# iMAgery Focused Therapy for PSychosis (iMAPS-2): An Assessor-blind Feasibility Randomized Controlled Clinical Trial

**DOI:** 10.1093/schbul/sbaf060

**Published:** 2025-10-06

**Authors:** Christopher D J Taylor, Ben Helliwell, Rebecca Coleman, Chris J Sutton, Paul Hutton, Yvonne Sylvestre, Leanne Bird, Gemma Shields, James A Kelly, Thomas Brandwood-Spencer, Alicia Boland, Emma Sharrock-Ingham, Bukunmi Babatunde, Amy Beech, Anvita Vikram, Serena Guillemard, Arnoud Arntz, Sean F Harper, Katherine Berry

**Affiliations:** School of Psychology, Faculty of Science, The University of Sheffield, Sheffield, United Kingdom; Pennine Care NHS Foundation Trust, Greater Manchester, United Kingdom; Pennine Care NHS Foundation Trust, Greater Manchester, United Kingdom; Pennine Care NHS Foundation Trust, Greater Manchester, United Kingdom; Lancashire Clinical Trials Unit, University of Central Lancashire, Preston, United Kingdom; School of Health & Social Care, Edinburgh Napier University, Edinburgh, United Kingdom; Division of Population Health, Health Services Research and Primary Care, School of Health Sciences, The University of Manchester, Manchester Academic Health Science Centre, Manchester, United Kingdom; Division of Psychology and Mental Health, School of Health Sciences, The University of Manchester, Manchester Academic Health Science Centre, Manchester, United Kingdom; Greater Manchester Mental Health NHS Foundation Trust, Manchester, United Kingdom; Manchester Centre for Health Economics, Division of Health, Health Services Research and Primary Care, The University of Manchester, Manchester Academic Health Science Centre, Manchester, United Kingdom; Greater Manchester Mental Health NHS Foundation Trust, Manchester, United Kingdom; Faculty of Health & Medicine, Lancaster University, Lancaster, United Kingdom; Pennine Care NHS Foundation Trust, Greater Manchester, United Kingdom; Pennine Care NHS Foundation Trust, Greater Manchester, United Kingdom; Pennine Care NHS Foundation Trust, Greater Manchester, United Kingdom; Pennine Care NHS Foundation Trust, Greater Manchester, United Kingdom; Pennine Care NHS Foundation Trust, Greater Manchester, United Kingdom; Pennine Care NHS Foundation Trust, Greater Manchester, United Kingdom; Pennine Care NHS Foundation Trust, Greater Manchester, United Kingdom; Department of Clinical Psychology, University of Amsterdam, Amsterdam, Netherlands; Health Board Headquarters, NHS Grampian, Aberdeen, United Kingdom; Division of Psychology and Mental Health, School of Health Sciences, The University of Manchester, Manchester Academic Health Science Centre, Manchester, United Kingdom; Greater Manchester Mental Health NHS Foundation Trust, Manchester, United Kingdom

**Keywords:** mental imagery, psychosis, schemas, schematic beliefs, imagery focused therapy, schizophrenia

## Abstract

**Background and Hypothesis:**

Intrusive mental images and negative schematic beliefs have been identified as maintenance and possible causal factors for some psychotic experiences, with limited focus in existing therapies in psychosis. Our primary aim was to assess the feasibility and acceptability of undertaking a randomized controlled trial (RCT) of a novel, imagery focused psychological therapy for psychosis (iMAPS).

**Study Design:**

An assessor-blind RCT (iMAPS-2). Participants who were help seeking; with hallucinations or delusions, who reported distressing intrusive mental imagery were eligible to take part. Participants were randomly assigned (2:1) to receive 12 sessions of iMAPS therapy plus standard care or treatment as usual (TAU). Assessments were undertaken at 0, 16 and 28 weeks. The primary feasibility outcomes were recruitment target, retention at 16 week follow up and number of therapy sessions attended.

**Study Results:**

The trial recruitment was 100% of target (45 participants). The study had a high rate of retention of 80% (36 participants) at 16-week primary endpoint, a high rate of adherence to the imagery focused therapy (77%) and positive qualitative feedback. There were two serious adverse events in the iMAPS therapy arm deemed unrelated to treatment and zero in the TAU group.

**Conclusions:**

This is the largest trial to date of imagery focused therapy for psychosis, demonstrating it is safe. An adequately powered clinical and cost effectiveness trial is warranted to provide an estimate of the effects of the iMAPS therapy.

**Trial Registration ISRCTN:**

81150786.

## Introduction

Schizophrenia is a significant challenge internationally, with substantial human suffering, disability and financial costs (eg, £12.5 Billion a year in England alone).^1^ Cognitive behavioral therapy (CBT) has demonstrated a robust but small effect on hallucinations and delusions^1,2^ but it does not work for everyone.^2^ Up to 74% of people with psychosis experience distressing intrusive mental images^3,4^accompanied by high levels of negative schemas—strongly held beliefs about the self and others,^5^ which are associated with distress, poorer functioning and clinical outcomes. Despite this prevalence, there are few references to imagery work in existing CBT for Psychosis manuals.^6–8^

Distressing imagery is often sometimes associated with trauma and/or psychotic experiences. Adverse life experiences are frequently reported by people with psychosis^9^ and can lead to individuals developing negative beliefs about self and others, often with associated intrusive mental images (eg, flashbacks, “pictures in your mind’s eye”).^3^ Taylor et al.^10^ used a qualitative approach to explore core beliefs and schema in psychosis and their links with hallucinations and paranoia. Four emergent themes were identified including links between beliefs and images. However, the existing core belief techniques from standard CBT for depression or anxiety are frequently under-utilized by therapists working with psychosis (8% of sessions in largest CBT for Psychosis Trial).^11^

In recent years, there have been a small number of studies making use of imagery techniques in psychosis in individual single case studies or small case series. A recent systematic review of imagery interventions in psychosis confirms there are limited trials to date.^12^ In psychosis, Ison et al.^13^ conducted a small case series using imagery re-scripting alone to work with voices. Subsequently, other case series have focused on using imagery rescripting as a standalone technique for: voice hearers who have experienced trauma,^14^ nightmares,^15^ and imaginal reprocessing of traumatic experiences,^16^ which have led to reductions in distress, and reductions in conviction in beliefs associated with images. Imagery approaches can also include the use of positive imagery techniques^17^ to help the person feel good, positive, and compassionate, which are also often missing from existing treatment manuals.

iMAgery focused therapy for Psychosis (iMAPS)^18^ involves an assessment, utilizes an imagery formulation model, and a range of imagery techniques including imagery manipulation (eg, changing the perceptual characteristics), imagery rescripting (changing the associated meaning and narrative content) and positive imagery. It has previously been tested in a small case series (*N* = 5) and was acceptable to participants with psychosis (measured by session uptake, feedback, etc.).^19^ The recruitment target was achieved and excellent uptake of sessions (100% of sessions attended) and good retention (100% retention during therapy). There were no serious adverse events or adverse effects. A replication case series^20^ (*N* = 5), delivered entirely online via telehealth video therapy sessions, with a more culturally diverse group of participants was also acceptable with 100% attendance at sessions and no serious adverse events. However, the overall sample size across the two case series was small (*N* = 10), with an absence of a control group and assessor blinding. In the current study, our main aim was to assess whether it is feasible to conduct a future randomized controlled trial (RCT) to examine the (clinical and cost) effectiveness of an imagery focused psychological therapy in psychosis. Although a feasibility study, it is the largest RCT of an imagery focused therapy in schizophrenia and psychosis to date.

## Methods

### Design

A single-center rater blind, two-arm, randomized controlled feasibility trial, recruiting individuals at a UK National Health Service (NHS) mental health trust at a single site in Greater Manchester.

### Procedure

This trial, named the iMAgery focused therapy for Psychosis (iMAPS-2) trial, was approved by the UK Health Research Authority (HRA) Yorkshire and The Humber Leeds West Research Ethics Committee on May 6, 2022 (Reference 22/YH/0091). The trial was prospectively registered at ISRCTN 81150786. The protocol^21^ was approved by a predominately independent Trial Steering Committee (TSC) and uploaded at Open Science Framework (https://osf.io/pvaz3) before the trial began and the most recent version is provided in the Supplementary Materials. Three substantial ethical amendments were made in two amendment submissions: (i) to lower recruitment age from 18 to 16 years due to a number of potentially eligible people aged 16-18 being referred from EI teams; (ii) to expand recruitment to inpatient wards; and (iii) the addition of a therapist qualitative study and are described in the Supplementary Materials.

### Participants

We approached the early intervention psychosis (EIP) teams and community mental health teams (CMHT) and asked staff to reviewed caseloads against the study eligibility criteria and talk to potential participants about taking part. The research team then discussed the study with those who were interested. Participants gave written informed consent and were then screened via an interview using the Positive and Negative Syndrome Scales^22^ (PANSS) and an adapted imagery interview^23^ asking about intrusive mental images linked to either hallucinations and/or delusions. The 16-week and 28-week assessments were conducted by research assistants blind to trial arm allocation. Assessments took place at NHS clinic spaces, or at participants home or a mixture of face to face or telephone and video (MS TEAMS) calls, depending on participant preference.

Individuals who met the following criteria were eligible to take part: (i) meeting criteria for an ICD-10 schizophrenia-spectrum diagnosis (confirmed by the participant’s psychiatrist, a case note review and an ICD standardised checklist) or meeting the operational criteria to under the care of early intervention psychosis team, defined using the Positive and Negative Syndrome Scales (PANSS) and/or the Comprehensive Assessment of At Risk Mental States^24^ (CAARMS) psychosis transition criteria; meeting a criterion level of positive symptoms indicated by a PANSS score of > 3 on either delusions (P1), hallucinations (P3), grandiosity (P5) or suspiciousness (P6) in the previous week; (ii) aged 16 and above; (iii) identifying a distressing image^1^ (rated 50% distressing or above) related to the psychotic experience scoring 3 or above on PANSS; the participants self-reported the image as distressing (eg, *Have you had a distressing image over the past week/month? Yes/No; What would you rate the distress over the past month from 0-100?*); (iv) capacity to give informed consent; and (v) under the care of an NHS mental health team the study is recruiting from with a keyworker/access to a duty team worker.

Exclusion criteria were (i) primary diagnosis of alcohol, substance misuse disorder, or bipolar disorder (affective psychosis); (ii) secondary presenting difficulties such as severe addiction, acute suicidal risk, dementia, neurological disorder; (iii) developmental disability (moderate to severe learning difficulty); (iv) acquired brain injury/organic syndrome; (v) currently participating in physical or mental health treatment studies or receiving psychological therapy; (vi) unable to complete the measures in written English (due to assessment battery psychometric validation in English); (vii) in forensic settings; and (viii) unmanageable level of risk of violence to researchers/clinicians (eg, harassment behavior—stalking).

### Sample Size

As a feasibility trial, the sample size was chosen to estimate recruitment and retention parameters, in addition to the SD for outcome measures, with reasonably good precision. Recruiting 45 participants enabled us to estimate the retention rate at end of therapy via a 95% (exact binomial) confidence interval, with width no greater than 25%, assuming retention would be at least 80%. It was also sufficient for the estimation of the SD, although the sample size (expected minimum 36 participants with outcome data) is toward the lower end of the sample size recommended.^25^

### Randomization and Masking

Participants were randomly allocated on a 2:1 ratio to receive either usual care (TAU) or iMAPS plus TAU using random permuted blocks of random length (6; 9), stratified by team (EIP or CMHT). The allocation sequence was computer generated, uploaded and stored by a statistician independent of the study team (AK) into the independent randomization system provided by Sealed Envelope (https://www.sealedenvelope.com/). The statistical analyses were undertaken by YS, with support from CS. Delegated staff at site confirmed the eligibility criteria before randomizing participants. Allocation concealment was ensured, as the service released the randomization codes after participants were recruited into the trial, which took place after baseline measurements were completed. Methods to maintain masking included arrangements for separate telephone numbers and different generic email addresses for research assistants and therapists, and verbal reminders to participants, family members, and care team clinicians about the importance of masking. Breaks in allocation concealment were reported to the Chief Investigator, with learning points disseminated to the study team and to the independent trial steering committee for monitoring and as detailed below, an assessor blind to treatment allocation completed the assessment and scoring.

### iMAgery Focused Therapy for Psychosis (iMAPS)

Participants allocated to receive iMAPS therapy were offered up to 12 one-hour sessions of individual imagery focused therapy for psychosis by appropriately trained therapists over a 4-month period plus their usual care (offered treatment in line with UK national clinical guidelines from the EIP and CMHT teams). iMAPS therapy sessions were typically once per week, but with flexibility offered. The therapy was based on the iMAPS therapy approach (Taylor et al. 2019), expanded from 6 to 12 sessions for this trial based on an earlier case series^19^ and informal feedback from patients that this would be helpful. The intervention was delivered by clinical psychologists and CBT therapists who met British Association for Behavioral and Cognitive Psychotherapies (BABCP) minimum training standards for cognitive behavioral therapy. The therapy sessions were delivered either face to face in person in NHS clinic bases, at home or online via MS TEAMS. Therapists had an initial 2-day training (led by CT) and undertook additional relevant training as required.

In the initial phase of therapy, patients and therapists collaboratively identified an intrusive image they wished to work on, associated with a psychotic symptom (eg, hallucination or delusion), usually the image which met inclusion criteria to enter the trial. The intrusive image chosen was collaboratively formulated using the iMAPS model into an idiosyncratic formulation focusing on the image, appraisal, power of the image, coping strategies and past experiences, including development and influence of core schematic beliefs. Subsequent sessions focused on imagery change strategies outlined in our therapy guide,^18^ such as imagery manipulation, imagery rescripting of past events and flashforwards, working with nightmares, working with positive imagery, and a final phase focused on consolidation.

### Therapy Fidelity

Therapy sessions were recorded for supervision purposes and fidelity assessment. Therapy fidelity was independently assessed in a subsample of recordings using the Cognitive Therapy Scale for Psychosis^26^ (CTS-Psy) by a BABCP accredited CBT Therapist, experienced in working with psychosis. Therapy fidelity was facilitated via a therapy guide, fortnightly group clinical supervision, individual supervision, and assessment of video/audio recorded therapy sessions using the CTS-Psy.^26^ Following each session, therapists complete a session record that monitored the content of the session, in terms of the agenda, image focus, between session tasks and imagery change strategies used. These data were used to monitor adherence and address any adherence difficulties with therapist training sessions.

### TAU

Participants randomized to the treatment as usual arm were offered treatment in line with UK national clinical guidelines from the EIP and CMHT teams. Clinical records and case notes were reviewed to monitor the offer of care received by participants and we documented which proportion of usual care arm participants received CBT or any other psychological intervention during the trial or in the iMAPS + TAU group. For both therapy and TAU groups, we collected data on any additional therapy offered via self-report and medical record screening at follow up visits at 16 weeks and 28 weeks.

### Outcomes and Assessments

#### Feasibility Outcomes

The trial had three feasibility outcomes: (i) recruitment of EI and CMHT participants into a trial of imagery focused therapy for psychosis, (ii) levels of trial retention at 16-week follow-up (proposed primary outcome), (iii) the level of engagement of EI and CMHT patients in the iMAPS therapy. In addition, our trial registration lists additional feasibility outcomes of (iv) therapist adherence to therapy protocols, and (v) therapy safety—number of serious adverse events (SAEs) and adverse events (AEs).

Recruitment, retention and therapy engagement were defined in advance using a “traffic light” approach with three levels^27^ with thresholds to indicate for each outcome if a larger-scale, well-powered future clinical trial would appear feasible using the current study design (Green), if modifications to the current trial design would be required (Amber) or if there may be unresolvable issues that would suggest a future trial would be difficult to successfully conduct (Red).

These progression criteria were reviewed during the peer review of the funding application, by the ethics committee and by the TSC and are documented in the protocol (Supplementary Material). These were:

1. Recruitment of participants into a trial of iMAPS: Green: >=80% (*n*>=36) of target recruited; Amber >=40 % (*n* = 18-35)-< 80% of target recruited; RED < 40% (*n* < 18) of target recruited.
2. Retention of participants: Green: >=80% of participants providing 16-week outcome data; Amber > 60%- < 80% of participants providing 16-week outcome data; Red: < 60% of participants providing 16-week outcome data.
3. Levels of engagement in iMAPS therapy (Adherence): Green>=75% adherence of participants attend at least 5 out of 12 sessions of therapy, Amber>=40% participants attend at least 5 out of 12 sessions of therapy; Red: < 40% of participants attend at least 5 out of 12 sessions of therapy (based on Jolley et al.^28^).

### Rater Blind Measures and Self-report Measures

At assessment, we collected demographic information and clinical details (such as diagnosis, previous therapy, current service support). A brief interview regarding images and imagery was conducted with participants also completing an imagery characteristics visual analogue scale used in previous studies^19,29^ and a brief measure of core beliefs.^30^ Participants were asked to describe and rate up to three distressing intrusive images and the frequency (weekly; monthly) and distress (0-100 where 0 is no distress at all and 100 is worst distress). To examine the feasibility of utilising potential interview and self-report measures and gather participants’ feedback on these, we administered these at baseline, 16-week and 28-week assessments. All research assistants received training and ongoing supervision regarding the administration and scoring of the rater blind semi structured interview measures and demonstrated excellent reliability against gold standard expert raters ratings (ICC: 0.91).

The measures utilised were:

The Positive and Negative Syndrome Scales^22^ (PANSS), a frequently used semi-structured interview measure assessing the presence and severity of psychotic symptoms and other mental health symptoms. Each PANSS item is scored using a 7-point scale, where 1 indicates symptoms absent and 7 indicates extreme severity of symptoms. The PANSS is often reported as a total score and the positive, negative and general symptoms. In line with recent factor analysis of PANSS,^31^ we also report the subscales in relation to following symptoms: positive, negative, excitative, affective, and cognitive disorganization.

The Psychotic Symptom Rating Scales^32^ (PSYRATS) is a widely utilized semi-structured interview measure which assess dimensional aspects of auditory hallucinations (PSYRATS-AH) with 11 items and dimensional aspects of delusions (PSYRATS-DEL) with six items. The items are rated over the past week and scored on a five-point scale, with zero indicating not present to four, indicating most severe.

The Questionnaire about the Process of Recovery^33^ (QPR) is a self-report measure with 15 items and assess intrapersonal recovery based on work with individuals with lived experience of psychosis.^34^ QPR is scored on a five-point scale, with zero indicating disagree strongly and four indicating agree strongly. Higher scores on this measure suggest greater perceived intrapersonal recovery.

The Brief Core Schema Scales^30^ (BCSS) is a 24-item self-report questionnaire assessing core negative and positive schematic beliefs regarding the self and others. Higher scores reflect a greater endorsement of beliefs. The items are first endorsed as either present or absent (Yes/No), with endorsed beliefs rated on a five-point scale from zero to four.

The Trauma and Life Events checklist^35^ (TALE) is a 20 item screening questionnaire to assess for exposure to potentially traumatic events, with excellent psychometrics.^36^

The International Trauma Questionnaire^37^ (ITQ) is a 18-item measure assessing post-traumatic experiences and symptoms in the past month. ITQ has six items which give a dimensional PTSD score and a dimensional disturbances in self-organization score (DSO), measuring the symptoms of C-PTSD. It is possible to categorize individuals with probable PTSD or C-PTSD according to ICD-11 criteria.^37^

The Basic Emotions Scale^38^ (BES) is a 20-item self-report measure of the five “basic” emotions (anger, sadness, disgust, fear, and happiness) over the past week. It has a seven-point Likert scale to rate the degree to which specific emotions have been experienced, with a total score of each emotion scale.

The Beck Anxiety Inventory^39^ (BAI) is a 21-item self-report measure of common symptoms of anxiety. Scores rate from 0 not at all to 4 severely—I could barely stand it. Higher scores indicate more severe anxiety symptoms.

The Calgary Depression Scale^40^ (CDS) is a nine-item informant rated interview measure which assesses depression in schizophrenia, distinguishing depression features from negative symptoms of psychosis.

The Warwick-Edinburgh Mental Well-being Scale^41^ (WEMWBS) is a 14-item self-report scale assessing positive mental flourishing and well-being over the past 2 weeks. Each item is rated on one (none of the time) to five (all of the time) scale.

The Personal and Social Performance Scale^42^ (PSP) is an interview measure of functioning in (i) socially useful activities, (ii) personal and social relationships, (iii) self-care, (iv) disturbing and aggressive behaviors. It scores severity of difficulties in each area, rated on a six-point scale measuring level of functioning, from one absent to six very severe. The scores are then summarized to give a score out of 100, with higher scores indicating better functioning.

The EQ-5D-5L^43^ is a self-report questionnaire of health in five areas—physical mobility, self-care, usual activities, pain-discomfort and anxiety, and depression. A visual analogue scale asks participants to rate their own health from zero (poorest health) to 100 (best health).

The Recovering Quality of Life^44^ (ReQOL-10) scale is a generic mental health measure of quality of life which includes themes of connectedness, hope, and optimism about the future.

Economic Patient Questionnaire (EPQ)^45^ is used to collect data from participants regarding the range and frequency of health and social care services used. These three of measures (EQ-5D, ReQoL-10, and EPQ) were used to inform a future cost-effectiveness analysis if progression criteria met as part of a larger future trial.

The Working Alliance Inventory^46,47^ (WAI) is a self-report measure of therapeutic alliance with client and therapist versions, administered at sessions 3, 6, and 9 by a member of team who was not the participant’s own therapist. The client version utilized has 12 items on a 1 to 5 scale. The therapist version utilised has 10 items on a 1 to 5 scale. This assesses therapeutic alliance on the basis of Bordin’s three theoretical components of alliance: goals, tasks and bond.^48^ The participant questionnaires were administered over phone or video call at separate appointments, by a different therapist to whom they were seeing for sessions.

A brief imagery interview schedule adapted from an imagery interview previously used in social phobia^23^ was also utilized. Imagery Visual Analogue Scales named Mental Imagery in Psychosis Questionnaire (MIPQ) were also completed. These were visual analogue scales rated by the participant on a scale 1-10 from “not at all” to “extremely,” including five questions: “*How compelling was the image*?,” “*How real was the image?*,” “*How vivid was the image?*,” “*How absorbing was the image*?” and “*How preoccupying was the image?*.” This was based on an earlier version of a mental imagery questionnaire developed by Holmes et al.^29^ and used in previous iMAPS studies.^19,20^ In addition, two imagery ratings were also assessed at each visit “*To which extent could you understand the role that the image(s) play in changing your fears that other mean you harm?*” and “*To what extent could you find helpful/positive ways of coping with your images?.”* We also administered a new Psychosis Imagery Questionnaire (PIQ) assessing frequency of images in relation to specific psychotic symptoms, which is under development as part of the trial.

### Adverse Events (Safety)

We administered a range of other measures to detect any evidence of harm or threats to acceptability following an adapted version of a previously utilised protocol.^49^ In line with good clinical practice and the UK Health Research Authority^50^ and the International Council for Harmonisation,^50,51^ we recorded all participant adverse events, which were monitored and reviewed by a senior clinician researcher to examine seriousness.

The severity, relatedness to trial procedures and or interventions and expectedness were all assessed. All potential SAEs were independently assessed by the Independent Chair of TSC and if related, reported to the relevant regulator. Following Klinberg et al.^49,52^ we defined suicidal crisis without attempt as a score of 2 on item 8 of the CDSS. Severe symptom exacerbation was recorded if Clinical Global Impression Severity (CGI-S) Scale and Clinical Global Impression Improvement (CGI-I) scale ratings suggested the participant had become severely to extremely mentally unwell (ie, they scored 6 or 7 on the CGI-S) and their mental health problems were much or very much worse than they were at the start of the trial.^53,54^ Both the patient and researcher rated CGI-S are scored from 1 to 7 with higher scores indicating greater symptom severity. The CGI-I scales are scored 1 to 7 with higher scores indicating less improvement. We also planned to collect data on a bespoke measure of potential unwanted effects of trial participation (Adverse Experiences in Psychotherapy Questionnaire; AEP), used in other trials of psychological interventions for psychosis.^55^ Clinical notes were also reviewed as part of this process. We also undertook two qualitative studies, which will be reported elsewhere, using reflexive thematic analysis^56^ investigating participants’ experiences of taking part in the trial and therapist experiences of delivering the therapy.

### Data Analysis

A statistical and health economic analysis plan (SHEAP) was approved by the TSC and published online via ISRCTN prior to commencement of the analysis of unblinded outcome data. The feasibility of recruitment, retention, adherence, and study participants’ characteristics, were summarised using appropriate descriptive statistics. Overall retention rates and completion rates for individual outcome questionnaires were estimated using point estimates with 95% binomial CIs. Analyses to assess proof-of-concept and proof-of-efficacy were by “intention-to-treat (ITT).” Each clinical outcome was analyzed using a linear regression model at each timepoint (16 or 28 weeks) adjusted for team (CMHT or EI) and the corresponding baseline outcome score, using Maximum Likelihood Estimation (MLE). No imputation of missing outcome data was performed. However, to avoid exclusion of participants with missing baseline data for outcome variables in the complete-case analyses, we used simple mean imputation (across the groups) of the corresponding baselinesmall data.

Point estimates were presented as regression coefficients and two-sided 100*(1-α)% confidence intervals with α ranging from 0.05 to 0.25 (in steps of 0.05, following the approach proposed by Lee et al., 2014) for the between-groups differences in means for the candidate primary outcomes at the primary end-point (ie, 16 weeks). Standardized effect sizes (SES) were also estimated using the corresponding pooled within-group SD. Point estimates with 95% confidence intervals were presented for the 28-week outcomes for these measures, and for the other clinical outcomes.

We selected four potential candidate primary outcomes in the SHEAP: PANSS total score, PSYRATS, QPR, and BCSS. The first two are widely utilised measures of psychotic symptoms and often primary outcomes in other psychological therapy trials. QPR was chosen as it is a widely used measures of service user defined recovery. BCSS was chosen as imagery work can often target and change unhelpful strongly held beliefs which are distressing in psychosis. In line with current NICE recommendations, the mapping function developed by the Decision Support Unit (DSU) using the “EEPRU dataset” was used to estimate utilities for the EQ-5D data.^57^ Estimating utilities from ReQoL data were used to generate utilities using a selection of the items available and published preference weights.^58^

### 28 Week Follow-up

Due to an extension to recruitment period, it was not possible to offer follow up appointments to all 45 participants before the end of the trial. We were able to offer 28-week assessments to 39 of 45 participants.

### Missing Data

Simple mean imputation (across the groups) to avoid exclusion of such participants in the complete-case analysis. Please see the SHEAP for full details.

## Results

**Figure 1.**
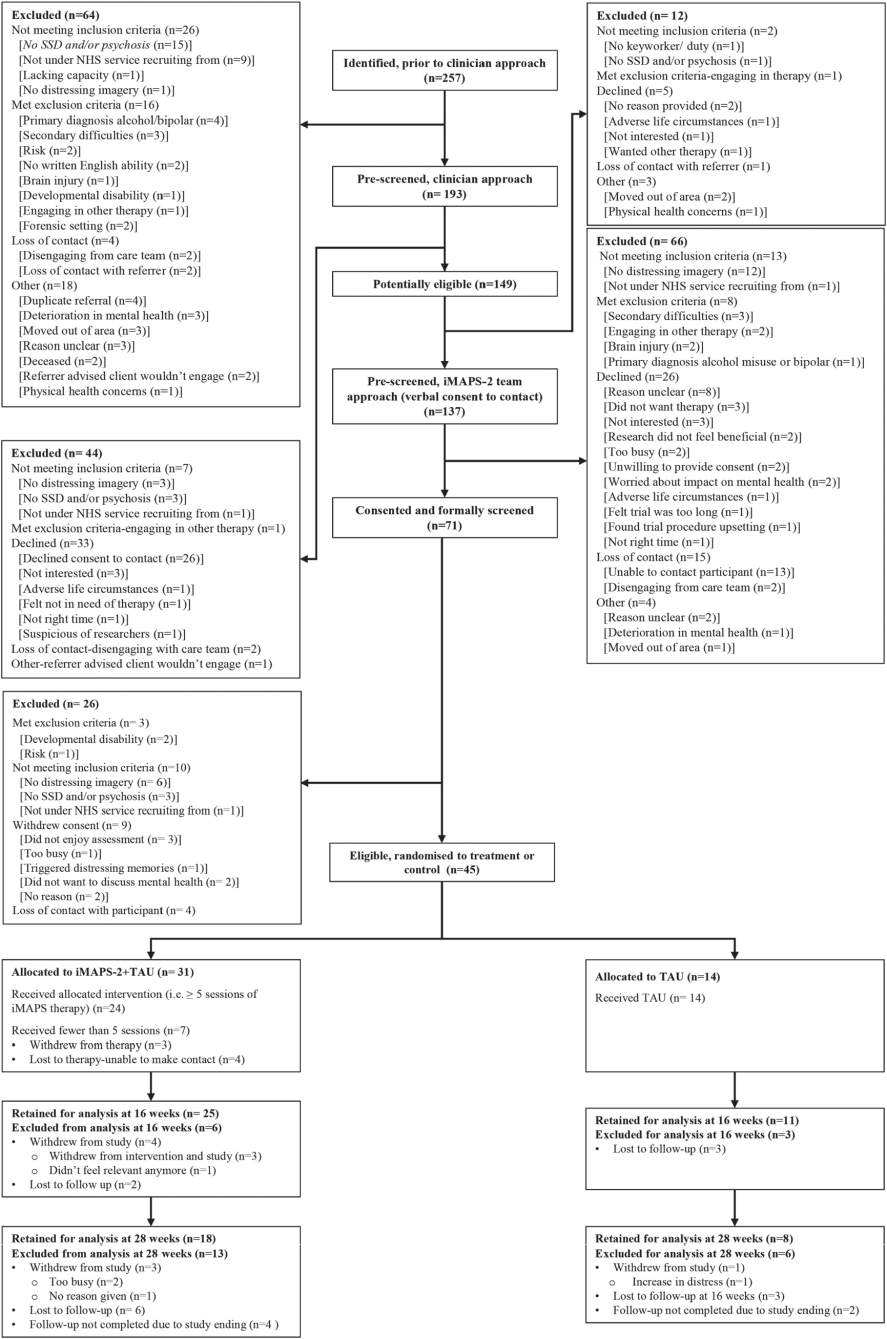
CONSORT diagram.

The CONSORT diagram is displayed in Figure 1. Between 14th June 2022 and 15th September 2023, 192 of 257 passed initial eligibility screening checks and were contacted by their clinical team to see if they were interested in taking part (Figure 1). Of this group, 137 service users were referred. We assessed eligibility for 71 individuals, with a 2:1 randomization, 45 participants were randomised to either iMAPS plus TAU (*n* = 31) or TAU alone (*n* = 14). Sixteen and 28week follow ups were completed until May 2024.

### Recruitment

We recruited 100% of our target sample of 45 participants (green progression criteria met). The monthly and cumulative randomization is given in Supplementary Table 1. Of the 71 who gave informed consent to be screened for eligibility, 26 were excluded. Three met the exclusion criteria (developmental disability *N* = 2; Risk *N* = 1), six did not report distressing mental imagery, three did not have a schizophrenia spectrum diagnosis, one was not under the care of an NHS service we were recruiting from, four we were unable to contact to assess, and nine withdrew for various reasons. Forty-five out 45 eligible and consenting participants took part in the trial. The recruitment window was extended for reasons outlined in the Supplementary Information, including staffing issues.

### Retention

Thirty-six participants (80%) completed the 16-week follow-up assessment (green progression criteria met); four withdrew from the study (no reason given *N* = 3; did not feel relevant taking part anymore *N* = 1) and five were lost to therapy/unable to make contact. Twenty-six out of 39 were offered (67%) completed 28-week assessments, with four withdrawing (too busy *N* = 2; no reason given *N* = 1), nine lost to follow up and six were not offered follow up assessment, due to trial ending. Trial retention was good across arms at 16-week assessment but reduced at 28-week assessment.

### Therapy Engagement

Thirty-one out of 45 participants were allocated to the intervention arm, of which 29 received the iMAPS therapy, with two participants not attending any sessions. Twenty four out of 31 iMAPS plus TAU participants (77%) received five or more sessions of 12 iMAPS sessions offered (green progression). Two participants did not receive any sessions of iMAPS and five participants received some sessions but dropped out before session five. Reasons for not attending or dropping out of therapy included “not wanting to take part anymore” and “too busy.” The average number of therapy sessions attended was 9.2 (SD = 4.0) and the median sessions attended was 12 (range 7-12). Participant engagement to the iMAPS therapy was defined as a participant attending five or more out of the 12 iMAPS therapy sessions. A total of 24/31 (77%, 95%CI 59-90) participants were adherent (attended at least 5 sessions of the iMAPS therapy; green progression criteria). The distribution of number of therapy sessions attended and their duration is showed in Supplementary Figure 1.

### iMAPS Fidelity

In line with our trial registration, fidelity to therapy delivery was a primary outcome and all therapists demonstrated acceptable or very good independently rated CTS-Psy^26^ scores across a sample of 25 sessions (9% of 267 sessions delivered); average rating 48.8 out of 60 (Range 34-57). One hundred per cent of sessions rated scored over 33.5/60, which was the highest mean score in the original CTS-Psy validation study.^26^

### Session Records Post Therapy Checklist

The 29 participants who engaged in therapy attended 267 sessions of iMAPS. Eighty-four per cent of sessions had a target image identified and being worked on in therapy. Ninety-six per cent of sessions had an agenda and 88% of sessions had a between session task agreed, with 77% of between session tasks completed. In summary, as outlined above, all three pre-specified progression criteria regarding recruitment, retention, and therapy engagement were all rated green, suggesting a fully powered randomised trial is feasible.

### Blind Breaks and Measure Completion

There were 12 blind breaks, where the assignment of participants was revealed to assessors during the 16-week or 28-week follow-ups. For each case, another rater masked to group allocation completed and scored the respective assessments when unblinding occurred. Measure completion rates were as follows: baseline (100%), 16 weeks (80%), and 28 weeks (66%).

### Rater Blinded and Self-Report Measures

The baseline demographic characteristics are reported in Table 1. Details of participants’ trauma history are provided in Supplementary Table 2, which highlights that a large number of participants reported repeated trauma (84%) and multiple exposures (98%) to traumatic events. These were also reported to have a high level of perceived impact of these events on their ongoing problems (*M *= 8.0; *S.D.* 2.0 out of a potential maximum score of 10). Descriptive statistics of the measures collected at the three time points are summarised in Table 2.

**Table 1.** Baseline Demographics and Clinical Characteristics of the Trial Participants

		iMAPS + TAU	TAU	Total
		*n* = 31 (%)	*n* = 14 (%)	*N* = 45 (%)
Gender	Male	19 (61)	5 (36)	24 (53)
	Female	11 (35)	8 (57)	19 (42)
	Non-binary	1 (3)	0 (0)	1 (2)
	Did not answer	0 (0)	1 (7)	1 (2)
Age	Mean (SD)	36 (14)	35 (12)	36 (13)
	Range	19-61	22-57	19-61
	Median (IQR)	34 (23-49)	31 (25-46)	32 (24-49)
PANSS—baseline	Not ill	7 (23)	3 (21)	10 (22)
	Mildly ill	19 (61)	7 (50)	26 (58)
	Moderately ill	4 (13)	4 (29)	8 (18)
	Markedly ill	1 (3)	0 (0)	1 (2)
*Diagnosis*				
ICD-10 Code				
	F20.0 (Schizophrenia)	8 (26)	3 (21.5)	11 (24)
	F20.9 (Unspecified schizophrenia)	1 (3)	2 (14)	3 (7)
	F23 (Acute and transient psychosis)	8 (26)	3 (21.5)	11 (25)
	F23.1 (acute polymorphic psychotic disorder w symptoms of schizophrenia)	1 (3)	0 (0)	1 (2)
	F25.2 (schizoaffective disorder; mixed type)	4 (13)	1 (7)	5 (11)
	F.29 Psychosis Not Otherwise Specified	0 (0)	1 (7)	1 (2)
	Meets entry criteria for an EIP service for first-episode psychosis at baseline.	9 (29)	4 (29)	13 (29)
Highest level of education	secondary school	8 (26)	2 (14)	10 (22)
	further education	18 (58)	7 (50)	25 (56)
	higher education	5 (16)	5 (36)	10 (22)
Employment status	Full-time	2 (6)	2 (14)	4 (9)
	Part-time	7 (23)	1 (7)	8 (18)
	Student	3 (10)	1 (7)	4 (9)
	Unemployed	19 (61)	9 (64)	28 (62)
	Missing	0 (0)	1 (7)	1 (2)
Marital status	Single	26 (84)	11 (79)	37 (82)
	Married	0 (0)	1 (7)	1 (2)
	Living with partner	3 (10)	1 (7)	4 (9)
	Separated	1 (3)	0 (0)	1 (2)
	Divorced	1 (3)	1 (7)	2 (4)
Living arrangements	Spouse/partner	1 (3)	1 (7)	2 (4)
	Spouse/partner & children	2 (6)	1 (7)	3 (7)
	Spouse/partner & others	0 (0)	1 (7)	1 (2)
	Alone	11 (35)	3 (21)	14 (31)
	Parent/s only	10 (32)	4 (29)	14 (31)
	Supported accommodation/hostel	2 (6)	2 (14)	4 (9)
	Parent/s & siblings	5 (16)	1 (7)	6 (13)
	House share	0 (0)	1 (7)	1 (2)
Index of Multiple Deprivation	1-3	16 (52)	6 (43)	22 (49)
	4-7	6 (19)	6 (43)	12 (27)
	8-10	3 (10)	2 (14)	5 (11)
	Missing	6 (19)	0 (0)	6 (13)
Ethnicity	White British	25 (81)	12 (86)	37 (82)
	White Irish	1 (3)	0 (0)	1 (2)
	Pakistani	2 (6)	2 (14)	4 (9)
	Other Asian background	1 (3)	0 (0)	1 (2)
	African	1 (3)	0 (0)	1 (2)
	White & Black Caribbean	1 (3)	0 (0)	1 (2)
*Therapy received*				
Previous	CBT	4 (13)	2 (14)	6 (13)
	CBT P	4 (13)	0 (0)	4 (9)
	Counselling	1 (3)	1 (7)	2 (4)
	IPT	1 (3)	0 (0)	1 (2)
	Eclectic Psychotherapy & IPT	0 (0)	1 (7)	1 (2)
	CBT & DBT	1 (3)	0 (0)	1 (2)
	CBT, Family Intervention & CAT	0 (0)	1 (7)	1 (2)
	CBT & CBT P	1 (3)	1 (7)	2 (4)
	CBT P & Family Intervention	0 (0)	1 (7)	1 (2)
	CBT & Schema therapy	1 (3)	0 (0)	1 (2)
	none	18 (58)	7 (50)	25 (56)
Current (during trial)	CBT	2 (6)	3 (21)	5 (11)
	Family Intervention	1 (3)	1 (7)	2 (4)
	CBT P, DBT & Family Intervention	0 (0)	1 (7)	1 (2)
	CBT P & Counselling	1 (3)	0 (0)	1 (2)
	none	27 (87)	9 (64)	36 (80)
Service^*^	CMHT	12 (39)	6 (43)	18 (40)
	EI	19 (61)	8 (57)	27 (60)
Patient status	Inpatient	2 (6)	0 (0)	2 (4)
	Outpatient	29 (94)	14 (100)	43 (96)
Beck Anxiety Inventory (BAI)	Minimal anxiety	2 (6)	1 (7)	3 (7)
	Mild anxiety	5 (16)	0 (0)	5 (11)
	Moderate anxiety	6 (19)	5 (36)	11 (24)
	Severe anxiety	18 (58)	8 (57)	26 (58)

^*^ *Community Mental Health Team (CMHT), Early Intervention Psychosis Team (EI).*

**Table 2.** Outcome Measures at Baseline, 16 Week Follow-up, and 28-week Follow-up by Randomized Group

	iMAPS + TAU (*n* = 31)			TAU (*n* = 14)		
	*M*	SD	Missing	*M*	SD	Missing
PANSS Total						
Baseline	65.4	11.5	0	66.6	9.1	0
16 weeks	60.7	13.8	9	66.7	11.9	3
28 weeks	61.1	15.1	15	64.4	18.8	9
PANSS Positive						
Baseline	15.6	4.0	0	17.1	2.7	0
16 weeks	12.0	4.3	8	15.5	4.4	3
28 weeks	11.5	4.9	13	13.7	5.8	8
PANSS Negative						
Baseline	15.0	4.9	0	13.3	3.0	0
16 weeks	14.8	5.0	8	14.1	2.8	3
28 weeks	15.5	6.0	14	12.0	3.4	8
PSYRATS-AH						
Baseline	13.9	14.1	0	18.5	13.5	0
16 weeks	11.1	13.6	8	12.1	14.0	3
28 weeks	12.1	14.0	14	7.6	12.0	8
PSYRATS-DEL						
Baseline	13.8	5.2	0	15.9	3.2	0
16 weeks	9.9	6.9	8	15.1	4.6	4
28 weeks	6.2	7.0	14	11.8	5.0	8
QPR						
Baseline	28.7	11.5	1	27.7	9.5	0
16 weeks	36.0	9.5	9	31.4	11.0	3
28 weeks	34.8	8.9	17	35.5	12.3	8
Imagery Characteristics (MIPQ) Image 1						
Baseline	34.4	14.0	1	38.2	6.7	0
16 weeks	23.5	16.2	11	28.0	13.4	3
28 weeks	20.4	16.2	13	18.4	13.6	9
BCSS negative-self						
Baseline	8.8	7.4	0	9.4	7.2	0
16 weeks	5.9	5.5	11	8.1	6.0	4
28 weeks	5.1	5.0	13	7.0	6.5	7
BCSS positive-self						
Baseline	8.4	7.0	0	8.1	4.5	0
16 weeks	7.7	4.9	11	8.9	3.9	4
28 weeks	9.2	5.3	13	14.4	6.8	7
BCSS negative-other						
Baseline	9.9	7.4	0	9.3	8.1	1
16 weeks	9.5	6.7	9	8.7	7.6	4
28 weeks	8.6	5.6	13	5.7	5.7	7
BCSS positive-other						
Baseline	8.3	6.9	2	9.5	6.0	0
16 weeks	9.4	7.1	11	13.1	6.5	4
28 weeks	10.3	5.9	13	11.7	6.4	7
ITQ PTSD						
Baseline	14.6	6.4	4	12.2	5.4	1
16 weeks	11.9	6.3	11	10.6	4.7	4
28 weeks	11.4	7.4	16	8.0	6.1	8
ITQ DSO						
Baseline	15.6	6.5	4	12.5	4.9	1
16 weeks	11.9	6.2	11	11.1	7.0	3
28 weeks	11.1	7.0	16	6.5	4.4	8
BES—Anger						
Baseline	16.6	5.5	0	15.6	4.6	0
16 weeks	14.4	4.8	9	14.5	5.0	3
28 weeks	13.8	5.0	15	10.2	3.3	8
BES—Sadness						
Baseline	15.3	5.9	0	14.4	6.2	0
16 weeks	13.9	5.9	9	12.5	5.4	3
28 weeks	13.1	4.9	15	7.7	2.3	8
BES—Disgust						
Baseline	16.0	6.6	0	15.7	7.2	0
16 weeks	13.5	5.8	9	13.9	6.1	3
28 weeks	13.4	4.9	15	9.2	4.3	8
BES—Anxiety						
Baseline	20.4	6.1	0	21.1	5.0	0
16 weeks	18.0	5.2	9	18.6	5.3	3
28 weeks	18.5	4.7	15	15.3	5.4	8
BES—Happiness						
Baseline	14.8	4.6	0	15.8	3.0	0
16 weeks	15.3	3.8	9	15.5	5.0	3
28 weeks	15.9	3.8	15	16.0	6.7	8
BAI						
Baseline	30.3	16.2	0	31.3	14.6	0
16 weeks	26.7	14.8	9	23.9	15.3	3
28 weeks	26.8	15.6	8	20.6	7.4	7
CDSS						
Baseline	9.7	5.2	0	9.9	3.5	0
16 weeks	8.2	4.9	9	8.7	5.9	4
28 weeks	6.7	4.3	16	6.0	5.7	8
WEMWBS						
Baseline	37.0	9.8	0	36.6	8.1	0
16 weeks	41.6	8.4	9	37.9	9.6	3
28 weeks	43.2	12.1	16	39.8	12.1	8
PSP						
Baseline	58.0	9.8	0	56.2	8.1	1
16 weeks	60.0	13.2	9	58.3	12.2	3
28 weeks	55.5	10.8	16	70.6	15.6	9
EQ-VAS						
Baseline	53.5	17.1	0	52.5	15.3	0
16 weeks	58.7	18.7	8	60.9	11.6	3
28 weeks	55.0	20.7	14	61.3	22.7	6
ED-5D Index						
Baseline	0.6	0.2	1	0.5	0.3	1
16 weeks	0.7	0.2	11	0.5	0.4	4
28 weeks	0.6	0.2	15	0.4	0.4	7
ReQoL-UI						
Baseline	0.7	0.2	1	0.7	0.2	0
16 weeks	0.8	0.1	8	0.8	0.2	3
28 weeks	0.8	0.1	15	0.8	0.2	7

We estimated the potential effectiveness on a range of candidate primary outcome measures outlined above (ie, PANSS, PSYRATS, QPR, and BCSS) and their standard deviations (SDs). The results are presented in Table 3 with a range of confidence intervals, and the corresponding standardised effect sizes (with standardised 95% confidence intervals), where standardisation was performed using the pooled within-group standard deviation.

**Table 3.** Treatment Effects at 16 Weeks for the Possible Candidate Primary Outcomes

	Scale range	*N*	Effect estimate	in favor of …	95% CI	90% CI	85% CI	80% CI	75% CI	SD_pooled_	SES (95%CI)
**Positive and Negative Syndrome Scale (PANSS)**											
Total score	30-210	34	3.0	TAU	(−4.4 to 10.3)	(−3.2 to 9.1)	(−2.5 to 8.4)	(−1.9 to 7.8)	(−1.4 to 7.3)	13.2	0.2 (−0.3 to 0.8)
**Psychotic Symptom Rating Scales (PSYRATS)**											
Auditory Hallucinations Subscale (AHS)	0-44	35	−1.9	iMAPS	(−9.0 to 5.2)	(−8.2 to 4.3)	(−7.2 to 3.3)	(−6.6 to 2.7)	(−6.3 to 2.4)	14.7	−0.1 (−0.6 to 0.4)
Delusions Subscale (DS)	0-24	34	2.6	TAU	(−1.2 to 6.3)	(−0.6 to 5.7)	(−0.2 to 5.3)	(0.1 to 5.0)	(0.4 to 4.8)	6.3	0.4 (−0.2 to 1.0)
**Questionnaire about the Process of Recovery (QPR)**	0-60	34	−2.2	TAU	(−6.6 to 2.2)	(−5.8 to 1.5)	(−5.4 to 1.1)	(−5.0 to 0.7)	(−4.7 to 0.4)	10.0	−0.2 (−0.7 to 0.2)
**Brief Core Schema Scale (BCSS)**											
Negative-self	0-24	31	1.7	TAU	(−2.2 to 5.5)	(−1.6 to 4.9)	(−1.2 to 4.5)	(−0.8 to 4.2)	(−0.6 to 3.9)	5.7	0.3 (−0.4 to 1.0)
Positive-self	0-24	31	1.8	iMAPS	(−1.0 to 4.6)	(−0.6 to 4.1)	(−0.3 to 3.8)	(−0.1 to 3.6)	(−0.1 to 3.4)	4.7	0.4 (−0.2 to 1.0)
Negative-others	0-24	33	0.5	TAU	(−3.1 to 4.0)	(−2.6 to 3.5)	(−2.2 to 3.1)	(−1.9 to 2.8)	(−1.7 to 2.6)	7.0	0.1 (−0.5 to 0.6)
Positive-others	0-24	31	2.9	iMAPS	(−1.1 to 6.8)	(−0.5 to 6.2)	(−0.1 to 5.8)	(0.2 to 5.5)	(0.5 to 5.2)	6.9	0.4 (−0.2 to 1.0)

*Effect estimate = iMAPS intervention + TAU—TAU*.

*Standardised effect size (SES) = Effect estimate /* SD_pooled_; the corresponding 95%CI is calculated by dividing the limits of the 95%CI for the effect estimate by the SD_pooled_.

The effect sizes for the positive-self and positive-other subscales of the Brief Core Schema Scale (BCSS) were above a suggested minimally clinically important difference (MCID) of a standardised effect size of (SES) 0.3. However, the confidence intervals include zero and the estimated effects for the negative-self and negative-other subscales were in favor of the TAU. Table 4 shows the treatment effects at 12 and 28 weeks for the proof-of-concept outcomes with 95% confidence intervals, where there is promising improvement in many of the outcomes at 28 weeks (although there was higher attrition).

**Table 4.** Treatment Effects at 16 Weeks and 28 Weeks for All Outcomes, Results Generated from Regression Models

		16 Weeks				28 Weeks			
	Scale range	*N*	Effect estimate	95% CI	… in favor of …	*N*	Effect estimate	95% CI	… in favor of …
**Positive and Negative Syndrome Scale (PANSS)**									
Positive Symptoms	5-35	35	1.3	(−0.9 to 3.6)	TAU	24	−1.4	(−4.9 to 2.0)	iMAPS
Negative Symptoms	8-56	35	0.3	(−2.3 to 2.8)	TAU	23	−1.6	(−5.8 to 2.5)	iMAPS
Disorganization	8-56	35	0.7	(−1.6 to 2.9)	TAU	25	1.7	(−0.7 to 4.1)	TAU
Affect	7-49	35	1.4	(−1.7 to 4.6)	TAU	23	2.3	(−1.2 to 5.8)	TAU
Resistance	4-28	36	0.8	(−0.1 to 1.8)	TAU	25	−0.5	(−1.5 to 0.4)	iMAPS
Total score	30-210	34	3.0	(−4.4 to 10.3)	TAU	21	−1.1	(−13.2 to 11.0)	iMAPS
**Psychotic Symptom Rating Scales (PSYRATS)**									
Auditory Hallucinations Subscale (AHS)	0-44	35	−1.9	(−9.0 to 5.2)	iMAPS	23	−3.8	(−12.6 to 5.1)	iMAPS
Delusions Subscale (DS)	0-24	34	2.6	(−1.2 to 6.3)	TAU	23	3.2	(−2.2 to 8.6)	TAU
**Questionnaire about the Process of Recovery (QPR)**	0-60	34	−2.2	(−6.6 to 2.2)	TAU	20	2.4	(−4.1 to 8.8)	iMAPS
**Visual Analogue Scale (VAS) Imagery Characteristics (MIPQ)**									
Image 1	7-50	32	−1.5	(−10.0 to 7.1)	iMAPS	23	−4.3	(−18.3 to 9.7)	iMAPS
Item 6: To which extent could you understand the role that the image(s) play in changing your fears that others mean you harm?	0-10	31	0.6	(−1.7 to 2.9)	TAU	23	−0.2	(−3.6 to 3.2)	iMAPS
Item 7: To which extent could you find positive / helpful ways of using the image(s)?	0-10	31	−0.9	(−2.9 to 1.2)	iMAPS	23	−0.2	(−2.2 to 1.7)	iMAPS
Image 2	7-50	22	6.5	(−8.5 to 21.4)	TAU	15	−7.5	(−23.6 to 8.6)	iMAPS
Item 6: To which extent could you understand the role that the image(s) play in changing your fears that others mean you harm?	0-10	22	−0.4	(−3.5 to 2.7)	iMAPS	15	−1.2	(−5.3 to 2.9)	iMAPS
Item 7: To which extent could you find positive / helpful ways of using the image(s)?	0-10	22	−0.7	(−2.8 to 1.3)	iMAPS	15	−1.5	(−2.7 to −0.3)	iMAPS
Image 3	7-50	8	4.0	(−19.6 to 27.6)	TAU	7	−8.2	(−18.6 to 2.2)	iMAPS
Item 6: To which extent could you understand the role that the image(s) play in changing your fears that others mean you harm?	0-10	8	0.5	(−3.6 to 4.6)	TAU	7	−1.8	(−4.6 to 1.0)	iMAPS
Item 7: To which extent could you find positive / helpful ways of using the image(s)?	0-10	8	−1.0	(−4.9 to 2.9)	iMAPS	7	−1.6	(−3.5 to 0.3)	iMAPS
**Brief Core Schema Scale (BCSS)**									
Negative-self	0-24	-	-	-	-	25	2.9	(−0.8 to 6.6)	TAU
Positive-self	0-24	-	-	-	-	25	7.3	(2.8 to 11.8)	iMAPS
Negative-others	0-24	-	-	-	-	25	−2.5	(−7.0 to 2.0)	iMAPS
Positive-others	0-24	-	-	-	-	25	1.4	(−3.8 to 6.7)	iMAPS
**International Trauma Questionnaire (ITQ)**									
Post-Traumatic Stress Disorder (PTSD)	0-24	32	1.6	(−1.6 to 4.8)	iMAPS	21	0.8	(−5.9 to 7.4)	TAU
Disturbances in self- organization (DSO)	0-24	32	0.7	(−3.3 to 4.7)	iMAPS	21	−2.0	(−8.3 to 4.4)	iMAPS
Total Score	0-48	32	2.2	(−4.2 to 8.6)	iMAPS	21	−1.0	(−12.7 to 10.7)	iMAPS
**Basic Emotions Scale (BES)**									
Anger	4-28	34	0.3	(−2.2 to 2.8)	TAU	22	−3.5	(−8.0 to 1.1)	iMAPS
Sadness	4-28	34	−0.9	(−4.2 to 2.5)	iMAPS	22	−5.3	(−9.6 to −1.0)	iMAPS
Disgust	4-28	34	0.5	(−2.3 to 3.3)	TAU	22	−3.0	(−7.3 to 1.4)	iMAPS
Anxiety	4-28	34	−0.1	(−2.7 to 2.6)	iMAPS	22	−4.4	(−8.2 to −0.6)	iMAPS
Happiness	4-28	34	−0.1	(−2.8 to 2.5)	TAU	22	−0.1	(−4.6 to 4.3)	iMAPS
**Beck Anxiety Inventory (BAI)**	0-63	34	−3.6	(−10.4 to 3.2)	iMAPS	23	−5.2	(−15.0 to 4.6)	iMAPS
**Calgary Depression Scale (CDS)**	0-27	32	0.7	(−2.5 to 3.9)	TAU	21	0.8	(−3.0 to 4.5)	TAU
**Warwick Edinburgh Mental Well Being Scale (WEMWBS)**	14-70	34	−2.5	(−7.6 to 2.7)	TAU	21	−1.3	(−10.3 to 7.7)	TAU
**The Personal and Social Performance Scale (PSP)**	0-100	34	1.4	(−6.4 to 9.1)	iMAPS	20	15.5	(5.8 to 25.2)	iMAPS

*Effect estimate = iMAPS intervention + TAU—TAU*.

^*^ *Visual Analogue Scales (MIPQ/VAS) were analyzed using regression models for the most important image (*i.e., *first image). For the second and third images reported, we obtained unadjusted mean differences with bootstrap confidence intervals.*

The results of the Psychotic Symptom Rating Scales (PSYRATS) were consistent at the end of treatment (16 weeks) and follow-up (28 weeks), with the estimated effects of the auditory hallucinations subscale (PSYRATS-AH) favoring the intervention and the delusions subscale (PSYRATS-DEL) favoring TAU. Supplementary Table 4d presents an additional post-hoc analysis of the PSYRATS data for participants only experiencing both hallucinations and delusions, with a small standardised effect in favor of iMAPS on PSYRATS-AH at 16 weeks, corresponding to an unstandarised effect estimate of 3.4 (compared to 1.9 for the full sample).

Supplementary Table 5 shows the response rates as measured by the PANSS by allocated treatment and time point. Overall improvement rates were higher at 16 weeks than 28 weeks, with the proportion of participants responding being slightly higher in the intervention arm; 15/25 (60%) vs. 6/11 (54%) at 16 weeks and 8/18 (45%) vs 3/8 (39%) at 28 weeks in the intervention and TAU arms respectively. In addition, each of the up to three images participants were asked about in terms of frequency (weekly; monthly) and distress (0-100) are reported in Supplementary Tables 7–9. The results demonstrate some reduction in mean scores of distress for image 1 and image 2 between baseline and 16- and 28-week follow-ups which is also an encouraging finding.

### Adverse Events

We recorded 13 adverse events; two were rated as serious adverse events (both in iMAPS + TAU). No serious adverse events were deemed related to the trial procedures or the iMAPS therapy. The adverse reactions reported were expected (increase in distress thinking about trauma; suicidal ideation with a plan, often predating the assessment). Supplementary Table 6 details the number of adverse events and serious adverse events by treatment group and overall. We recorded suicidal ideation with a plan as *protocol defined* serious adverse events during the trial and reported them in the same way to the Sponsor to ensure a greater level of monitoring but are reported here as AEs in line with usual definitions. There were no severe symptom increases as reported on the CGI-S or the CGI-I, in either the participant or researcher measures as assessed at each contact. We also planned to record adverse effects using the adverse effects questionnaire. However, due to a Case Report Form (CRF) printing error these additional questionnaires were not administered.

### TAU Psychological Therapies Received

The review of clinical records indicated that post iMAPS therapy delivery during the follow-up stages, four iMAPS participants (4/29; 14%) accessed psychological therapies. These were CBT (2 participants), Family Interventions (1 participant) and CBT & Counselling (1 participant). In the TAU arm, five TAU participants (5/14; 35.7%) accessed psychological therapies over the trial period follow-up. These were CBT (3 participants), Family Interventions (1 participant), and Family Interventions plus CBT/DBT (1 participant).

### Therapeutic Alliance

Alliance data were available for 11/31 participants at session three, 7/31 participants at session six and 12/31 at session nine. The therapist version was available for 9/31 participants at start of therapy, 7/31 at session six and 12/31 at session nine. Alliance rated by participants was slightly higher than the ratings given by therapists and is presented in Supplementary Table 10. Alliance ratings were similar to those observed in other psychological therapy trials of psychosis.^59^ The measure could not be administered by the research assistants due to rater blinding and the therapists reported finding it challenging to arrange additional appointments with each others participants in-between sessions to administer, in addition to their core therapist duties.

### Qualitative Interview Findings—Acceptability

The qualitative feedback from (*N* = 12) participants^60^ was generally very positive. A thematic analysis study of twelve participants from the trial will be reported elsewhere but in summary, participants found that the therapy helped them make sense of their intrusive images, reduced the frequency, and helped with perceived control of their intrusive images. Participants appreciated the ease of the imagery techniques to use, being able to talk openly about their images and the importance of the therapeutic relationship. In contrast, some participants felt that sometimes the therapy could sometimes potentially elicit some negative emotions, due to its highly personal nature. However, activating negative intrusive images to work on these and improve them sometimes necessitates emotions being experienced and activated on the therapeutic journey to lasting change. Length and location of sessions seemed acceptable. More than 12 sessions would have been preferred, and participants’ felt the number of questionnaires and length of interview assessments could be reduced. A qualitative study of therapist’s experiences of delivering imagery focused therapy for psychosis^61^ (*N* = 4) also supported extending beyond 12 sessions of therapy for future studies and delivery of the therapy.

### Completion Rates for Candidate Primary Outcome Measures

The PANSS was completed at baseline by 45/45 participants, at 16 weeks by 34/36 participants and at 28 weeks by 21/26 participants. The PSYRATS was completed at baseline by 45/45 participants, at 16 weeks by 34/36 participants and at 28 weeks by 23/26 participants. The QPR was completed at baseline by 44/45, by 33/36 participants at 16 weeks and by 20/26 participants at 28 weeks. Finally, the BCSS was completed at baseline by 45/45 participants, by 30/36 participants at 16 weeks and 25/26 at 28 weeks.

### Acceptability of Candidate Primary Outcome Measures

Participants did not give specific feedback about particular questionnaires, although some did highlight in qualitative feedback that they felt the overall number of questionnaires could be reduced. Completion rates for potential candidate primary outcome measures were very good at baseline, at 16 weeks primary endpoint but reduced at 28 weeks. Further consultation with people with psychosis will be needed to identify which measure is most relevant to their experiences and what they wish to change before confirming the most appropriate primary outcome measures for a larger, powered RCT. It is also important to select an outcome relevant to the treatment target, thus new measures of negative mental imagery may also be relevant to consider.^62^ The decision on primary outcome measure will also help to determine the appropriate sample size for a future trial.

### Health Economics

Regarding the health economics feasibility questions, we found there were similar rates of completion for both the EQ-5D and ReQoL-10 for estimating cost utility. We did find that the ReQoL derived utilities were higher than the EQ-5D utilities (see Table 2). The EPQ questionnaire found that regularly used services in mental health settings, such as clinics and mental health nursing support were also offered to participants in this study as part of their usual care.

## Discussion

This is the first randomized controlled trial to evaluate the feasibility of iMAPS imagery focused therapy for people with psychosis. Overall, the study highlights the feasibility of testing an imagery focused therapy for individuals with psychosis using a randomised controlled clinical trial design and that a larger, well powered trial should be possible. The participants recruited reported a range of distressing intrusive mental images linked with their hallucinations and/or delusions and problematic negative core schematic beliefs regarding the self and others. Retention at pre-specified primary follow-up at 16 weeks was high, similar to other feasibility trials^63^ but did reduce at 28 weeks slightly below retention of other similar trials.^64^ The qualitative feedback suggests the attrition could be due to participants not wishing to undertake a large number of questionnaires at follow-up, and so reducing these could increase retention in a future trial. Extending the recruitment window also meant that the window for follow-ups was reduced and six participants were not able to be offered their 28-week assessment as planned, which could be accounted for in a future larger trial. The results confirm that the iMAPS therapy can be delivered with high levels of therapy fidelity and we can engage people with psychosis in an imagery focused psychological therapy using face to face and remote/telehealth therapy delivery. A small number of participants struggled to engage or were lost to follow up, but this is consistent with other trials in psychosis and schizophrenia.

Regarding cost-effectiveness there is no evidence to suggest either potential method to estimate utility (EQ-5D or ReQoL-UI) is more robust and completion is similar. More work is needed to validate the ReQoL-UI in similar populations prior to using it alone in a full-scale trial. The ReQoL derived utilities are higher than the EQ-5D utilities, which aligns with findings from a larger study comparing the measures in a schizophrenia population in the UK.^65^ Commonly used services (eg, clozapine clinic visits, CPN) reported in this feasibility trial will help to re-design the EPQ for use in a full trial. The overall therapy engagement adherence was high at 77%, with an average of nine sessions attended and a median of 12/12. The dropout rate is similar to the rate established in a meta analytic review examining dropout in psychological therapies for PTSD in adults.^66^ The independent therapy ratings on a sample of recordings rated on the Cognitive Therapy for Psychosis Scale (CTS-Psy) scored a mean of 48 out of a possible 60, with all tapes passing. This suggests a high quality of therapy was delivered on the trial. There is some promise of a small effect on images, positive beliefs regarding the self and positive beliefs regarding others, but less support for symptom severity and recovery from psychosis. However, the current study is a feasibility trial, with a 2:1 randomization of unequally balanced groups, and was not powered to detect differences between groups, nor was it designed to do so. There were a number of participants in the TAU control group (35.7%) who accessed psychological therapies over the trial period follow-up, which could be an argument for considering an active control or other control in a future trial. The qualitative feedback from participants^60^ was broadly very positive, highlighting how the techniques helped make sense of images, reduce frequency of intrusions, and increase control of the images. Refinements to the therapy manual include the feedback to increase the number of sessions offered to participants. A qualitative study of therapist’s experiences of delivering imagery focused therapy for psychosis^61^ also supported extending beyond 12 sessions of therapy for future studies and delivery of the therapy. The working alliance data was more challenging to collect (the research assistants were blind to allocation group and collecting it would have revealed the participant was in therapy). A future study might use an online survey tool to support it being completed a convenient times by participants. Limitations include that the trial recruited a relatively small number of participants from a minoritised ethnic group (10%), which limits generalisability. A future trial could include costs for translating materials as needed for participants who are not fluent in English and increase efforts for better representation in line publicly available local demographic information. The trial was conducted at one mental health trust in the North of England and the participants may not be representative of all individuals with psychosis and schizophrenia spectrum diagnoses accessing services. The trial design gives an indication of potential benefits of the iMAPS therapy when added to standard care but not the active ingredients of therapy which are needed or if another therapeutic approach might offer a greater treatment effect.

In summary, the findings from this RCT do support the feasibility of progressing to large multi-center randomized controlled clinical trial to evaluate the effectiveness of iMAPS therapy. iMAPS-2 was a robustly designed and delivered trial, with a pre-registered protocol, pre-registered SHEAP, and clear a priori progression criteria regarding a large multi-center RCT. Imagery focused therapy for psychosis appears safe and acceptable. An adequately powered clinical and cost effectiveness trial is warranted to provide an estimate of the effects of the iMAPS therapy.

## Supplementary Material

sbaf060_suppl_Supplementary_Tables_1-10_Figures_1

## Data Availability

On completion of the team’s publication plan, deidentified participant will be available on reasonable request in anonymised form from the corresponding author (chris.d.j.taylor@sheffield.ac.uk) with a study proposal/protocol, subject to review and a contract with Pennine Care NHS Foundation Trust. The Protocol and Statistical and Health Economic Analysis Plan (SHEAP) are available on OSF, ISRCTN and in the appendix.

## Footnotes

1 Mental Imagery “occurs when perceptual information is accessed from memory, giving rise to the experience of ‘seeing with the mind’s eye’, hearing with the mind’s ear”^67^. We define a distressing image as a potential participant reporting an mental image, a “picture in the mind’s eye,” which could also be in any one of the five senses. The images could be clear or unclear, fully formed or fleeting.

## References

[1] National Institute for Health and Care Excellence. NICE guidelines CG178 - Psychosis and schizophrenia in adults: Treatment and management. London: National Institute for Health and Care Excellence; 2014.

[2] Bighelli I, Salanti G, Huhn M, et al Psychological interventions to reduce positive symptoms in schizophrenia: systematic review and network meta-analysis. World Psychiatry 2018;17:316–329. 30192101 10.1002/wps.20577 PMC6127754

[3] Schulze K, Freeman D, Green C, Kuipers E. Intrusive mental imagery in patients with persecutory delusions. Behav Res Ther. 2013;51:7–14. 23178174 10.1016/j.brat.2012.10.002

[4] Morrison A, Beck A, Glentworth D, et al Imagery and psychotic symptoms: A preliminary investigation. Behav Res Ther. 2002;40:1053–1062. 12296490 10.1016/s0005-7967(01)00128-0

[5] Taylor CDJ, Harper SF. Early maladaptive schema, social functioning and distress in psychosis: A preliminary investigation. Clinical Psychologist 2017;21:135–142.

[6] Kingdon DG, Turkington D. Cognitive-behavioral therapy of schizophrenia: 2nd Edition. London: Guilford Press; 2005.

[7] Morrison A, Renton J, Dunn H, Williams S, Bentall R, eds. Cognitive therapy for psychosis: A formulation-based approach: Routledge; 2004.

[8] Fowler D, Garety P, Kuipers E. Cognitive behaviour therapy for psychosis: Theory and practice. Chichester: Wiley 1995.

[9] Alameda L, Christy A, Rodriguez V, et al Association Between Specific Childhood Adversities and Symptom Dimensions in People With Psychosis: Systematic Review and Meta-Analysis. Schizophr Bull. 2021;47:975–985. 33836526 10.1093/schbul/sbaa199 PMC8266673

[10] Taylor CDJ, Haddock G, Speer S, Bee PE. Characterizing core beliefs in psychosis: a qualitative study. Behavioural and Cognitive Psychotherapy 2020;48:67–81. 30957739 10.1017/S1352465819000274 PMC7039701

[11] Haddock G, Berry K, Davies G, et al Delivery of cognitive-behaviour therapy for psychosis: a service user preference trial. Journal of Mental Health 2018;27:336–344. 29271276 10.1080/09638237.2017.1417549

[12] Cairns AJJ, Taylor CDJ, Kelly JA. The outcomes of imagery-focused interventions in relation to distress in people with delusions: a systematic literature review. Behavioural and Cognitive Psychotherapy 2024;52:596–615. 39308236 10.1017/S1352465824000237

[13] Ison R, Medoro L, Keen N, Kuipers E. The Use of Rescripting Imagery for People with Psychosis Who Hear Voices. Behavioural and Cognitive Psychotherapy 2014;42:129–142. 23920004 10.1017/S135246581300057X

[14] Paulik G, Steel C, Arntz A. Imagery rescripting for the treatment of trauma in voice hearers: a case series. Behavioural and Cognitive Psychotherapy 2019;47:709–725. 30975230 10.1017/S1352465819000237

[15] Sheaves B, Onwumere J, Keen N, Kuipers E. Treating your worst nightmare: a case-series of imagery rehearsal therapy for nightmares in individuals experiencing psychotic symptoms. The Cognitive Behaviour Therapist 2015;8:e27.

[16] Keen N, Hunter ECM, Peters E. Integrated Trauma-Focused Cognitive-Behavioural Therapy for Post-traumatic Stress and Psychotic Symptoms: A Case-Series Study Using Imaginal Reprocessing Strategies. Front Psychiatry. 2017;8:92. 28620323 10.3389/fpsyt.2017.00092 PMC5451497

[17] Lincoln TM, Hohenhaus F, Hartmann M. Can Paranoid Thoughts be Reduced by Targeting Negative Emotions and Self-Esteem? An Experimental Investigation of a Brief Compassion-Focused Intervention. Cognit Ther Res. 2013;37:390–402.

[18] Taylor CDJ, Bee PE, Kelly J, Haddock G. iMAgery focused therapy for persecutory delusions in PSychosis (iMAPS): A novel treatment approach. Cognitive and Behavioural Practice 2019;25:575–588.

[19] Taylor C, Bee PE, Kelly J, Emsley R, Haddock G. iMAgery focused psychological therapy for persecutory delusions in PSychosis (iMAPS): a multiple baseline experimental case series. Behavioural and Cognitive Psychotherapy 2020;48:530–545. 32264985 10.1017/S1352465820000168

[20] Cairns AJJ, Kelly J, Taylor CDJ. Assessing the delivering of iMAgery-focused therapy for PSychosis (iMAPS) via telehealth. Psychol Psychother . 2023;96:678–696. 37002818 10.1111/papt.12463

[21] Taylor et al iMAgery focused therapy for PSychosis (iMAPS-2): Protocol for a feasiblity randomised controlled clinical trial. Available at: https://osf.io/pvaz3. Accessed 11th May 2022.

[22] Kay SR, Fiszbein A, Opler LA. The Positive and Negative Syndrome Scale (PANSS) for Schizophrenia. Schizophr Bull. 1987;13:261–276. 3616518 10.1093/schbul/13.2.261

[23] Hackmann A, Surawy C, Clark DM. Seeing yourself through other’s eyes: A study of spontaneously occuring images in social phobia. Behavioural and Cognitive Psychotherapy 1998;26:3–12.

[24] Yung AR, Yung AR, Pan Yuen H, et al Mapping the onset of psychosis: the comprehensive assessment of at-risk mental states. Aust N Z Psychiatry. 2005;39:964–971. 10.1080/j.1440-1614.2005.01714.x 16343296

[25] Sim J, Lewis M. The size of a pilot study for a clinical trial should be calculated in relation to considerations of precision and efficiency. J Clin Epidemiol 2012;65:301–308. 22169081 10.1016/j.jclinepi.2011.07.011

[26] Haddock G, Devane S, Bradshaw T, et al An investigation into the psychometric properties of the Cognitive Therapy Scale for Psychosis (CTS-Psy). Behavioural and Cognitive Psychotherapy 2001;29:221–233.

[27] Avery KNL, Williamson PR, Gamble C, et al; members of the Internal Pilot Trials Workshop supported by the Hubs for Trials Methodology Research. Informing efficient randomised controlled trials: exploration of challenges in developing progression criteria for internal pilot studies. BMJ Open 2017;7:e013537. 10.1136/bmjopen-2016-013537 PMC5318608 28213598

[28] Jolley S, Garety P, Peters E, et al Opportunities and challenges in Improving Access to Psychological Therapies for people with Severe Mental Illness (IAPT-SMI): evaluating the first operational year of the South London and Maudsley (SLaM) demonstration site for psychosis. Behav Res Ther. 2015;64:24–30. 25499927 10.1016/j.brat.2014.11.006

[29] Holmes EA, Bonsall MB, Hales SA, et al Applications of time-series analysis to mood fluctuations in bipolar disorder to promote treatment innovation: a case series. Transl Psychiatry. 2016;6:e720. 26812041 10.1038/tp.2015.207 PMC5068881

[30] Fowler D, Freeman D, Smith B, et al The Brief Core Schema Scales (BCSS): psychometric properties and associations with paranoia and grandiosity in non-clinical and psychosis samples. Psychol Med. 2006;36:749–759. 16563204 10.1017/S0033291706007355

[31] Shafer A, Dazzi F. Meta-analysis of the positive and Negative Syndrome Scale (PANSS) factor structure. J Psychiatr Res. 2019;115:113–120. 31128501 10.1016/j.jpsychires.2019.05.008

[32] Haddock G, McCarron J, Tarrier N, Faragher EB. Scales to measure dimensions of hallucinations and delusions: the psychotic symptom rating scales (PSYRATS). Psychol Med. 1999;29:879–889. 10473315 10.1017/s0033291799008661

[33] Law H, Neil ST, Dunn G, Morrison AP. Psychometric properties of the Questionnaire about the Process of Recovery (QPR). Schizophr Res. 2014;156:184–189. 24816049 10.1016/j.schres.2014.04.011

[34] Pitt L, Kilbride M, Nothard S, Welford M, Morrison AP. Researching recovery from psychosis: a user-led project. Psychiatric Bulletin 2007;31:55–60.

[35] Carr S, Hardy A, Fornells-Ambrojo M. The Trauma and Life Events (TALE) checklist: development of a tool for improving routine screening in people with psychosis. European journal of psychotraumatology 2018;9:1512265. 30220986 10.1080/20008198.2018.1512265 PMC6136359

[36] Airey ND, Taylor CDJ, Vikram A, Berry K. Trauma measures for use with psychosis populations: A systematic review of psychometric properties using COSMIN. Psychiatry Res. 2023;323:115163. 36948019 10.1016/j.psychres.2023.115163

[37] Cloitre M, Shevlin M, Brewin CR, et al The International Trauma Questionnaire: Development of a self‐report measure of ICD‐11 PTSD and complex PTSD. Acta Psychiatr Scand. 2018;138:536–546. 30178492 10.1111/acps.12956

[38] Power MJ. The structure of emotion: An empirical comparison of six models. Cognition & Emotion 2006;20:694–713.

[39] Beck AT, Epstein N, Brown G, Steer RA. An inventory for measuring clinical anxiety: Psychometric properties. J Consult Clin Psychol. 1988;56:893–897. 3204199 10.1037//0022-006x.56.6.893

[40] Addington D, Addington J, Schissel B. A depression rating scale for schizophrenics. Schizophr Res. 1990;3:247–251. 2278986 10.1016/0920-9964(90)90005-r

[41] Tennant R, Hiller L, Fishwick R, et al The Warwick-Edinburgh Mental Well-being Scale (WEMWBS): development and UK validation. Health and Quality of Life Outcomes 2007;5:63. 18042300 10.1186/1477-7525-5-63 PMC2222612

[42] Morosini PL, Magliano L, Brambilla L, Ugolini S, Pioli R. Development, reliability and acceptability of a new version of the DSM-IV Social and Occupational Functioning Assessment Scale (SOFAS) to assess routine social functioning. Acta Psychiatr Scand. 2000;101:323–329. 10782554

[43] Janssen MF, Pickard AS, Golicki D, et al Measurement properties of the EQ-5D-5L compared to the EQ-5D-3L across eight patient groups: a multi-country study. Quality of life research : an international journal of quality of life aspects of treatment, care and rehabilitation 2013;22:1717–1727. 23184421 10.1007/s11136-012-0322-4 PMC3764313

[44] Keetharuth AD, Brazier J, Connell J, et al Recovering Quality of Life (ReQoL): a new generic self-reported outcome measure for use with people experiencing mental health difficulties. The British journal of psychiatry : the journal of mental science 2018;212:42–49. 29433611 10.1192/bjp.2017.10 PMC6457165

[45] Davies LM, Lewis S, Jones PB, et al; CUtLASS team. Cost-effectiveness of first- v. second-generation antipsychotic drugs: results from a randomised controlled trial in schizophrenia responding poorly to previous therapy. The British journal of psychiatry : the journal of mental science 2007;191:14–22. 17602120 10.1192/bjp.bp.106.028654

[46] Hatcher RL, Gillaspy JA. Development and validation of a revised short version of the working alliance inventory. Psychother Res. 2006;16:12–25.

[47] Hatcher RL, Lindqvist K, Falkenström F. Psychometric evaluation of the Working Alliance Inventory-Therapist version: Current and new short forms. Psychother Res. 2020;30:706–717. 31621525 10.1080/10503307.2019.1677964

[48] Bordin ES. The generalizability of the psychoanalytic concept of the working alliance. Psychotherapy: Theory, Research & Practice 1979;16:252–260.

[49] Klingberg S, Herrlich J, Wiedemann G, et al Adverse effects of cognitive behavioral therapy and cognitive remediation in schizophrenia: results of the Treatment of Negative Symptoms Study. J Nerv Ment Dis. 2012;200:569–576. 22759932 10.1097/NMD.0b013e31825bfa1d

[50] Health_Research_Authority. UK Policy Framework for Health and Social Care Research. Available at: https://www.hra.nhs.uk/planning-and-improving-research/policies-standards-legislation/uk-policy-framework-health-social-care-research/uk-policy-framework-health-and-social-care-research/

[51] International_Council_for_Harmonisation. ICH Guidelines for Good Clinical Practice E6 (R2) Scientific Guideline. Available at: https://www.ema.europa.eu/en/ich-e6-r2-good-clinical-practice-scientific-guideline.

[52] Klingberg S, Wittorf A, Meisner C, et al Cognitive behavioural therapy versus supportive therapy for persistent positive symptoms in psychotic disorders: The POSITIVE Study, a multicenter, prospective, single-blind, randomised controlled clinical trial. Trials 2010;11:123. 21190574 10.1186/1745-6215-11-123 PMC3022781

[53] Guy W. ECDEU assessent manual for psychopharmacology. In: US Department of HEalth EaW, Public Health Service, ed; 1976.

[54] Hermes E, Sokoloff D, Stroup TS, Rosenheck RA. Minimum clinically important difference in the Positive and Negative Syndrome Scale with data from the Clinical Antipsychotic Trials of Intervention Effectiveness (CATIE). J Clin Psychiatry. 2012;73:526–532. 22579152 10.4088/JCP.11m07162 PMC3786588

[55] Hutton P, Kelly J, Taylor CD, et al Accelerating the development of a psychological intervention to restore treatment decision-making capacity in patients with schizophrenia-spectrum disorder: a study protocol for a multi-site, assessor-blinded, pilot Umbrella trial (the DEC: IDES trial). Pilot and Feasibility Studies 2023;9:1–17. 37422659 10.1186/s40814-023-01323-0 PMC10329297

[56] Braun V, Clarke V. Reflecting on reflexive thematic analysis. Qualitative Research in Sport, Exercise and Health 2019;11:589–597.

[57] National_Institute_for_Health_&_Care_Excellence. NICE health technology evaluations: the manual Process and methods-and-conditions#notice-of-rights). (PMG36). Available at: https://www.nice.org.uk/process/pmg36

[58] Keetharuth AD, Rowen D, Bjorner JB, Brazier J. Estimating a Preference-Based Index for Mental Health From the Recovering Quality of Life Measure: Valuation of Recovering Quality of Life Utility Index. Value in health : the journal of the International Society for Pharmacoeconomics and Outcomes Research 2021;24:281–290. 33518035 10.1016/j.jval.2020.10.012 PMC7871010

[59] White R, Gumley A, McTaggart J, et al A feasibility study of Acceptance and Commitment Therapy for emotional dysfunction following psychosis. Behav Res Ther. 2011;49:901–907. 21975193 10.1016/j.brat.2011.09.003

[60] Dyer K, Berry K, Boland A, et al iMAgery focused therapy for psychosis (iMAPS-2): Exploring service-users’ perceptions and experiences of a novel therapy for psychosis. Manuscript submitted for Publication 2025.

[61] Guillemard S , et al Therapist Experiences of Delivering Imagery Focused Therapy for Psychosis. Manuscript submitted for Publication 2025.

[62] Oaie FE, Bower JL, Steel C. The development of the Negative Mental Imagery Questionnaire (MIQ-N). Behavioural and Cognitive Psychotherapy 2024;53:17–29. 39421888 10.1017/S1352465824000304

[63] Morrison AP, Law H, Carter L, et al Antipsychotic drugs versus cognitive behavioural therapy versus a combination of both in people with psychosis: a randomised controlled pilot and feasibility study. The Lancet Psychiatry 2018;5:411–423. 29605187 10.1016/S2215-0366(18)30096-8 PMC6048761

[64] Steel C, Hardy A, Smith B, et al Cognitive–behaviour therapy for post-traumatic stress in schizophrenia. A randomized controlled trial. Psychol Med. 2017;47:43–51. 27650432 10.1017/S0033291716002117

[65] Franklin M, Enrique A, Palacios J, Richards D. Psychometric assessment of EQ-5D-5L and ReQoL measures in patients with anxiety and depression: construct validity and responsiveness. Qual Life Res. 2021;30:2633–2647. 33835414 10.1007/s11136-021-02833-1 PMC8034045

[66] Lewis C, Roberts NP, Gibson S, Bisson JI. Dropout from psychological therapies for post-traumatic stress disorder (PTSD) in adults: systematic review and meta-analysis. Eur J Psychotraumatol 2020;11:1709709. 32284816 10.1080/20008198.2019.1709709 PMC7144189

[67] Kosslyn SM, Ganis G, Thompson WL. Neural foundations of imagery. Nat Rev Neurosci. 2001;2:635–642. 11533731 10.1038/35090055

