# Supplementary material for: iMAgery Focused Therapy for PSychosis (iMAPS-2): An Assessor-blind Feasibility Randomized Controlled Clinical Trial
Source: Schizophr Bull. 2025 Oct 6;51(Suppl 3):S317–35. doi: 10.1093/schbul/sbaf060 (PMC12498916; doi:10.1093/schbul/sbaf060)
Supplement: sbaf060_suppl_Supplementary_Tables_1-10_Figures_1 [file sbaf060_suppl_supplementary_tables_1-10_figures_1.docx]

**Supplementary Online Content**

Christopher D. J. Taylor, Ben Helliwell, Rebecca Coleman, Chris Sutton, Paul Hutton, Yvonne Sylvestre, Leanne Bird, Gemma Shields, James A. Kelly, Thomas Brandwood-Spencer, Alicia Boland, Emma Sharrock-Ingham, Bukunmi Babatunde, Amy Beech, Anvita Vikram, Serena Gulliemard, Arnoud Arntz, Sean F. Harper and Katherine L. Berry**.**

*iMAgery focused therapy for PSychosis (iMAPS-2): an assessor-blind feasibility randomised controlled clinical trial*

**Contents**

| Protocol – imagery focused therapy for Psychosis: Protocol for a feasibility randomised controlled clinical trial Version 6.0 | p.2 |
| --- | --- |
| iMAPS-2 Statistical and Health Economics Analysis Plan (SHEAP) | p.52 |
| Supplementary Table 1: Monthly and cumulative randomisation | p.82 |
| Supplementary Figure 1: Distribution of the number of therapy sessions attended and their duration in minutes | p.83 |
| Supplementary Table 2: Baseline trauma and life events (TALE) by treatment allocated | p.84 |
| Supplementary Table 3: Baseline clinical measures by treatment allocated | p.85 |
| Supplementary Table 4a: Post Hoc Analysis - Frequency (%) of participants by time-point with presence of both auditory hallucinations & delusions, hallucinations or delusions only at baseline | p.88 |
| Supplementary Table 4b: Post Hoc Analysis Baseline summary measures of the Psychotic Symptom Rating Scales (PSYRATS) for those participants with auditory hallucinations & delusions at baseline | p.88 |
| Supplementary Table 4c: Post Hoc Analysis Follow up summary measures of the Psychotic Symptom Rating Scales (PSYRATS) for those participants with auditory hallucinations & delusions at baseline | p.89 |
| Supplementary Table 4d: Post Hoc Analysis Treatment effects at 16 weeks of the Psychotic Symptom Rating Scales (PSYRATS) for those participants with auditory hallucinations & delusions at baseline | p.89 |
| Supplementary Table 5: Response rates as measured by the Positive and Negative Syndrome Scale (PANSS) by treatment allocated and timepoint | p.91 |
| Supplementary Table 6: Number of Adverse Events and Serious Adverse Events by Treatment Group and Overall | p.92 |
| Supplementary Table 7a: Image 1 How often have you experienced the image in the past week? | p.93 |
| Supplementary Table 7b: Image 1 How often have you experienced the image in the past month? | p.93 |
| Supplementary Table 7c: Image 1 How distressing was the image at the time (0-100)? | p.93 |
| Supplementary Table 8a: Image 2 How often have you experienced the image in the past week? | p.94 |
| Supplementary Table 8b: Image 2 How often have you experienced the image in the past month? | p.94 |
| Supplementary Table 8c: Image 2 How distressing was the image at the time (0-100)? | p.94 |
| Supplementary Table 9a: Image 3 How often have you experienced the image in the past week? | p.95 |
| Supplementary Table 9b: Image 3 How often have you experienced the image in the past month? | p.95 |
| Supplementary Table 9c: Image 3 How distressing was the image at the time (0-100)? | p.95 |
| Supplementary Table 10: Working Alliance Inventory (WAI) Scores – Participant and Therapist Versions | p.96 |
| Supplementary Table 11: Combined CONSORT reporting checklist (Pilot; Social and Psychological Interventions) | p.97 |
| Supplementary Table 12: More information on timing and purpose of changes and Amendments | p.106 |


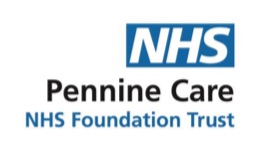


Study Protocol

# Administrative Information

Full Title: iMAgery focused therapy for Psychosis (iMAPS-2): A feasibility randomised controlled trial

Protocol Version: 6.0

Protocol Date: 30^th^ June 2024

Funded by: National Institute for Health Research (NIHR)

Sponsor: Pennine Care NHS Foundation Trust

Ethics References: 22/YH/0091 IRAS Project ID: 309409

Chief Investigator: Dr Christopher Taylor (Pennine Care NHS Foundation Trust & The University of Manchester)

Investigators: Professor Katherine Berry (The University of Manchester), Dr Chris Sutton (The University of Manchester), Dr James Kelly (Lancaster University & Greater Manchester Mental Health NHS Foundation Trust), Professor Paul Hutton (Edinburgh Napier University), Yvonne Sylvestre (University of Manchester), Dr Sean Harper (NHS Lothian), Thomas Brandwood-Spencer (Pennine Care NHS Foundation Trust), Gemma Shields (The University of Manchester)

Collaborator: Professor Arnoud Arntz (University of Amsterdam)

# SIGNATURE PAGE

The undersigned confirm that the following protocol has been agreed and accepted and that the Chief Investigator agrees to conduct the trial in compliance with the approved protocol and will adhere to the principles of GCP, the Sponsor’s SOPs, and other regulatory requirements as amended.

I agree to ensure that the confidential information contained in this document will not be used for any other purpose other than the evaluation or conduct of the clinical investigation without the prior written consent of the Sponsor.

I also confirm that I will make the findings of the study publicly available through publication or other dissemination tools without any unnecessary delay and that an honest accurate and transparent account of the study will be given; and that any discrepancies from the study as planned in this protocol will be explained.

| **Study Sponsor:** | | |
| --- | --- | --- |
| Signature:    ...................................................................................................... |  | Date: ....../........../......  ddmmmyyyy |
| Name (please print): Simon Kaye  ...................................................................................................... |  |  |
| Position: Research and Innovation Manager.................................................................................... |  |  |
| **Chief Investigator:** | | |
| Signature: ...................................................................................................... |  | Date: ....../........../......  ddmmmyyyy |
| Name: (please print): Dr Chris Taylor  ...................................................................................................... |  |  |
| **Statistician:** |  |  |
| Signature: ...................................................................................................... |  | Date:  ....../........../......  ddmmmyyyy |
| Name: (please print): Yvonne Sylvestre & Dr Chris Sutton  ...................................................................................................... |  |  |
| Position: Research Fellow in Clinical Trials Statistics.  Senior Lecturer in Clinical Trials |  |  |

**SIGNATURE PAGE**

**Principal Investigator**

As Principal Investigator for the iMAPS-2 trial I confirm that I will be responsible to ensure that all members of the local clinical trial team are appropriately trained on the trial protocol and have the relevant qualifications and experience to carry out their role in accordance with the trial protocol.

| Name | Site | Signature | Date  dd/mmm/yyyy |
| --- | --- | --- | --- |
| Dr Chris Taylor | Pennine Care NHS FT |  |  |

**Study Identifiers**

| ISRCTN: | 81150786 | | |
| --- | --- | --- | --- |
| REC Reference: | 22/YH/009 | | |
| UKCRN Number: | N/A | | |
| Protocol Version: | 6.0 | Date:  30th June 2024 |  |
| Start Date: | HRA Favourable Ethical Opinion: 6^th^ May 2022  Pennine Care NHS FT Approval date: 14^th^ June 2022 | | |
| End Date: | 30^th^ June 2025 |  |  |

**Sponsor**

| **Sponsor Contacts:** | Simon Kaye, Research & Innovation Lead |
| --- | --- |
| **Address:** | Pennine Care NHS Foundation Trust  Research and Innovation Department  Trust Headquarters  225 Old Street  Ashton-under-Lyne  OL6 7SR |
| **Telephone:** | Phone: 0161 716 3892/3993  Simon Kaye Mobile: 0777 500 7114 |
| **Email:** | [simon.kaye2@nhs.net](mailto:simon.kaye2@nhs.net) |

**Chief Investigator**

| **Name:** | Dr Christopher Taylor, Consultant Clinical Psychologist |
| --- | --- |
| **Role:** | Chief Investigator |
| **Address:** | Community Mental Health Team,  Humphrey House, Angouleme Way, Bury, BL9 0EQ |
| **Phone:** | 0161 253 7667 |
| **Email:** | chrisdjtaylor@nhs.net |

Version History

| **Document ID** | **Description of changes from previous version** | | **Effective Date** |
| --- | --- | --- | --- |
| Protocol V 1.0 | First Protocol submitted for HRA Review |  | |
| Protocol V 2.0 | Revised Version submitted to achieve HRA Favourable Ethical Opinion | 21/04/2022 | |
| Protocol V 3.0 | 1. Reduce Inclusion Criteria Age Range from 18 years to 16 years old, to expand access to a greater number of Early Intervention Psychosis service users/patients.  2. Expand recruitment to include adult inpatient wards, for patients who are judged to have capacity to consent as assessed by a key clinician, to expand access to the study for potentially eligible service users/patients.  3. To capture imagery characteristics each therapy session, as a potential mechanism of change  4. Seek verbal consent to document demographics at screening stage, to record any differences between those screened and those eventually randomised, to support maximising purposive sampling of participants from different ethnic minority backgrounds. | 21/11/2022 Amendment approved by HRA | |
| Protocol V 4.0 | 1. Substantial Amendment requested to add a third qualitative study. The aim of the study is explore therapist's experiences of delivering imagery focused therapy through interviews and to amend participant’s qualitative interview schedule. |  | |
| Protocol V 5.0 | 1. Non Substantial Amendment to extend study end date to 30^th^ June 2024, to allow 16 week and 28 week assessments to be completed. |  | |
| Protocol V 6.0 | Non Substantial Amendment to extend study for final analysis, checking and staff interview qualitative study to be completed on a no cost basis until 30^th^ June 2025 . | 30^th^ June 2024 | |

|  |  |  |
| --- | --- | --- |

CONTENTS

[1 Administrative Information 1](#_Toc170740665)

[SIGNATURE PAGE 2](#_Toc170740666)

[2 INTRODUCTION 9](#_Toc170740667)

[2.1 BACKGROUND & RATIONALE FOR STUDY 9](#_Toc170740668)

[2.2 OBJECTIVES AND RESEARCH QUESTIONS 10](#_Toc170740669)

[2.3 Trial Design 10](#_Toc170740670)

[3 METHODS 11](#_Toc170740671)

[3.1 Study Setting 11](#_Toc170740672)

[3.2 Eligibility CRITERIA 11](#_Toc170740673)

[3.3 Trial procedures 12](#_Toc170740674)

[3.4 Recruitment 14](#_Toc170740675)

[3.5 Patient Identification 15](#_Toc170740676)

[3.6 Consent And eligibility 15](#_Toc170740677)

[3.6.1 Participant withdrawal 17](#_Toc170740678)

[3.7 Assignments of Interventions 17](#_Toc170740679)

[3.8 Allocation 17](#_Toc170740680)

[3.9 (Allocation Implementation) 18](#_Toc170740681)

[3.10 Blinding 18](#_Toc170740682)

[3.11 Outcome Measures (Feasibility, Clinical and Health Cost) 19](#_Toc170740683)

[3.11.1 Number of Assessments and Measures (Participant Burden) 24](#_Toc170740684)

[3.12 FOLLOW UP ASSESSMENTS 24](#_Toc170740685)

[3.13 Withdrawal 24](#_Toc170740686)

[3.14 Lost to Follow Up 25](#_Toc170740687)

[3.15 End of the Trial 25](#_Toc170740688)

[3.16 INTERVENTION 25](#_Toc170740689)

[3.17 ADVERSE EVENTS 27](#_Toc170740690)

[3.18 Operational definitions for (S)AEs 29](#_Toc170740691)

[3.19 Reporting urgent safety measures 30](#_Toc170740692)

[3.19.1 Risks associated with non-clinical (research) procedures 30](#_Toc170740693)

[3.19.2 Risks associated with therapy procedures 32](#_Toc170740694)

[3.20 RESEARCHERS and CLINICIANS 32](#_Toc170740695)

[3.20.1 Risk of violence from participants 32](#_Toc170740696)

[3.20.2 Vicarious trauma 33](#_Toc170740697)

[3.21 Sample Size 33](#_Toc170740698)

[3.22 ANALYSIS METHODS 33](#_Toc170740699)

[3.23 DATA HANDLING 35](#_Toc170740700)

[3.24 CRFs as Source Documents 35](#_Toc170740701)

[3.25 DATA Monitoring 36](#_Toc170740702)

[3.26 Data Collection, Source Data and Confidentiality 36](#_Toc170740703)

[3.26.1 Study Site Staff and Training 39](#_Toc170740704)

[3.27 Data Monitoring and Quality Assurance 39](#_Toc170740705)

[3.28 Data Handling and Recording Keeping 39](#_Toc170740706)

[3.29 Archiving 40](#_Toc170740707)

[3.30 MONITORING 40](#_Toc170740708)

[3.31 Trial Steering Committee (TSC) 40](#_Toc170740709)

[4 ETHICS and DISSEMINATION 41](#_Toc170740710)

[4.1 RESEARCH ETHICS COMMITTEE REVIEW AND REPORTS 41](#_Toc170740711)

[4.2 PROTOCOL AMENDMENTS 41](#_Toc170740712)

[4.3 PEER REVIEW 41](#_Toc170740713)

[4.4 Public Patient Involvement 41](#_Toc170740714)

[4.5 Protocol Compliance 41](#_Toc170740715)

[4.6 Notification of Serious Breaches to GCP and/ or the protocol 42](#_Toc170740716)

[4.7 SERIOUS BREACH OF PROTOCOL 42](#_Toc170740717)

[4.8 INSPECTION OF RECORDS 42](#_Toc170740718)

[4.8.1 Data Protection 42](#_Toc170740719)

[4.9 INSURANCE AND INDEMNITY 43](#_Toc170740720)

[4.10 DISSEMINATION POLICY 43](#_Toc170740721)

[AUTHORSHIP POLICY 43](#_Toc170740722)

[4.10.1 Study suspension or discontinuation 44](#_Toc170740723)

[5 REFERENCES 46](#_Toc170740724)

[APPENDICES: 50](#_Toc170740725)

[APPENDIX 1: Proposed CONSORT Flow Diagram 50](#_Toc170740726)

**Scientific Summary**

**Aims**

Our research aims to improve the current treatments for people with psychosis and schizophrenia spectrum diagnoses. Our main aim is to assess whether it is feasible to conduct a randomised trial to examine the (clinical and cost) effectiveness of an imagery focused psychological therapy in psychosis. The therapy specifically targets distressing “mental imagery” e.g. *“pictures in the mind’s eye, sounds in the mind’s ear”*, and negative beliefs, frequently reported but infrequently treated.

**Background**

Schizophrenia is a severe mental health condition, for example,

where individuals hear distressing voices other people cannot hear (e.g. auditory hallucinations) and/or have distressing unusual beliefs that others do not share (delusions). They also report negative core beliefs (e.g. “I am vulnerable; I am a failure; Others are hostile”) and unwanted intrusive mental images (that are in the “mind’s eye” and other senses) which may be maintaining factors for psychotic symptoms. One of the best interventions for psychosis is Cognitive Behavioural Therapy (CBT), which is recommended by the UK National Institute for Health and Care Excellence (NICE). Sadly, the first generation of CBT for Psychosis (adapted from CBT for emotional disorders) has small effect size, and there is a need to refine and improve it. Imagery approaches are almost completely absent from multiple CBT for Psychosis therapy manuals. Empirical studies consistently demonstrate that imagery has a more powerful impact on emotion than verbal cognition. Therefore, we anticipate using an imagery focused approach to target images and schemas will result in a reduction in psychotic symptoms.

We wish to undertake a feasibility randomised controlled trial of an imagery focused therapy called iMAPS, which targets negative images and negative core beliefs (schemas). The project will tell us if we may be able to run a definitive randomised controlled clinical trial.

**Design and methods to be used**

We will ask 45 patients with psychosis to take part in our study with a 2:1 randomisation ratio: 30 will be offered iMAPS therapy and their usual care and the other 15 patients will continue with their usual care only. The research assistants will be blind to the group allocation.

**Patient and Public Involvement (PPI)**

Patients have been involved throughout: in helping to design the first studies and the therapy. The project also has a dedicated PPI co-applicant who will provide a patient perspective and input into the design and management of the project, as well as a service user reference group (SURG) who will meet every few months to have input into the project and shape the direction. The role will also involve deciding which measures are useful, reviewing documentation, they will assist with interpretation of results and also support dissemination activities.

**Lay Summary**

“*I really do think the therapy helped me. The images don’t really occur too much anymore. I don’t think about them…, as much as I did before the therapy. I’m putting “changing the meaning of memories [imagery therapy]”, I’m using that for other things that have troubled me in the past.” Participant* iMAPS-1

**Aims**

Our research aims to improve the current treatments for people with psychosis. We wish to explore a psychological therapy where the therapist and client specifically work with distressing “mental imagery” e.g. *“pictures in the mind’s eye, sounds in the mind’s ear”*, and negative beliefs, which are often reported but rarely treated.

**Background**

Psychosis is a mental health condition, where individuals hear distressing voices other people cannot hear and/or have distressing unusual beliefs, which others do not share. They also report negative core beliefs called schema (e.g. “I am a failure; others are hostile”) and unwanted (intrusive) pictures in the mind’s eye “mental images”. Sadly, current psychological therapies do not work for everyone.

We have tested a more specific way of working with negative beliefs and images in psychosis which targets and uses imagery.

**Methods**

We will ask 45 people with psychosis to take part. 30 will receive iMAPS therapy and usual care and the other 15 their usual care only. This will be decided by chance (randomly). For every two people who are allocated to therapy, one person will be allocated to usual care alone in the control group (a 2:1 randomisation rate). The project will tell us if we may be able to run a bigger version of the trial. We will only know if the intervention works, when we do the bigger study, but this is an important step.

**Patient and Public Involvement**

Patients have been and will be involved in: helping to design study and the therapy, deciding on measures, helping us highlight key findings from a service user perspective the study, interpreting the findings and helping us spread the word.

We will tell people about what we find including via practice manuals, workshops and conferences and scientific publications. We will also hold events online and in person to inform academics, clinicians, commissioners, service users, carers and the wider public about our findings.

# INTRODUCTION

## BACKGROUND & RATIONALE FOR STUDY

*“John has spent over half his adult life grappling with uncontrollable intrusive images of him and his family being killed by a secret organisation and fears this will happen unexpectedly; sending him spiralling into worry, fear and despair. Adverse experiences in early life led to John developing negative beliefs about himself and others, which worsen his paranoid fears. At 29, John tried to kill himself, hoping to escape his persecutors.”*

Schizophrenia, a frequently diagnosed form of psychosis, is a significant challenge for the National Health Service (NHS), with substantial human suffering, disability and financial costs (e.g. £12.5 Billion in England (National_Institute_for_Health_and_Care_Excellence, 2014)). Service users diagnosed with schizophrenia experience psychotic symptoms such as hallucinations (e.g., hearing voices that other people cannot) and delusions (e.g., paranoia and strongly held distressing unusual beliefs such as *“These figures I see intend to cause me harm and kill me.”)* A number of people with psychosis respond to initial treatment with antipsychotic medication but around 80% relapse within five years of first episode. Suicide risk is sadly six to 14 times more likely in people with psychosis compared to individuals in the general population (Tanskanen et al., 2018; Westman et al., 2018).

Cognitive Behavioural Therapy (CBT), one of the current NICE recommended psychological therapies, has demonstrated a small effect on hallucinations and delusions (National_Institute_for_Health_and_Care_Excellence, 2014). However, it does not work for everyone (Jauhar et al., 2014). One of the reasons for poor efficacy could be that standard CBT does not directly tackle distressing imagery, one of the key aspects experienced by many people with psychosis, often related to trauma and/or their psychotic experiences. Adverse life experiences (e.g. trauma) are frequently reported by people with psychosis (Varese et al., 2012) and can lead to individuals developing strongly held negative beliefs about self and others (schema), often with associated intrusive mental images (e.g. flashbacks, pictures in your head (Schulze et al., 2013). Despite up to 74% of people with psychosis experiencing distressing intrusive mental images (Morrison et al., 2002; Schulze et al., 2013) and high levels of negative schemas - beliefs about the self and others (Taylor & Harper, 2017), there are few references to imagery work in existing CBT for Psychosis manuals e.g. (Fowler et al., 1995; Kingdon & Turkington, 2005; Morrison et al., 2004).

Taylor et al. (2020) used a qualitative approach to explore core beliefs and schema in psychosis and their links with hallucinations and paranoia. Four emergent themes were identified including links between beliefs and images. Our study examining schemas in psychosis using a daily diary method demonstrated that strongly held negative beliefs about the self and others explained a significant proportion of the severity of voices, delusions and how distressed people were by these experiences (Taylor et al., 2022; Under Review). The existing core belief (schema) techniques from standard CBT for depression or anxiety are frequently under-utilised by therapists working with psychosis; in the largest trial of CBT for Psychosis anywhere in the world (Morrison et al., 2018; 3,056 sessions) only 8% of sessions focusing on change techniques used schema change (Bowe, 2017).

In recent years, there have been a small number of studies making use of imagery in psychosis in individual single case studies or small case series.

Ison et al. (2007; 2014) conducted a series of small case series using imagery re-scripting alone to work with voices. There was subsequently a larger case series focusing on imagery rescripting for voice hearers who have experienced trauma (Paulik et al., 2019), nightmares (Sheaves et al., 2015) and imaginal reprocessing of traumatic experiences (Keen et al., 2017) which have led to reductions in distress, conviction in beliefs associated with images and emotions. Imagery approaches can also include the use of positive imagery techniques, which are also often missing from existing treatment manuals.

IMAPS was also developed working with people who experience psychosis. The therapy (Taylor et al., 2019) involves an adapted formulation model and a range of imagery techniques including metacognitive approaches, imagery rescripting and positive imagery. It has previously been tested in a small case series and was acceptable (Taylor et al. 2020). The recruitment target was achieved and excellent uptake of sessions (100% of sessions attended) and good retention (100% retention during therapy. There were no significant adverse effects.

## OBJECTIVES AND RESEARCH QUESTIONS

Our main aim is to assess whether it is feasible to conduct a randomised controlled trial (RCT) to examine the (clinical and cost) effectiveness of an imagery focused psychological therapy in psychosis. This feasibility study is an essential step before a definitive RCT with a greater number of participants.

Research Questions:

1. What number and percentage of eligible patients/service users consent to the trial (recruitment)?
2. What is the level of engagement with adherence to the iMAPS intervention (therapy sessions attendance measures; therapist fidelity)?
3. What completion and data quality rates can be achieved (data completion and retention of participants)?
4. What estimates of effect sizes (if any) are present (acknowledging this is a feasibility trial)?
5. What are service users view regarding acceptability of i) participating in the trial, ii) participant’s views and priorities for potential iii) acceptability of al the primary outcome measures used and iv) of receiving iMAPS therapy (adherence to intervention protocol)?
6. What is the estimated sample size for a fully powered trial to evaluate the effectiveness of iMAPS (relative to usual care)?
7. What is the range of services used by participants and which are likely to be key cost drivers to consider for the main trial?
8. What is the range of health benefits and are they covered by the EQ-5L health status questionnaire?

We will also establish the format of finalised and refined treatment manuals and design of a future definitive trial.

## Trial Design

24-month feasibility randomised controlled trial comparing treatment as usual plus iMAPS therapy, versus treatment as usual, with a 2:1 allocation ratio. The study is funded by the UK National Institute for Health Research as part of a rapid call for Mental Health projects in the North of England to be conducted within 24 months. The study therefore has a nine-month recruitment window to recruit 45 participants. As this is a feasibility study, we aim to find out from service users/participants who are allocated to receive the iMAPS therapy about their experiences of taking part in the trial, receiving the therapy and suggestions for improvements. Therefore, our study design ensures that most of our service users in the study will be allocated to iMAPS therapy by using a 2:1 allocation.

# METHODS

Participants, Interventions and Outcomes

## Study Setting

**Setting:** NHS Early Intervention (EI) in Psychosis and Community Mental Health Team services, including inpatient wards Pennine Care NHS Foundation Trust (Greater Manchester). Forty-five participants will be recruited in Pennine Care NHS FT (30 in the iMAPS plus usual care arm and 15 in the usual care arm). The recruitment rate will be five participants per month, for approximately nine months.

## Eligibility CRITERIA

**Inclusion Criteria**

**Participants:**

1. Meeting criteria for a schizophrenia-spectrum diagnosis (ICD-10 codes F20, F22, F23, F25, F28, F29; ICD-11 codes F20, F22, F23, F25, F28, F29; ICD-11 codes 6A20, 6A21, 6A23, 6A24, 6A2Y,6A2Z) AND Score of 3 Mild or above on P1 Delusions or P3 Hallucinations on Positive and Negative Syndrome Scales (PANSS).

**Or**

a criterion level of positive symptoms severity indicated by a score of > 3 (mild symptom present) on either the delusions (P1), hallucinations (P3), grandiosity (P5) or suspiciousness (P6) items of the PANSS in the previous week (this is usually the operational criteria to under the care of early intervention psychosis team)

And/or the psychosis transition criteria of the CAARMS

1. aged 16 and above
2. Identifying a distressing image^[[1]](#footnote-1)^ (Rated 50% distressing or above) related to the psychotic experience scoring 3 or above on PANSS. The participant will self-report the image as distressing (e.g. Have you had a distressing image over the past month? Yes/No What would you rate the distress over the past month from 0-100?.
3. capacity to give informed consent,
4. under the care of an NHS mental health team the study is recruiting from
5. with a keyworker/access to a duty team worker.

**Exclusion Criteria**

1. primary diagnosis of alcohol, substance misuse disorder, or bipolar disorder (affective psychosis)
2. Secondary presenting difficulties such as severe addiction, acute suicidal risk, dementia, neurological disorder.
3. developmental disability (moderate to severe learning difficulty)
4. acquired brain injury/organic syndrome
5. currently participating in physical or mental health treatment studies or receiving psychological therapy
6. unable to complete the measures in written English (due to assessment battery psychometric validation in English)
7. in forensic settings
8. unmanageable level of risk of violence to researchers or clinicians (harassment behaviour – stalking).

**Qualitative Study – Participants**

**Inclusion Criteria**

1. Capacity to provide informed consent for interviews
2. Consent to have interviews recorded.
3. Participation in iMAPS-2, with at least 4 weeks involvement in iMAPS-2 post randomisation
4. Sufficient English language proficiency to take part in qualitative interviews or agreement to the use of an interpreter.

**Inclusion Criteria**

**Qualitative Study – Clinician Referrers/Staff**

1. Consent to have interviews digitally recorded
2. Experience of referring at least one service user to the iMAPS-2 Study

**Inclusion Criteria**

**Qualitative Study – Imagery focused therapy -Therapist Interview Study**

1. Consent to have interviews digitally recorded
2. Experience of treating at least one participant on an imagery focused therapy for psychosis trial

A purposive sampling approach will be utilised for the qualitative interviews, recruiting on the basis of a vary of factors e.g. gender, first episode psychosis team vs Community Mental Health Team, ethnicity, etc.

## Trial procedures

Service users who meet eligibility criteria and are under the care of the NHS Trust, will be invited to complete a number of measures at baseline, 16 weeks and 28 weeks follow ups. The service user will meet with a research assistant at the time intervals above to complete an integrated interview and a number of self-report measures. In addition to face to face appointments, the assessments may be conducted via NHS approved MS TEAMS or Attend Anywhere “Telehealth” video platforms. At the same time as giving consent to take part in the main trial and randomised to either usual care and iMAPS therapy or usual care, participants will be able to consent to being contacted by another researcher on the project, (via their preferred method) to provide additional information about the qualitative interviews which could take place at any point from end of therapy to end of study.

Figure 1. Participant Timeline

Potential participants are contacted by their care -coordinator or self refer to the research team, check meet initial eligibility criteria, given PIS, at least 24hrs to consider taking part, then re-contacted

RA arranges to receive informed written consent and undertake eligibility screen. Feedbacks outcome.

If eligible, appt to complete baseline outcome measures

30 participants are randomised to iMAPS therapy and usual care

15 participants are randomised to usual care

RA meets with participants 16 weeks post randomisation to complete outcome measures

Staff and patients who agreed to take part in interviews contacted and interviews arranged throughout this period

RA meets with participants 28 weeks post randomisation to complete outcome measures

## Recruitment

(Strategies for achieving adequate participant enrolment to reach target sample size)

The Chief Investigator will support Research Assistants to visit clinical services to present the trial, engage clinicians in the trial and to provide copies of the participant information sheets and posters. Members of the clinical care team will seek consent from potential participants to be contacted by the researchers. Service users/patients who consent for details to be passed to the trial team, will be given a Participant Information Sheet (PIS) by either a member of the care team, by post/email or by the RA and given the opportunity to ask any questions they may have. The RA will contact the service user/patient at least 24 hours (often longer) after being given the PIS to discuss whether they would like to take part in the study. The Service User will be given the opportunity to ask any further questions they may have and will be offered time to consider taking part should they wish. Individuals who give informed, written consent will be screened for eligibility. Individuals who are eligible, complete the assessment and are randomised will enter the trial.

iMAPS-2 will also accept self referrals from Service Users at Pennine care NHS FT, in Boroughs which the trial is actively recruiting from. Posters will be displayed in NHS mental health services to advertise the study. A study website will also be available. Participant information sheets will also be available, but the main aim of the website is public engagement and dissemination. If an individual self refers, we will ask their consent to contact their mental health team/mental health professional involved in their care to check eligibility criteria and obtain information for risk assessment purposes and to ensure participation is not contraindicated.

The key screening is for the reporting, of distressing image related to a psychotic experience which would score 3 or above on PANSS P1 or P3 in a brief discussion with the patient. The RA will then complete an eligibility review with the PI. After the eligibility review, the potential participant will be contacted by the RA to inform them of whether they are eligible to participate in the study.

During the consent and eligibility screening meeting, the member of the research team will complete the following measures with the participant;

- Demographic Information
- Participant Details (contact information, GP, etc)
- Defining “mental imagery” with examples (using the Spontaneous Use of Imagery Scale (SUIS) (Reisberg et al., 2003) (“try to think of a lemon in the form a picture that you can see in your mind’s eye”). Participants will then be asked a series of questions about any intrusive images in relation to aspects of psychotic experiences. “Do you ever have any intrusive images or pictures that just pop into your mind when you have thoughts about (delusion or hallucinatory experience)? (Additional information will include content of the intrusive image, and context; image distress, threat, vividness, controllability, frequency all rated from 0-100 not at all to extremely) Frequency in last week and month. Intensity of emotion from 0-100. Meaning of the image.
- Key questions from PANSS P1 Delusions and P3 Hallucinations to confirm eligibility

If eligible, an appointment will be made with the RA to complete the baseline assessment (either face to face or remote). If they are not eligible, the potential participant will be contacted by the RA. Those individuals will be informed of the reasons for not being eligible for the study. For example, not experiencing mental images which are particularly distressing. The reason for ineligibility will be framed in a positive way, so as to minimise disappointment and emphasis that it is the study that is not suitable for them e.g. that the person is coping well with their mental images or psychotic experiences. The potential participants care coordinator will also be informed that they are not eligible to take part. If frequency or severity of the Service User’s psychotic experience should change they can be re-referred to the study for re-consent and screening. The Service User and their Care Coordinator will be informed of this. We will send participants a thank you card, after each of the three assessments as a token of appreciation for taking part. For participants who withdraw from the therapy arm and/or assessments as part of the trial, on withdrawal we will ask them if they wish to be sent a thank you card. If they decline, they will not be sent a card.

Our service user co-applicant Thom (who was a patient in the case series study) has advised the recruitment strategy should include examples of imagery in different senses (*e.g. “I get a visual flashback to something bad which happened”*), and negative beliefs about self and other examples (*e.g. “I am vulnerable, Others are hostile”*), to make it clearer to potential participants the sorts of difficulties the therapy is trying to help with.

Professional referrers will be invited to take part in interviews about the experience of referring participants to iMAPS-2. Those who are interested in taking part will be given a PIS and opportunity to have their questions asked and answered. They will be recontacted after 24 hours (often longer) with more time given if needed. Those who give written informed consent, will be invited to take part in the interviews.

Therapists who have offered therapy to a least one participant on an imagery focused therapy for psychosis study will be invited to take part in interviews about the experience of delivering therapy to participants. Those who are interested in taking part will be given a PIS and opportunity to have their questions asked and answered. They will be recontacted after 24 hours (often longer) with more time given if needed. Those who give written informed consent, will be invited to take part in the interviews.

## Patient Identification

Potential participants will be identified by a range of strategies. The RAs are embedded within the clinical team and the identification of service users may be by direct RA identification and service user approach and clinical consent to contact. The Ras will also identify potential service users who may be eligible with staff assistance using the inclusion and exclusion criteria provided to them. These staff members will then ascertain whether participants are willing to receive information about the study and to be contacted by a researcher. Potential participants can also self refer. They will give consent for their care team to be contacted to check eligibility and risk and the process will follow the same steps as any other participant.

## Consent And eligibility

Service Users/Patients who are recruited via the clinician-referral recruitment route, will hear about the study from their clinician, who will take to them about the study and discuss the trial with them. Any questions will be answered, they will be given a participant information sheet (PIS) and will be asked to give their consent to be contacted by the research team and to pass on risk information to allow a risk assessment. A research assistant will communicate with clinician colleagues to identify potential participants, be passed on contact details for potential participants, and we will ask the clinician a number of risk questions to ensure we have complete risk assessment documentation. The research assistant will then contact potential participants directly to talk about the study with them and answer any queries about participation.

There is also a self-referral route to participation. In these instances, the research assistant will gain clear informed verbal consent to contact their care co-ordinator (CCO)/clinician involved in their care to establish if they meet criteria to take part (inclusion criteria) and to share risk information to complete a risk assessment. The research assistant will also contact the care team to explore if they have any concerns regarding the service user/patient’s participation and their capacity to consent to take part. If the service user/patient may potentially meet the inclusion criteria for the trial and has an ongoing interest in taking part, the research assistant will contact them to arrange a meeting to take their informed consent and undertake the eligibility screen.

Service Users/Patients interested in taking part will have as long as they wish (while the trial is open to recruitment) to think about the information sheet before being contacted by a member of the research team, with at least 24 hours. The Participant Information Sheet (PIS) outlines what participants are asked to do, how their information will be utilised, and the risks and benefits of taking part in the trial.

At the first appointment with the service user/patient interested in taking part, the research assistant will check they have been given a PIS and had a chance to review it. The service user/patient will have another opportunity to re-read it and have any further questions answered. It will be reiterated to all service users/patients interested in taking part that participation is voluntary and that their usual care and treatment will not be impacted if they decline to take part. The right to withdraw and not take part anymore will also be reiterated and any withdrawal will have no impact on their care and treatment.

Those who wish to participate, will give written informed consent. The original wet-ink signed copy of the consent form(s) will be retained in the Investigator Site File and a copy given to the participant. A copy of the consent form will be sent to the participants relevant care team to be on their clinical records (or a copy directly uploaded to the care record). Screening assessment for eligibility will then take place. If eligible, the initial assessment will be undertaken and the participant will be randomised. Participation in the study will begin. A similar process will be followed for referrers or clinicians or therapists who are taking part in an interview study (with the exception of not uploading copies/sending copies of consent forms to their medical records or writing to their GPs to inform them of their participation in the study). After each assessment timepoint is completed by the RA, we will contact the participant by phone 48 hours later to check on their wellbeing and ensure they are fine after the appointments.

A research assistant will determine (with support from the Chief Investigator Dr Christopher Taylor) if a participant has capacity to give informed consent and meets inclusion criteria. All researchers working on the study will attend the NIHR training on good clinical practice and informed consent.

Trained RAs will assess each participant’s capacity at each assessment interval (baseline, 16 weeks, 28 weeks and qualitative interviews where relevant) to determine whether the participant still qualifies as having capacity and thus is still able to provide informed consent to continue. However, the participant will not be required to sign further consent forms.

### Participant withdrawal

Participants in the study are free to withdraw from participating at any point, without giving a reason and without their legal rights or usual care being impacted. It is possible for a participant to with draw from receiving the therapy, but continue to remain in the trial for the follow up assessments, in essence, participants will be asked if they are willing to continue data collection assessments at 16 weeks and 28 weeks, if they wish to withdraw from the iMAPS intervention. While being reminded of their right to withdraw without giving a reason, we will politely ask if they are willing to give a reason, but remind them that this is entirely voluntary.

If continued participation for some reason is assessed by the researchers to be harmful in some way, then we may withdraw a participant. The Trial Steering Committee (TSC) which incorporates a Data Monitoring and Ethics function will review all instances of adverse events, regardless of whether they are judged to be attributable to the trial or interventions. Reviewing this information, the independent committee members will decide if a participant should be withdrawn from the study. All adverse events deemed related to study participation will be reported the Research Ethics Committee (See Section 9 for further details).

There is a possibility that individuals taking part may lose capacity to consent to continue participating in the study. In this event, we would pause their participation to see if they regain their capacity (acknowledging that capacity fluctuates in people with psychosis and may be temporary). However, if they continue to lack capacity, this may also mean they are no longer able to consent to ongoing participation in the study or for their data to be retained. In line with previous studies (including the iMAPS case series) we will seek informed consent in advance from all participants to retain their data if they subsequently withdraw because of loss of contact or are withdrawn by the study team due to loss of capacity

## Assignments of Interventions

**Allocation Concealment Mechanism**

## Allocation

**Sequence Generation**

Participants will be allocated in a 2:1 ratio to one of two arms within the RCT; usual care or iMAPS intervention plus usual care using blocks of random (but relatively short) length, stratifying by being a service user/patient under the care of a Community Mental Health Team or an Early Intervention Psychosis Team Health Teams (CMHT).

Allocation

Randomisation will be performed by SealedEnvelope, an online central randomisation service. The randomisation list will be generated by a statistician independent from the study team and held by SealedEnvelope

Delegated staff at sites with access to the online randomisation service will have to confirm the eligibility criteria before they enter the stratification information and being permitted to randomised. Then the system will allocate the randomised treatment which will be notified to the user via email. Allocation concealment (from both recruiting staff member and [potential] participant) will be ensured, as the system will not release the randomisation code until the participant has been randomised.

Participants will be allocated to one of two arms within the RCT, randomised (2:1) to usual care or iMAPS intervention plus usual care using blocks of random (but relatively short) length and implemented using the on-line sealedenvelope.com system. The parameters for the randomisation list using sealed envelope (<https://www.sealedenvelope.com/simple-randomiser/v1/lists>) will be prepared by Chris Sutton and Yvonne Sylvestre (e.g. treatment groups, block sizes, list length and stratification) however, the seed for the random number generator will be input by an independent statistician colleague at University of Manchester who is independent from the study team.

## (Allocation Implementation)

The responsibility for recruiting and randomising participants into the study lies with the Chief Investigator and staff at site with access to the online randomisation system which will be provided with a secure login to the sealedenvelope.com website.

## Blinding

To reduce detection bias, researchers undertaking assessments will be blind to the allocation. The success or otherwise of our attempts at blinding will be recorded. Where a research assistant is unblinded, another research assistant will make an appointment to undertake the assessment.

*Allocation concealment (from both recruiting staff member and [potential] participant) will be ensured, as the system will not release the randomisation code until the participant has been randomised which takes place after all baseline measurements have been completed*.

.

It is not anticipated that un-blinding of the research assistants will occur in the trial and it is not expected that there would be any reason for emergency un-blinding of the researchers. Any instances of unintentional un-blinding will be recorded.

The Trial Statistician will remain blinded to treatment allocation until after the Statistical Analysis Plan (SAP) have been sign off and the analysis of unblinded data commences.

## Outcome Measures (Feasibility, Clinical and Health Cost)

1. Recruitment rate (Number of participants consented into the trial and randomised. This will also include number of referrals per month; source of recruitment; number of patients (potential) participants contacted; number of participants screened for eligibility; number of screened patients who are found to eligible, reasons for non-eligibility or withdrawal of interest – where potential participants are happy to share these reasons).
2. Therapy Engagement % who drop out of therapy/% who did not receive treatment allocated. (This will include number of therapy sessions attended).
3. Therapist adherence to therapy protocols and supervision protocols. This will include assessing therapist fidelity against the Cognitive Therapy Rating Scale Revised (CTS-R) or the Cognitive Therapy Rating Scale for Psychosis (CTS-Psy) and the nested study developing an iMAPS fidelity scale (see Appendix 4).
4. Completion rates at week 0, 16 weeks and 28 weeks on widely used outcome measures of schema (Brief Core Schema Scales)(Fowler, Freeman, Smith, Kuipers, Bebbington, Bashforth, et al., 2006), imagery characteristics (VAS/MPIQ) (Holmes et al., 2016), and delusions (Psychotic Symptom Rating Scales; PSYRATS (Haddock et al., 1999)) and the overall measures completion rate. In line with guidance for feasibility trials, we have not specified a primary outcome, and in addition, the study seeks to identify a suitable primary outcome for the larger planned trial (Lancaster et al., 2004). Adverse events, quality of life and NHS service use will also be measured.
5. Standard deviations of normally distributed clinical outcomes
6. Reported breaks of assessor blinding, participant, and staff views on the trial acceptability (including randomisation) and possible changes to the intervention and trial protocol.
7. We will also include the EQ-5D 5L, a NICE recommended health measure utilised for QALY estimate and the ReQoL-10 for feasibility of economic analysis. We will record all service user and other relevant health economic data, using an adapted version of the Economic Patient Questionnaire (Davies, Lewis and Jones, 2007) which includes questions from the Client Service Receipt Inventory (CSRI).
8. Service users views regarding acceptability of i) participating in the trial and ii) of receiving iMAPS therapy assessed via qualitative interview. We will also interview a number of clinician colleagues about their experience of referring to the trial and feedback .
9. Health economic outcomes, via HE measures and service use questionnaire. A range of NHS and social care service use is collected, including inpatient admission, outpatient visits, A&E, primary, community and social care use.”
10. SAEs and AEs

**Full Trial Progression Criteria:**

We will progress to a full trial if we all our criteria below are classed as GREEN; if one or more is AMBER, we will investigate whether it is possible to make adjustments to the trial design to overcome any problems and, if that appears possible, progress to a full trial (including an assessment of the success of our adjustments in its internal pilot, where appropriate); if one or more is RED, we will not progress unless we can make the substantial amendments which would be necessary to make a full trial feasible.

1. Recruitment: GREEN: >=80% (n>=36) of target recruited; AMBER >=40 % (n=18-35)-< 80% of target recruited; RED <40% (n<18) of target recruited.
2. Retention: GREEN: >=80% of participants providing 16-week outcome data ; AMBER: >60%- <80% of

participants providing 16-week outcome data; RED: <60% of participants providing 16-week outcome data.

- 1. Adherence: GREEN>=75% adherence of participants attend at least 5 sessions of therapy, AMBER>=40% participants

attend at least 5 sessions of therapy; RED: <40% of participants attend at least 5 sessions of therapy (based on Jolley et al. 2015).

Assessment Measures

|  | **Procedures and Questionnaires** | **Mins** | **Baseline** | **16 Weeks Follow Up** | **28 Weeks Follow Up** |
| --- | --- | --- | --- | --- | --- |
|  | Demographics | 5 | X | As needed | As needed |
| **Psychosis Measures** | PANSS | 60 | X | X | X |
|  | PSYRATS | 15 | X | X | X |
|  | QPR | 5 | X | X | X |
| **Imagery** | Brief Imagery Interview  Visual Analogue Scales/MIPQ  Psychosis Imagery Questionnaire (PIQ) | 10  5 | X  X | X | X |
| **Schemas** | BCSS | 2 | X | X | X |
| **Trauma** | TALE | 5 | X |  |  |
|  | ITQ | 5 | X | X | X |
|  | BES | 5 | X | X | X |
| **Mental Health and Functioning** | BAI | 5 | X | X | X |
|  | CDS | 9 | X | X | X |
|  | WEMWBS | 5 | X | X | X |
|  | PSP | 10 | X | X | X |
| **Health Economics** | EQ-5D-5L | 2 | X | X | X |
|  | ReQoL-10 | 2 | X | X | X |
|  | Economic Patient Questionnaire | 10 | X | X | X |
|  |  |  |  |  |  |
| **Working Alliance** | WAI-client | 5 | 3^rd^ Session | 6^th^ | 9^th^ |
|  |  | 5 |  |  |  |
| **THERAPIST ONLY** | WAI-therapist | 5 | 3^rd^ Session | 6^th^ | 9^th^ |
|  | iMAPS therapy fidelity Checklist  CTS-R or CTS-Psy |  | A selection of therapy sessions will rated in supervision and by an independent external therapist |  |  |
| **Adverse Effects** | CGI-IP | 1 | Each Session |  |  |
|  | CGI-SP | 1 | Each Session |  |  |
|  | AEP | 5 |  | End of therapy or at dropout, if consent to send has been given in advance. |  |

Note: PANSS: Positive and Negative Syndrome Scale (Kay et al., 1987); PSYRATS: Psychotic Symptom Rating Scales(Haddock et al., 1999); QPR: Questionnaire about the Process of Recovery(Law et al., 2014); MIPQ: Mental Imagery in Psychosis Questionnaire/Visual Analogue Scales (Taylor et al., 2019); PIQ: Psychosis Imagery Questionnaire Taylor et al. 2022); BCSS, Brief Core Schema Scale (Fowler, Freeman, Smith, Kuipers, Bebbington, & Bashforth, 2006); TALE: Trauma and Life Events Checklist (Carr et al., 2018); ITQ: International Trauma Questionnaire (Cloitre et al., 2018); BES: Basic Emotions Scale (Power, 2006); BAI: Beck Anxiety Inventory (Beck et al., 1988); CDS: Calgary Depression Scale (Addington et al., 1993); WEMWBS: Warwick Edinburgh Mental Well Being Scale (Tennant et al., 2007); PSP: The Personal and Social Performance Scale (Morosini et al., 2000); EQ-5D-5L: The EuroQol 5-Dimenson 5 Level measure (Janssen et al., 2013); REQol-10: Recovering Quality of Life Measure (Keetharuth et al., 2018); Economic Patient Questionnaire (Shields et al., 2020); WAI-SR-client: Working Alliance Inventory – client version (Hatcher & Gillaspy, 2006); WAI-SRT-therapist: Working Alliance Inventory – therapist version(Hatcher & Gillaspy, 2006); CTS-R: Cognitive Therapy Rating Scale – Revised (Blackburn et al., 2001); Cognitive Therapy Scale for Psychosis (Haddock et al., 2001); Clinical Global Impression- Improvement Participant Version (CGI-IP) Clinical Global Impression - Severity Participant (CGI-SP) Version; Adverse Effects of Psychotherapy Questionnaire (Hutton, 2013)

We will also capture postcode and calculate the Index of Multiple Deprivation Decile by using the following website link:

<http://imd-by-postcode.opendatacommunities.org/imd/2019>

**Qualitative Measures**

We aim to undertake qualitative interviews with participants and with staff who refer service users/patients to the study. We anticipate this will help us capture any emerging changes in implementation, including potential facilitators and barriers to implementation. The Interview topic guide is included as part of the HRA documents submission and includes questions regarding the trial, including the acceptability and preferences around questionnaires being used (if these are “too many” or acceptable). The Participants Interview schedule has been developed based on a theoretical framework of acceptability (Sekhon et al. 2017) covering areas such as affective attitude, burden, ethicality, intervention coherence, opportunity costs, perceived effectiveness and self efficacy) with questions framed in straightforward language.

We also seek to interview therapists who have delivered an imagery focused therapy for psychosis, to help inform understanding of the delivery of the therapy and to help make refinements to the therapy manual.

**Inter-Rater Reliability Measures**

All assessment sessions will be recorded with informed written consent. This to ensure there is sufficient inter-rater reliability across the assistants when administering the PANSS and PSYRATS interview measures, as part of an integrated interview protocol.

**Details of therapy sessions Fidelity & iMAPS Fidelity Scale Development**

Therapy sessions are to be audio/video recorded. A number of these will be reviewed in supervision every few weeks to be rated against the CTS-R and/or the CTS-PSY (Haddock et al. 2001) in clinical supervision. We will also seek to develop an adapted imagery focused therapy fidelity rating scale, with elements specific to the iMAPS protocol. The aim of this nested study will be to develop an iMAPS therapy fidelity scale, investigate it’s psychometric properties and examine it’s utility in evaluating the adherence of therapists engaged in this type of therapy with people experiencing psychosis. Please see appendix 4 for brief outline of aims, methods and planned analysis.

We will identify an experienced therapist colleague outside of the trial team to be added as part of the study team (letter of access if needed, etc) and to rate a random selection of sessions for fidelity.

**Health Economic Analysis**

Descriptive statistics will be used to summarise the range of service use and costs and the range of health benefits. Costs and baseline data will be described to explore key service use/cost drivers. A similar approach will be used to explore the range of health benefits and how well the generic EQ-5D-5L correlates with other outcome measures in this participant group.

Data will be collected on the utilisation of primary, community and secondary care, including medications, admissions, A&E attendances and bed-days.

### Number of Assessments and Measures (Participant Burden)

The trial has been designed to minimise the number of interviews and self-report questionnaires. Recent clinical trials conducted in the UK involving people with psychosis have had a significantly greater number of assessments. For example, the [Youth Mental Health and Resilience Study](https://doi.org/10.1186/s12888-017-1206-5) had >24 assessments per assessment point, including 10 interviews or psychometric assessments and 3 brain imaging assessments); the NIHR [FOCUS Trial](https://dx.doi.org/10.1186%2Fs12888-016-0983-6) had > 26 assessments per timepoint including 8 interviews or assessments)

Our trial has minimised the number of assessment points and we have carefully chosen our measures to gather essential information. Previous clinical trials we have been involved with have included longer research assessment durations. This experience has demonstrated that with regular breaks and the offer of completing the assessment over two or more appointments, people with psychosis have found the assessment process acceptable. Participants will each receive £15 per assessment (£45) in total to reimburse them for their time and any travel. If they take part in the qualitative interview, they will receive an additional £15. Staff who take part in the referrrer’s interview will also receive £15 as an honorarium (assuming they take part in non-working time e.g. beginning or end of working day).

We estimate the informed consent process will take approximately 15-20 minutes. We will then undertake a screening assessment to confirm eligibility (see earlier section 3.4 Recruitment). This will be discussed by the RA with the PI and the outcome fedback to the potential participant. The initial baseline assessment will take approximately 2 hours. Participants will be invited to complete this over two or three meetings, with a mixture of face to face and/or online video assessment as part of the offer. Breaks will be offered on request and/or every 30 mins.

The end of therapy assessment and follow up assessment will begin with confirming continued informed consent, and then completion of the measures. There will be the same offer to complete over two or more meetings and breaks will be frequently offered.

## FOLLOW UP ASSESSMENTS

Participants who are randomised will be invited to complete a follow up assessment at 16 weeks (end of therapy assessment) and again at 28 weeks (follow up assessment).

## Withdrawal

Withdrawal from the study will occur if:

- The participant decides to withdraw from the trial for any or no-stated reason

## Lost to Follow Up

Participants who unable to be contacted at 16 week follow up will be contacted again at 28 week follow up, unless they have withdrawn (or been withdrawn for clinical reasons) from the trial. Those who we are unable to contact at 28 weeks follow up will be lost to followup.

## End of the Trial

The trial will end when the last participant has completed the final follow up (and qualitative interview, if they also consent). We will then complete the Declaration of the End of Trial and submit to the HRA REC. We will notify the REC of the end of the trial within 90 days of completion or within 15 days, if the study should end prematurely. Following this, the process for closing the trial at the sites be undertaken. A summary report will be provided to the REC within 12 months of the end of trial.

## INTERVENTION

**iMAPS.** Therapists delivering the iMAPS therapy approach (Taylor et al. 2019) will be trained and supervised by lead \applicant and co-applicant experts in psychological therapy for psychosis to deliver the expanded 12 sessions of individualised, personalised iMAPS intervention over a twelve-week therapy phase. Please see our published iMAPS treatment approach journal publication for more details (Taylor et al 2019).

The therapy includes novel change techniques including imagery re-scripting (imaging an image or memory and changing the ending to change the meaning) of past events, imagery re-scripting of flash-forwards, working with nightmares and creating positive imagery. Other techniques used to work with negative core beliefs and images are i) learning that pushing images away makes them come back stronger (image suppression and responding differently), ii) testing beliefs about images and schemas (behavioural experiments), iii) changing (manipulation) of images and iv) working with upsetting memories. The use of the “Safe Place” image, as the first introduction to imagery work, ensures that patients have a personalised strategy to reduce any distress experienced. There are clear distress protocols developed and agreed in advance with patients (including managing dissociation) before the imagery sessions begin (after a careful assessment and formulation).

Please see more details in Table 1.**Table 1** Outline of the iMAPS Therapy (Taylor et al 2019)

Training and Supervision by CT, JK with support from KB, AA

Table 1. *iMAgery focused therapy for PSychosis (iMAPS)*

| Phase of Treatment | Main approach |
| --- | --- |
| Assessment, Goals, Psychoeducation | Interview  Imagery measures – Visual Analogue Scale, Spontaneous Use of imagery Scale (SUIS), Image Diaries, Assessing different types of imagery, Assessment of schema – core schema, early negative schema, schema modes |
| Formulation & Case Conceptualisation | Shared psychological formulation |
| Intervention |  |
| Imagery CT Approaches  Safe Place Image | A real or imaged safe place, described in detail, across each of the senses that gives a strong sense of safety and happiness |
| Image Suppression &  Behavioural Experiments | Similar to thought suppression experiments and behavioural experiments within other areas of CBT but with a focus on images |
| Manipulation of Images | To show images are only a mental event – improve sense of control  Test any beliefs or appraisals regarding an image meaning you are “going mad” |
| Working with upsetting memories | Transformation  Provide a wider context by running image on past the worst point  Updating aspects of the image  Emotional bridge to past (also use as diagnostic imagery exercise – can identify key life events which link to core beliefs, current images and psychotic symptoms). |
| Imagery Rescripting Approaches  Imagery Rescripting past events  Imagery Rescripting Flash-forwards | Past events  Future Flash-forwards  Discussion of negative beliefs re self and others, schemas – Imagery rescripting to change future anticipated image |
| Working with night-time imagery | Updating aspects of the image, rescripting new endings |
| Creating Positive Imagery | Deliberately generating positive images of the future |

We will follow best practice guidelines in delivering iMAPS, however, video therapy (Telehealth) is becoming part of routine care. The feasibility of the intervention will be assessed by the number of sessions attended, cancelled and drop-out rates. Intervention fidelity will be assessed by a random selection of tapes being rated using an iMAPS therapy checklist. In addition, therapists will complete a standardised session record form to monitor session content, in terms of therapy milestones and strategies permitted within the therapy protocol.

The feasibility of the intervention will be assessed by the number of sessions attended, cancelled and drop-out rates. Intervention fidelity will be assessed by a random selection of video tapes being rated using an iMAPS therapy checklist. In addition, therapists will complete a standardised session record form to monitor session content, in terms of therapy milestones and strategies permitted within the therapy protocol. Therapists will also complete a “therapy session record”, which will briefly document the main focus and techniques used from the iMAPS protocol. Therapists will also assess images each session, and record imagery characteristics for the main images. Similar to iMAPS (Taylor et al. 2020) where participants were offered the opportunity to have a CD copy of a therapy session, where the therapist is happy to and the participant is happy to, we will offer this opportunity to participants to also record the therapy session on their mobile phone/smartphone, so they can listen back to it in-between sessions to help consolidate learning. This is recommended in several CBT protocols but not widely undertaken in routine practice in several services.

**Usual Care**

Usual care will involve interventions offered by an early intervention service or a community mental health team (fortnightly/monthly visits from social worker, mental health nurse, outpatient psychiatry reviews quarterly, medication, routine CBT). The study would not seek to restrict access in routine care to CBT. We will record during the trial by asking participants and by reviewing their clinical record and record any service users in the usual care arm of the trial who receive routine CBT or other psychological intervention. However, we know there are still significant barriers to NHS therapy. The 2020 English NCAP Psychosis National Audit collected data on access to CBT, defining a course of CBT as receiving just one session of therapy, (rather than the min. 16 sessions recommended by NICE). Even with this low threshold, only 49% of service users had this offer in EI. Secondary care services have historically had even poorer commissioner investment.

**ANCILLARY AND POST-TRIAL CARE**

The iMAPS therapy is designed to be a stand-alone psychological therapy. Participants who wish to access further psychological assessment and therapy will be signposted to accessing this via the local NHS clinical services which they are eligible to be referred to.

## ADVERSE EVENTS

***Definitions***

| **Term** | **Definition** |
| --- | --- |
| **Adverse Event (AE)** | Any untoward medical occurrence in a participant to whom psychological therapy/assessment or qualitative interview has been administered.  Non severe adverse events will be defined as a score of >3 (Agree quite a lot or a lot) on any item of the participant rated 26 item Adverse Events of Psychotherapy Questionnaire (AEP; Hutton, 2016; Pyle et al 2016) which is given to participants on either completion of therapy, or if they have given informed consent, sent to those who drop out or end therapy early. |
| **Adverse Reaction (AR)** | An untoward and unintended response in a participant to a psychological therapy/assessment or qualitative interview which is related to the therapy/assessment or interview administered to that participant.  The phrase "response to a psychological therapy/assessment or qualitative interview" means that a causal relationship between a trial therapy/assessment or interview and an AE is at least a reasonable possibility, i.e. the relationship cannot be ruled out.  All cases judged by either the reporting clinically qualified professional or the Chief Investigator (acting on behalf of the Sponsor) as having a reasonable suspected causal relationship to the psychological therapy/assessment or qualitative interview qualify as adverse reactions. |
| **Serious Adverse Event (SAE)** | A serious adverse event is any untoward medical occurrence that:   - results in death - is life-threatening - requires inpatient hospitalisation or prolongation of existing hospitalisation - results in persistent or significant disability/incapacity - consists of a congenital anomaly or birth defect   Other ‘important medical events’ may also be considered serious if they jeopardise the participant or require an intervention to prevent one of the above consequences.  NOTE: The term "life-threatening" in the definition of "serious" refers to an event in which the participant was at risk of death at the time of the event; it does not refer to an event which hypothetically might have caused death if it were more severe.  In line with an approach previously used (Pyle et al 2016) which was adapted from Klingberg et al 2012), in addition we will define serious adverse events as:   1. death by suicide 2. suicide attempt 3. suicidal crisis without attempt (rating 2 on item 8 of Calgary Depression Rating Scale for Schizophrenia CDSS) 4. severe symptoms increase (rating of > 6 on the patient or researcher rated CGI and CGI-I). |
| **Serious Adverse Reaction (SAR)** | An adverse event that is both serious and, in the opinion of the reporting Investigator, believed with reasonable probability to be due to the psychological therapy/assessment or qualitative interview, based on the information provided. |
| **Related and Unexpected Serious Adverse Reaction** | A serious adverse reaction, the nature or severity of which is not consistent with the effects or consequences of a psychological intervention. |

NB: to avoid confusion or misunderstanding of the difference between the terms “serious” and “severe”, the following note of clarification is provided: “Severe” is often used to describe intensity of a specific event, which may be of relatively minor clinical significance. “Seriousness” is the regulatory definition supplied above.

Our iMAgery focused therapy for Psychosis approach has previously been tested in a case series and no severe adverse events were reported. Building on work by others in psychological treatments for psychosis, we wish to carefully record and report adverse events of psychological interventions and wish to implement a protocol developed assessing and managing any such risk, which will be completed at end of treatment and at follow up by research assistants who are blind to the allocated group. We are adding the use of the CGI and additional criteria below.

This protocol will also be completed by clinicians during the intervention, that is each session, to complete an assessment of severe adverse events, for clinical purposes.

## Operational definitions for (S)AEs

**AEs**

All adverse events **related** to the psychological therapy/assessment or qualitative interview that occur between baseline and end of study will be recorded in the participant medical notes and in the appropriate section of the trial CRF.

**AEs that do not require reporting**

Any untoward medical occurrence that is **not** related to the psychological therapy/assessment or qualitative interview that has been administered.

**SAEs**

All SAEs **related** to the psychological therapy/assessment or qualitative interview occurring between baseline until end of study must be recorded on the SAE report form and emailed to Sponsor immediately and within 24 hours of the research staff becoming aware of the event. Refer to the SAE form for the information that will be collected for all SAEs.

Any change of condition or other follow-up information should be emailed to the Sponsor as soon as it is available or at least within 24 hours of the information becoming available. Events will be followed up until the event has resolved or a final outcome has been reached.

| **Email completed all SAEs FAO:**  [**chrisdjtaylor@nhs.net**](mailto:chrisdjtaylor@nhs.net)  [**researchdevelopment.penninecare@nhs.net**](mailto:researchdevelopment.penninecare@nhs.net) |
| --- |

SAEs must be followed-up until resolution and sites must provide follow-up SAE reports if the SAE had not resolved at the time the initial report was submitted.

On receipt of the SAE Report Form, the Sponsor will send an acknowledgement of the SAE. This acknowledgement will include an SAE reference number which should be included on all future correspondence regarding the SAE.

The SAE Report Form will be reviewed by the CI for assessment of causality. If the SAE is related to the psychological therapy/assessment or qualitative interview, the CI will review the expectedness in relation to the nature or severity of an event which is not consistent with the effects or consequences of a psychological intervention. In the case that the SAE is related and **unexpected**, the study team will submit a report to REC, HRA and the Sponsor within the expedited timeframes.

## Reporting urgent safety measures

The Sponsor or Investigator may take appropriate urgent safety measures (USMs) in order to protect the participant of a clinical trial against any immediate hazard to their health or safety without prior authorisation from the ethics committee. This includes procedures taken to protect participants from pandemics or infections that pose serious risk to human health.

Where the PI takes urgent action that is not consistent with the protocol to prevent harm to a subject on a trial, the PI must immediately inform the Sponsor and give full details of the measures taken and the decision making process surrounding the action(s) taken. The CI will inform the sponsor and REC of these measures immediately, but no later than 3 days from the date the actions were taken.

Written notification in the form of a substantial amendment is also required, which is anticipated within approximately 2 weeks of initial notification.

##

Risk will also be assessed and managed as described in the following sections:

### Risks associated with non-clinical (research) procedures

#### Assessment and management of increased distress

The interviews and questionnaires involve revealing personal and sometimes sensitive information in relation to current symptoms and mood, including suicidal ideation. The interview measures of symptoms, such as the PANSS and the CDS have used routinely in clinical services (e.g. Early Intervention in Psychosis Services), without reported adverse effects. In a research context, these measures have been used over 100 times in previous studies (Leucht et al., 2017), including several locally in Manchester and the North West of England. Dazzi et al (Dazzi et al., 2014) found in relation to assessing suicidal ideation that discussing suicidal ideation “may in fact reduce, rather than increase suicidal ideation”.

Before the trial commences, researchers will be fully trained in how to respond to any increase in distress. During the trial, they will receive weekly structured supervision from HCPC registered practitioner clinical psychologists, who will be directly contactable by phone during the participant interviews.

If the participant experiences significant distress, the assistant will stay with them until the distress subsides. The assistant will be trained in active listening and listening empathically, and will help them to arrange contact their care co-ordinator. If the participant’s distress is more significant or more severe, they will immediately contact their HCPC registered clinical psychologist supervisor for advice and instructions on how to proceed (see separate *Safe Working Practices Document*). As much as possible, appointments will be scheduled close to/alongside, their usual clinical appointments so team members will be available or at the team base/clinic rooms, so a duty worker or member of the care team will be available.

All participants will be given contact details for their NHS services and support organisations in the unlikely event they feel distressed after their appointment. They will also have contact details for the assistant and the study team in case they have any further questions after taking part. As with all trials, participants will be reminded that they are free to withdraw at any point, without providing a reason and that their usual care and treatment will not be affected.

#### Safety Concerns (Risk to Self or Others)

Our trial involves asking questions about psychotic experiences and some questions about suicidal ideation (e.g. in the CDSS). There are some psychotic experiences, such as persecutory delusions or command hallucinations which can increase the risk of harm to self or others. There is also evidence that some people with psychosis and diagnosed with schizophrenia may have a significantly increased risk of suicide. As our clinical trial focuses on working with this group of service users/patients, some participants may disclose and reveal information to members of the research team which suggests that there is an imminent risk to self or others (further details are in the Safe Working Practices Document).

In the majority of referrals/participants, the participant’s NHS clinical team will already be aware of the risk and will have shared this risk information at the initial risk assessment stage when referring the potential participant. In some situations, the NHS clinical team will be unaware of all the risk issues or they may disclose some risk issues to the research team for the first time. In all circumstances, participants will be clearly informed by all researchers working on the trial that their information and data will be held confidentially, but there are limits to that confidentially which include sharing any disclosures of imminent risk of harm to themselves or others. In those circumstances, the researchers would be duty bound to take steps to reduce this risk, which in most situations will involve sharing concerns immediately with the NHS secondary care clinical team/participant’s care co-ordinator who will take over the clinical management of the risk issues. The research assistants will undertake their duties at all times under the supervision of an experienced clinical psychologist in the assessment and management of this process, making sure that that the participant and the assistant are fully supported.

### Risks associated with therapy procedures

#### Adverse effects of clinical interventions

The iMAPS therapy approach has been tested in a case series and no serious adverse events have been reported. As outlined in section 8.1, we will utilise a robust protocol for assessing and managing any risk which become apparent (please earlier section for more details).

#### Burden of iMAPS therapy

The offer of iMAPS therapy is approximately 12 sessions of therapy, to allow for the interventions to be as brief as possible, but give sufficient time for the images and schemas to be worked on in therapy to achieve change. Our retention rates of a shorter intervention during therapy in the case series was 100%, suggesting that the approach is acceptable to service users/patients with psychosis.

## RESEARCHERS and CLINICIANS

### Risk of violence from participants

There is a small risk of violence for researchers who have contact with participants (including assault, threatening behaviour, verbal abuse, etc). All the research team (assistants and clinical psychologists) will follow the Pennine Care NHS Foundation Trust Lone Working Procedure. The risk assessment forms will be collaboratively completed by the assistant and the NHS care team of the potential participant, to ensure all relevant risk information has been shared before meeting with the potential participant. Appointments for assessments or therapy will be undertaken in NHS clinic settings, or where a risk assessment highlights it is safe to do so with low risk, at participants homes, with a safe visiting. Any referred potential participants, who on discussion with the clinical team are identified as presenting with an unmanageable risk to the assistant or clinician will not be able to take part (e.g. risk of stalking or risk of violence that cannot be safely managed in an NHS clinic setting).

The assistants and clinicians will be given full induction regarding safety and alert measures in the NHS clinic rooms and will follow safe visiting protocols if visiting patients at home. This includes informing a colleague of their planned visit and address of visit in advance, calling to confirm they have arrived and when the appointment will begin, calling to confirm they are safe, the use of a discreet phrase to alert the colleague of an emerging risk situation and agreed procedures to escalate the risk if the assistant or clinician does not phone by an agreed time i.e. if unable to contact the researcher or clinician and unable to contact the participant, a call to police). The supervisor and research team will be available to be contacted throughout during such visits and appointments. All research staff will be trained on the safe visiting procedure and policy, reviewed weekly during the trial team meeting. Any issues or failure to follow the procedure will require further training and repeated failure to follow policy will result in HR escalation as needed.

The research team each have mobile phones, which will be fully charged, with the safe visiting number stored on the phone and also printed on the back. The research team will offer safe visiting on call cover for colleagues undertaking visits. The research team will all receive NHS training on breakaway techniques, de-escalation and managing violence and aggression. All research team members will be encouraged to follow their instincts and not go ahead with an interview or meeting if they feel at all uneasy.

### Vicarious trauma

The process of undertaking a detailed assessment (either research assessment or therapy assessment) involves a small risk of vicarious trauma. This is where hearing about distressing traumatic events in the lives of participants has a secondary emotional impact on them. All members of the team will receive regular supervision from experienced clinical psychologists. Self-care is a standing agenda item and supervisors will carefully monitor for any signs of distress. Where there are signs of stress or distress, we will pause recruitment and encourage colleagues to seek support (this could be from occupational health, the Trust Staff Support Service or their GP as indicated).

## Sample Size

As this is a feasibility trial, it is not powered to detect between-group differences. But we will seek to estimate recruitment and retention parameters as well as the SD in outcome measures with reasonably good precision. Recruiting 45 participants will allow us to estimate the retention rate at end of therapy via a 95% (exact binomial) confidence interval, with width no greater than 25%, assuming retention is at least 80%.  It will also suffice for the estimation of the SD, although the sample size (expected minimum 36 participants with outcome data) is towards the lower end of the sample size recommended (Julious, 2005; Sim and Lewis, 2012).  This is a recruitment rate average of five participants per month over nine months (with 2.0 WTE Research Assistants). The study will not be powered to detect between-group differences, however, the study will test participants willingness to be randomised to an imagery focused therapy trial with a control arm. But we will seek to estimate recruitment and retention parameters as well as the variance in outcome measures and the sample size will suffice to meet these and other project objectives.

In the participant’s qualitative study, we will seek to recruit 15-20 participants to take part in qualitative interview about their experiences of participating. In the clinicians/referrers interviews, we will seek to recruit 10 referrers to talk about their experience of referring to the trial. The participants will receive £15 as a token of thanks for taking part in the interviews.

## ANALYSIS METHODS

**Quantitative analysis**

The analysis will follow the intention-to-treat (ITT) principle. Clinical outcome data will be sought and all available data included in the analyses regardless of adherence to the intervention during the time scale of the trial. Participants will be retained and analysed in their allocated arm. The CONSORT 2010 extension for reporting pilot and feasibility studies will be followed.

As this is a feasibility study the analysis will be mainly descriptive and focus on confidence interval estimation rather than hypothesis testing.

A statistical analysis plan (SAP) will be presented to and agreed with the programme steering group prior to the allocation codes being revealed and commencement of any data analysis. Yvonne Sylvestre, with support from Dr Chris Sutton, (University of Manchester) will undertake the analysis of the trial data in line with the SAP which will be approved by the Trial Steering Committee prior to any analysis being performed.

The number of participants screened, eligible, consented, randomised and withdrawn from the study will be summarised and reported in a CONSORT flow diagram (Schulz, et al., 2010).

Demographic and other baseline characteristics will be summarised by randomised groups. Summary measures for the baseline characteristics of each group will be presented as mean and standard deviations and/or median and interquartile range (as appropriate), and as frequencies and percentages for categorical variables.

Key feasibility outcomes; recruitment rates, retention at the end of trial therapy and adherence to therapy (defined as the proportion of participants that attend at least 5 sessions) to assess the progression criteria to conduct a definitive trial will be presented as point estimates with 95% confidence intervals (using exact binomial methods).

Other feasibility outcomes such as unblinding rates, number and proportion of therapy sessions attended or cancelled, or the proportion of participants withdrawing from therapy will be summarised and reported in the same fashion.

The mean and standard deviation for the continuous clinical outcomes (BCSS, VAS/IMPIQ and PSYRATS) will be reported at baseline, 16 and 28 weeks which will inform sample size calculations of a future definitive trial. Differences in clinical outcomes will be estimated using regression-based approaches (primarily linear modelling) as an initial assessment of effectiveness. Results will be presented as effect estimates with 95% Confidence Intervals

The number of safety events (e.g., AEs and SAEs) (as outlined in section 3.17) and number (and proportion) of participants reporting at least one safety event will be summarised by treatment arm. Summary measures will be number (%).

**Qualitative Analysis**

In the planned qualitative studies, Dr Christopher Taylor and Professor Katherine Berry will supervise a researcher to undertake, conduct and record the participant and referrer’s interviews, transcribe them and analyse them utilising reflexive thematic analysis.

The therapist interview study will be supervised by Dr Mike Jestico, Lecturer in Psychology, University of Leeds (expert in qualitative research methods) and Dr Christopher Taylor, Chief Investigator.

Due to the exploratory nature of this research, it was decided that thematic analysis would be the most appropriate method of analysis to use for the data obtained. Interviews will be audio recorded and the recordings will be transcribed by a member of the project team or an administrator and uploaded to a software program to support analysis (e.g. Excel or NVivo). We may also use MS TEAMS (which is NHS approved) to record the interviews and make use of the transcription function (with careful checking and amendment of the transcription to ensure it is accurate).

We may also choose to accelerate the transcription process using a secure approved data transcription service. In phase 1, the data will be reviewed through reading and re-reading transcriptions and interviewer reflections and noting down initial ideas. Phase 2 will involve generating initial codes from each interview. Each of the interview transcriptions will be coded individually, line by line, to identify all relevant features of the data and codes collated systematically. In phase 3, the resulting codes will be grouped together into conceptual categories with shared or allied meaning. Phase 4 will involve reviewing these themes, by presenting them to the research team, checking that they work with the coded extracts and with the dataset as a whole. This iterative process will continue until in the 5th phase, the themes will be named and defined. Alternative interpretations will be discussed until consensus is reached for the final coding structure. This will include the study team and members of our public and patient involvement group. The 6th phase will involve choosing compelling extracts that help explain and justify the themes, and relating these back to the main research question (Braun and Clarke, 2006).

## DATA HANDLING

Case report Forms (CRFs) will be used to collect the study data. The PI is responsible for ensuring the accuracy, completeness, legibility and timely provision of the data recorded in the CRFs. Only the Investigator and those who have signed the Delegation Log and have been authorised by the Investigator should access the study database to enter or change data in the CRFs. At the end of the trial, all CRFs will be retained, site access to the study database removed, copy of patient data for the study provided and preparation for archiving on behalf of the Sponsor.

## CRFs as Source Documents

The CI is responsible for maintaining a comprehensive and centralised filing system of all study-related (essential) documentation, suitable for inspection at any time by representatives from applicable regulatory authorities. Elements should include:

• Patient files containing copies of completed e-CRFs, informed consent forms, and supporting copies of source documentation (if applicable)

• Study files containing the protocol with all amendments, copies of pre-study documentation and all correspondence to and from Research Ethics Committees

• All original source documents supporting entries in the CRF must be maintained and be readily available

## DATA Monitoring

## Data Collection, Source Data and Confidentiality

**Contact Information**

During the study, contact details (address, email, telephone and/or mobile phone numbers) will be used to contact the individual about the study. Contact details will be stored securely in a locked filing cabinet or on password protected computer files and kept separately from the other data obtained. Potential participants may be contacted via email or phone to provide information about the research, to answer any questions about the study, and to book and/or reschedule appointments for interviews. Any emails will be sent via nhs.net, a national NHS email system with a high level of security and encryption feature. Any emails sent will be in compliance with Data Protection Legislation and Trust Email Policy. Staff and organisations may also send emails to the researchers with contact details for individuals who have expressed that they would like to be contacted about the study. Patients will also be asked if they consent to their name and preferred contact details being retained for information about the study findings or information about future research.

**Quantitative Data Collection & Storage**

Data will be recorded on paper copies of the outcome measures. All data will be stored marked with a participant’s unique identifying code. Where follow-up outcome measures are being collected over MS TEAMS/the phone (if necessary), the research assistant will complete the paper copies, based on the responses provided by the participant.

All data will be entered into a database in a secure folder on an NHS Central ICT Drive by a researcher, and paper copies will be retained by Pennine Care NHS Foundation Trust. Paper data will be kept in a secure locked filing cabinet at the Trust. Personal data (e.g. name, contact details) will be separated from the research data in a locked filing cabinet on NHS premises. Consent forms will also be stored in a separate locked filing cabinet at NHS premises. During the study, contact details (address, email, telephone and/or mobile phone numbers) will be used to contact the individual about the study (e.g. provide study information, answer any queries, appointment booking/reminders, home visits). Contact details will be stored securely in a locked filing cabinet or on password protected computer files and kept separately from the other data obtained.

**Therapy Session and Qualitative Data Collection & Storage**

Interviews will be digitally audio recorded using encrypted devices and field notes taken by the researcher. Permission to record interviews will be sought on the Participant consent form. Recordings and transcripts will be stored on a password protected NHS computer and ICT Secure Central Drive. During the transcription process any identifiable information mentioned in the interview will be removed from the interviews. Transcription will be carried out by the research team or administrative staff employed by the NHS or an experienced, university/NHS approved, transcription company (e.g. 1^st^ Class Transcription – upload direct to their secure service <https://www.1stclass.uk.com/>faqs.html

*“We provide our client area as a pure extranet, which means that when you log in you can only see your own files and no other clients'; the same applies to all our clients. When uploading the files, they are transferred over a 256-bit SSL-encrypted channel which ensure that the transfer is secure. The extranet itself resides on a dedicated server to which only we have access - there is no public access to the hardware at all. The server is also security patched to the latest standards and we can supply information for any public sector or private organisation seeking confirmation of the standard we work to.*.

All personal data and audio/video recordings will be destroyed when no longer required when data lock is complete and the trial has formally ended (other than consent forms and enrolment log for audit and governance by regulatory authorities and contact details who wish to be contacted about future research opportunities). Anonymised direct quotes may be used in published articles or in presentations arising from the research. Care will be taken to ensure that no identifiable information is included when using direct quotes. Permission to use direct quotes will be gained via the consent forms. We will also seek informed written consent at end of therapy for a small number of video clips where a specific therapy technique has been useful to be retained for training and accessible by the chief investigator. We will show the clip to the participant and request informed consent for the clip to be used in future training of professionals in the therapy. However, this is again optional and participants may decline to give their permission without this impacting participation in the trial.

Clinical therapy sessions, research assessments and qualitative interviews will be audio-recorded with participant consent, using encrypted and password secure audio recorders with informed consent (or via video recording on Trust encrypted laptops). The devices will meet Pennine Care NHS FT IG standards. This is to ensure that we can i) check therapy fidelity (the quality of how well the psychological therapy is delivered – this is often done in routine clinical practice, as part of CBT accreditation procedures) ii) allow accurate scoring of interview responses iii) transcription and analysis of qualitative interviews.

In requesting specific informed written consent to video/audio record the therapy sessions, to be viewed by supervisors in clinical supervision and therapy protocol supervision. This is common practice for accredited cognitive behavioural therapists. It is optional, so participants may decline for some sessions or all sessions to be recorded. They will be stored securely on a secure folder on the Pennine Care ICT Server or securely on NHS Onedrive and only accessible by relevant members of the research team. We will also offer participants the opportunity to record their therapy sessions on their own device (e.g. mobile phone), assuming they are willing to do so and their therapist is willing to do so. This is for the purposes of listening back to the session during the week before their next appointment. It is a practice mentioned in some CBT protocols. An additional option would be to store the recording on secure NHS Onedrive and share a link with the participant to “view only”. The link and file could then be deleted at end of therapy.

Any personally identifiable information will be stored securely. Pseudo-Anonymised data (such as a database of questionnaire scores, linked to anonymous participant ID, will be held in secure password-protected storage on NHS or University computers). Anonymised and password protected data may be shared via email within the research team or via the NHS OneDrive, for statistical analysis purposes and with members of the TSC, for safety monitoring in line with the DMEC duties of the TSC. The annoymised transcripts from the interviews as part of the Qualitative studies will securely transferred via password protected documents or via OneDrive for analysis at the University of Manchester or at The University of Leeds using a software (e.g.NVivo) or traditional theme highlighting in a Word/Excel. The data generated as part of the study will be held securely and in line with NHS policies on Confidentiality, Data Protection Legislation and the British Psychological Society Code of Human Research Ethics guidelines and principles of Good Clinical Practice. The appropriate individuals from the research governance departments of Pennine Care NHS FT, may access the signed consent forms, if required as part of any audit but confidentiality will be respected at all times. Prior written agreement from the Sponsor or its designee must be obtained for the disclosure of any confidential information to other parties. This is made clear in the participant information sheet and participants give their informed consent for this access to happen in these circumstances. All investigators and trial staff involved must comply with the requirements of the Data Protection Act 2018 with regard to the collection, storage, processing and disclosure of personal information and will upload the Act’s core principles. The Sponsor’s host institution will be the data controller.

At a participant’s request, we are willing to share a summary of the assessments with their care co-ordinator or care team and more detail if requested. Similarly, it is common practice to share with consent progress in therapy with a participant’s care coordinator and we will discuss with the participant the level of detail they feel comfortable sharing about what was covered in the iMAPS therapy sessions.

**Personal Information**

All data collected during the study will be stored on password protected University or NHS computers and in a locked filing cabinet in a locked office on the University or NHS premises. Consent forms will be stored in separate locked filing cabinets to the data at the University of Manchester or on NHS premises. Personal data (e.g. name, contact details) will be separated from the research data in a locked filing cabinet on NHS premises. Postcode data may be shared with the University as part of planned analysis. However, because data will be collected at different times, it is necessary to keep all of the data together. There will be a password protected file which will be kept separate to all other documents that only the research team will have access to. This file will match participant name to the unique study number. As soon as all data has been collected, this file will be electronically deleted.

**Permitted Access**

Individuals from Pennine Care NHS FT Research and Innovation Department and, regulatory authorities, will have access to study data and material for monitoring and auditing purposes.

**Confidentiality**

Confidentiality of all personal data will be managed in accordance with Pennine Care NHS Foundation Trust policy. All participants will be assigned a unique study code which will be assigned at study entry for use on research documents. These numbers will be stored on a password protected file on a NHS computer. Any data held electronically will be password protected and unique participant codes will be applied to each participant in the study.

**Data Storage**

The research data will be managed and stored in accordance Pennine Care NHS FT data management policies. Individuals’ data will not be used for any other purpose than that stipulated in the participant information sheet and consent form. The storage of research data will comply with the Trust’s policy of storing data so will be retained 5 years after the last publication from the study. At the end of the retention period, paper will be destroyed using paper shredders. Electronic data which is not archived will be deleted.

**Participant Rights**

The participant will be informed that they can discontinue their participation at any time, without detriment to any treatment that they receive. This will also be emphasised in the participant information sheet and consent form. The participant information sheet will ask for permission to retain and use data already collected if a participant loses capacity to consent or withdraws from the study.

In line with NHR data sharing policy https://www.nihr.ac.uk/documents/nihr-position-on-the-sharing-of-research-data/12253, we are also asking for informed consent to share anonymised data with other researchers, at the discretion of the research team. This would be after the research team have completed their planned publications. Other researchers may submit a request to undertake a secondary data analysis, and so we would request from them a protocol and data analysis plan as part of such a request. Participants may decline for their data to included and their data will not form part of an end of study anonymous dataset prepared for future requests. Further details are in the Protocol Data Management Plan Appendix.

### Study Site Staff and Training

All members of the site research team will receive full training on this protocol, including but not limited to our consent procedures, our risk assessment (and actions) procedures and utilizing the research measures. This will also include training on ensuring high levels of data quality, keeping data secure in line with information governance procedures and the Data Protection Act, high standards of record keeping and maintaining confidentiality. All research staff working on the project will complete Good Clinical Practice Training this before undertaking research activities.

## Data Monitoring and Quality Assurance

The study will be subject to the audit and monitoring regime of the Research and Innovation Service, Pennine Care NHS Foundation Trust.

The trial will use the Trust’s Central ICT Drive (G Drive), with an individual restricted access Folder, only accessible to members of the research team. Access is strictly controlled as per GCP, individual access is only granted (with a specific ICT request) after specific training has been completed and documented. As such, risk is minimised and comprehensive control of the data is maintained.

An Excel file will be created for the capture and storage of participant data. The Pennine Care ICT Drive is backed up daily. Data entered into the Excel file will be pseudo-annoymised.

## Data Handling and Recording Keeping

Data will be checked for errors, inconsistencies and omissions by other assistants and the PI at random intervals. If missing or questionable data are identified, the PI will request that the data be clarified. Pre-defined checking routines will be applied to all batches of data to ensure complete, accurate data are provided for statistical analysis and reporting.

## Archiving

Essential documents will be retained for at least 5 years from conclusion of the trial (submission of the end of study report) for possible audit or inspection purposes. Documents will be securely stored and access restricted to authorised personnel. Archiving and destruction of essential trial documentation will be authorised by the Sponsor.

Participant identifiable information (participant details) will be kept for up to 6 months from the protocol defined end of study point. The consent forms will be retained for up to 5 years. All other documentation will be kept for 10 years to allow for substantiating the research findings or for the length of time stipulated by the peer review journal the research is submitted to.

## MONITORING

A Trial Monitoring Plan will be developed and agreed with the trial team, including some members of the Trial Management Group (TMG) and TSC. The plan will be based on the trial risk assessment, which may include on site monitoring.

This will include internal monitoring of RAs data collection and inputting i.e. how well in adheres to GCP guidelines, and correspondence between paper and electronic data entry.

## Trial Steering Committee (TSC)

This will be chaired by Professor Craig Steel, Oxford Health NHS Foundation Trust and the University of Oxford, and members will include Associate Professor Katherine Newman-Taylor, Consultant Clinical Psychologist & Associate Professor of Clinical Psychology, University of Southampton and Beverley Shirkley, Senior Research Associate in Medical Statistics, University of Bristol will undertake the Independent Trial Statistician role. The TSC for this feasibility study will also undertake the role of a Data Monitoring and Ethics Committee (DMEC). We are also approaching a service user representative to have a key voice on the TSC.

The role of the TSC includes:

- Advising the chief investigator on all aspects of the trial
- Provide overall supervision of the trial protocol and statistical analysis
- Monitor trial progress
- Review relevant information from other sources related to the trial
- Review outputs and final reports

The Trial Management group will meet regularly to ensure all elements of the trial are progressing well and working well and everyone within the trial understands them.

# ETHICS and DISSEMINATION

## RESEARCH ETHICS COMMITTEE REVIEW AND REPORTS

We will submit relevant documentation for review to the UK National Health Service Health Research Authority (HRA) and seek approval from a Research Ethics Committee.

This study will be conducted in accordance with the principles of the International Conference on Harmonisation Tripartite Guideline for Good Clinical Practice (ICH GCP). Before the study commences, all required approvals will be obtained and any conditions of approvals will be met.

The trial will have local approval and the sponsor and study will verify this before giving the site the “green light” to commence recruitment.

## PROTOCOL AMENDMENTS

The Chief Investigator will review and approve and changes to the study protocol (except those in relation to urgent safety issues) in regard to any changes in research activity. Any study amendments will be reviewed and approved a Sponsor Representative for review and authorization before being submitted to the HRA Research Ethics Committee and R and I department for review and approval before any participants are entered into the revised study protocol procedures.

## PEER REVIEW

The grant application was submitted to the National Institute for Health Research (NIHR)’s Research for Patient Benefit (RfPB) response mode funding scheme, under a specific “rapid call” for mental health projects in Northern England. It was peer reviewed and the committee gave feedback which has been incorporated into the protocol.

## Public Patient Involvement

Our PPI co-applicant/service user consultant Thom and other service users inputs at consultation meetings have led to specific changes to the case series study and the iMAPS-2 grant application, resulting in changes to this protocol.

We are in the process of recruiting an iMAPS-2 service user reference group, chaired by Thom, who will be consulted on all matters relating to data collection, analysis and dissemination. The group will have between 4 – 6 members with 3-4 meetings per year.

## Protocol Compliance

Protocol breaches, non-compliances or deviations are departures from the approved protocol. Prospective, planned deviations or waivers to the protocol are not permitted under the regulations governing clinical trials. For example, it is not acceptable to recruit a participant if they do not meet the eligibility criteria or restrictions specified within the trial protocol. Accidental protocol deviations can happen at any time. They must be documented on the relevant forms and reported to the CI immediately. Deviations from the protocol which are found to frequently recur are not acceptable, will require immediate action and could potentially be classified as a serious breach.

## Notification of Serious Breaches to GCP and/ or the protocol

A “serious breach” is a breach of GCP or the protocol which is likely to effect to a significant degree –

- 1. the safety or physical or mental integrity of the subjects of the
  2. trial; or
  3. the scientific value of the trial/ data reliability or robustness

The CI or Sponsor will notify the relevant REC in writing of any serious breach of the protocol or GCP.

## SERIOUS BREACH OF PROTOCOL

A serious breach is a breach which is likely to effect to a significant degree:

(a) the safety or physical or mental wellbeing of the participants in the study; or

(b) the scientific value of the study.

If a possible serious breach is reported by the Chief Investigator or research team, the Sponsor will be notified within 24 hours. The Sponsor will assess the implications of the breach on the scientific value of the study, to determine if the incident is a serious breach or not and report to the relevant research ethics committee.

Protocol deviations, non-compliances, or breaches are departures from the approved protocol. Prospective, planned deviations or waivers to the protocol are not allowed under the Clinical Trials Regulations and must not be used e.g. it is not acceptable to enrol a subject if they do not meet the eligibility criteria or restrictions specified in the trial protocol. Accidental protocol deviations can happen at any time. They must be adequately documented on the relevant forms and reported to the Chief Investigator immediately. Deviations from the protocol which are found to frequently recur are not acceptable, will require immediate action and could potentially be classified as a serious breach.

## INSPECTION OF RECORDS

The Sponsor, Health Research Authority NHS Research Ethics Committee and any relevant regulatory inspection will be permitted to undertake monitoring or audit as required. The investigators and research team will give access to study records and source documentation when an audit or monitoring is initiated. In the event of regulatory inspection, the researchers and investigators will agree to allow inspectors direct access to all study records and source documentation.

### Data Protection

Please see appendix for a copy of the study data management plan.

## INSURANCE AND INDEMNITY

The protocol has been written by the Chief Investigator and researchers employed by the University and collaborators. The NHS Trust (Pennine Care NHS FT) has insurance in place (including no fault compensation) for negligent harm caused by poor protocol design by the Chief Investigator and researchers employed by the Trust.

Sites taking part in the study will be liable for clinical negligence and other negligent harm to individuals taking part in the study and covered by the duty of care owed to them by the site concerned. The Sponsor requires individual sites taking part to arrange their own insurance or indemnity in respect of these liabilities.

All NHS sites including Pennine Care NHS Foundation Trust are part of the UK’s National Health Service and have NHS Indemnity.

## DISSEMINATION POLICY

The key output will be an imagery focused therapy for people with psychosis, feasible to deliver in NHS settings, ready for testing in a definitive clinical trial.

We plan to disseminate our findings via peer review publications, presentations, reports on websites, etc. The findings will be widely disseminated to all relevant stakeholders, including patients, mental health staff, NHS managers, service commissioners and the general public. All participants will be asked if they want to receive details of the study findings and if they consent a lay summary will be emailed or posted to them.

All Publications will acknowledge the contribution of the NIHR, NHS and the host NHS Trust.

## AUTHORSHIP POLICY

The data ownership is with the study team. The Chief Investigator will be the first author on the main publications arising from the trial. Authorship according to the International Committee of Medical Journal Editors recommends that authorship be based on the following 4 criteria:

- Substantial contributions to the conception or design of the work; or the acquisition, analysis, or interpretation of data for the work; AND
- Drafting the work or revising it critically for important intellectual content; AND
- Final approval of the version to be published; AND
- Agreement to be accountable for all aspects of the work in ensuring that questions related to the accuracy or integrity of any part of the work are appropriately investigated and resolved.

We are keen to ensure that all research and clinical staff involved have the opportunity to meet the criteria above and ensure their contributions to the project are appropriately recognized. We plan to develop a full authorship plan and agree this as far as possible in advance, assuming satisfactory completion of pre-planned tasks and roles.

### Study suspension or discontinuation

In the event there are a number of therapy attributed adverse events or serious adverse events, the research would be paused. Any serious adverse events and decisions regarding these were attributable or non-attributable will be passed to the independent TSC incorporating a DMEC function for review, who would have responsibility for reviewing those decisions. They would advise on threshold for discontinuation and issuing advice to suspend or discontinue. The research team would act on this advice in consultation and discussion with the Sponsor (Pennine Care NHS FT) and the Research Ethics Committee (See Section 9 for more details).

# REFERENCES

Addington, D., Addington, J., & Maticka-Tyndale, E. (1993). Assessing depression in schizophrenia: the Calgary Depression Scale. *The British Journal of Psychiatry*, *163*(S22), 39-44.

Addington, J., Cadenhead, K. S., Cannon, T. D., Cornblatt, B., McGlashan, T. H., Perkins, D. O., Seidman, L. J., Tsuang, M., Walker, E. F., Woods, S. W., & Heinssen, R. (2007). North American Prodrome Longitudinal Study: A Collaborative Multisite Approach to Prodromal Schizophrenia Research. *Schizophrenia Bulletin*, *33*(3), 665-672. <https://doi.org/10.1093/schbul/sbl075>

Beck, A. T., Epstein, N., Brown, G., & Steer, R. A. (1988). An inventory for measuring clinical anxiety: Psychometric properties. *Journal of Consulting and Clinical Psychology*, *56*, 893–897.

Blackburn, I.-M., James, I. A., Milne, D. L., Baker, C., Standart, S., Garland, A., & Reichelt, F. K. (2001). The revised cognitive therapy scale (CTS-R): psychometric properties. *Behavioural and Cognitive Psychotherapy*, *29*(4), 431-446.

Bowe, S. (2017, 10th Feb, 2017). *Delivering CBT on the FOCUS trial. Can the milestones in the CBT treatment protocol be met?* Translating Research about Psychosis into Clinical Practice, Manchester, UK.

Braun, V., & Clarke, V. (2006). Using Thematic Analysis in Psychology. *Qualitative Research In Psychology 3*(2), 77–101.

Carr, S., Hardy, A., & Fornells-Ambrojo, M. (2018). The Trauma and Life Events (TALE) checklist: development of a tool for improving routine screening in people with psychosis. *European journal of psychotraumatology*, *9*(1), 1512265.

Cloitre, M., Shevlin, M., Brewin, C. R., Bisson, J. I., Roberts, N. P., Maercker, A., Karatzias, T., & Hyland, P. (2018). The International Trauma Questionnaire: Development of a self‐report measure of ICD‐11 PTSD and complex PTSD. *Acta Psychiatrica Scandinavica*, *138*(6), 536-546.

Dazzi, T., Gribble, R., Wessely, S., & Fear, N. T. (2014). Does asking about suicide and related behaviours induce suicidal ideation? What is the evidence? *Psychological medicine*, *44*(16), 3361-3363.

Fowler, D., Freeman, D., Smith, B., Kuipers, E., Bebbington, P., & Bashforth, H. (2006). The Brief Core Schema Scales (BCSS): psychometric properties and associations with paranoia and grandiosity in non-clinical and psychosis samples. *36*, 749-759. <https://doi.org/10.1017/S0033291706007355>

Fowler, D., Freeman, D., Smith, B., Kuipers, E., Bebbington, P., Bashforth, H., Coker, S., Hodgekins, J., Gracie, A., Dunn, G., & Garety, P. (2006). The Brief Core Schema Scales (BCSS): Psychometric properties and associations with paranoia and grandiosity in non-clinical and psychosis samples. *Psychological Medicine*, *36*(6), 749-759. <https://doi.org/https://doi.org/10.1017/S0033291706007355>

Fowler, D., Garety, P., & Kuipers, E. (1995). *Cognitive behaviour therapy for psychosis: Theory and practice*. Wiley

Haddock, G., Devane, S., Bradshaw, T., McGovern, J., Tarrier, N., Kinderman, P., Baguley, I., Lancashire, S., & Harris, N. (2001). An investigation into the psychometric properties of the Cognitive Therapy Scale for Psychosis (CTS-Psy). *Behavioural and Cognitive Psychotherapy*, *29*(2), 221-233.

Haddock, G., McCarron, J., Tarrier, N., & Faragher, E. B. (1999). Scales to measure dimensions of hallucinations and delusions: the psychotic symptom rating scales (PSYRATS). *Psychological Medicine*, *29*(4), 879-889. <https://doi.org/http://dx.doi.org/10.1017/S0033291799008661>

Hatcher, R. L., & Gillaspy, J. A. (2006). Development and validation of a revised short version of the working alliance inventory. *Psychotherapy Research*, *16*(1), 12-25. <https://doi.org/10.1080/10503300500352500>

Holmes, E. A., Bonsall, M. B., Hales, S. A., Mitchell, H., Renner, F., Blackwell, S. E., Watson, P., Goodwin, G. M., & Di Simplicio, M. (2016). Applications of time-series analysis to mood fluctuations in bipolar disorder to promote treatment innovation: a case series [Original Article]. *Transl Psychiatry*, *6*, e720. <https://doi.org/10.1038/tp.2015.207>

Hutton, P. (2013). *Adverse Effects of Psychotherapy (AEP) Measure*. University of Edinburgh.

Ison, R., Medoro, L., Keen, N., & Kuipers, E. (2014). The Use of Rescripting Imagery for People with Psychosis Who Hear Voices. *Behavioural and Cognitive Psychotherapy*, *42*(02), 129-142. <https://doi.org/10.1017/S135246581300057X>

Janssen, M. F., Pickard, A. S., Golicki, D., Gudex, C., Niewada, M., & Scalone, L. (2013). Measurement properties of the EQ-5D-5L compared to the EQ-5D-3L across eight patient groups: a multi-country study. *22*, 1717-1727. <https://doi.org/10.1007/s11136-012-0322-4>

Jauhar, S., McKenna, P. J., Radua, J., Fung, E., Salvador, R., & Laws, K. R. (2014). Cognitive-behavioural therapy for the symptoms of schizophrenia: systematic review and meta-analysis with examination of potential bias. *The British Journal of Psychiatry*, *204*(1), 20-29. <https://doi.org/10.1192/bjp.bp.112.116285>

Kay, S. R., Fiszbein, A., & Opler, L. A. (1987). The Positive and Negative Syndrome Scale (PANSS) for Schizophrenia. *Schizophrenia Bulletin*, *13*(2), 261-276. <https://doi.org/10.1093/schbul/13.2.261>

Keen, N., Hunter, E. C. M., & Peters, E. (2017). Integrated Trauma-Focused Cognitive-Behavioural Therapy for Post-traumatic Stress and Psychotic Symptoms: A Case-Series Study Using Imaginal Reprocessing Strategies [Original Research]. *Frontiers in Psychiatry*, *8*(92). <https://doi.org/10.3389/fpsyt.2017.00092>

Keetharuth, A. D., Brazier, J., Connell, J., Bjorner, J. B., Carlton, J., Buck, E. T., Ricketts, T., McKendrick, K., Browne, J., & Croudace, T. (2018). Recovering Quality of Life (ReQoL): a new generic self-reported outcome measure for use with people experiencing mental health difficulties. *The British Journal of Psychiatry*, *212*(1), 42-49.

Kingdon, D. G., & Turkington, D. (2005). *Cognitive-behavioral therapy of schizophrenia: 2nd Edition*. Guilford Press.

Lancaster, G. A., Dodd, S., & Williamson, P. R. (2004). Design and analysis of pilot studies: recommendations for good practice. *Journal of Evaluation in Clinical Practice*, *10*(2), 307-312.

Law, H., Neil, S. T., Dunn, G., & Morrison, A. P. (2014). Psychometric properties of the Questionnaire about the Process of Recovery (QPR). *Schizophrenia Research*, *156*(2–3), 184-189. <https://doi.org/http://dx.doi.org/10.1016/j.schres.2014.04.011>

Leucht, S., Leucht, C., Huhn, M., Chaimani, A., Mavridis, D., & Helfer, B. (2017). Sixty years of placebo-controlled antipsychotic drug trials in acute schizophrenia: systematic review, bayesian meta-analysis, and meta-regression of efficacy predictors. *174*, 927-942. <https://doi.org/10.1176/appi.ajp.2017.16121358>

Morosini, P. L., Magliano, L., Brambilla, L., Ugolini, S., & Pioli, R. (2000). Development, reliability and acceptability of a new version of the DSM-IV Social and Occupational Functioning Assessment Scale (SOFAS) to assess routine social functioning. *101*, 323-329.

Morrison, A., Beck, A., Glentworth, D., Dunn, H., Reid, G., Larkin, W., & Williams, S. (2002). Imagery and psychotic symptoms: A preliminary investigation. *Behaviour Research and Therapy*, *40*(9), 1053-1062.

Morrison, A., Renton, J., Dunn, H., Williams, S., & Bentall, R. (Eds.). (2004). *Cognitive therapy for psychosis: A formulation-based approach*. Routledge.

Morrison, A. P., Pyle, M., Gumley, A., Schwannauer, M., Turkington, D., & MacLennan, G. (2018). Cognitive behavioural therapy in clozapine-resistant schizophrenia (FOCUS): an assessor-blinded, randomised controlled trial. *5*, 633-643. <https://doi.org/10.1016/S2215-0366(18)30184-6>

National_Institute_for_Health_and_Care_Excellence. (2014). *NICE guidelines CG178 - Psychosis and schizophrenia in adults: Treatment and management.* London: National Institute for Health and Care Excellence.

Paulik, G., Steel, C., & Arntz, A. (2019). Imagery rescripting for the treatment of trauma in voice hearers: a case series. *Behavioural and Cognitive Psychotherapy*, *47*(6), 709-725. <https://doi.org/10.1017/S1352465819000237>

Power, M. J. (2006). The structure of emotion: An empirical comparison of six models. *Cognition & Emotion*, *20*(5), 694-713.

Reisberg, D., Pearson, D. G., & Kosslyn, S. M. (2003). Intuitions and introspections about imagery: The role of imagery experience in shaping an investigator's theoretical views. *Applied Cognitive Psychology*, *17*(2), 147-160. <https://doi.org/https://doi.org/10.1002/acp.858>

Schulze, K., Freeman, D., Green, C., & Kuipers, E. (2013). Intrusive mental imagery in patients with persecutory delusions. *Behaviour research and therapy*, *51*(1), 7-14. <https://doi.org/https://doi.org/10.1016/j.brat.2012.10.002>

Sekhon, M., Cartwright, M., & Francis, J. J. (2017). Acceptability of healthcare interventions: an overview of reviews and development of a theoretical framework. *BMC health services research*, *17*(1), 1-13.

Sheaves, B., Onwumere, J., Keen, N., & Kuipers, E. (2015). Treating your worst nightmare: a case-series of imagery rehearsal therapy for nightmares in individuals experiencing psychotic symptoms. *The Cognitive Behaviour Therapist*, *8*, e27.

Shields, G. E., Wells, A., Doherty, P., Reeves, D., Capobianco, L., Heagerty, A., Buck, D., & Davies, L. M. (2020). Protocol for the economic evaluation of metacognitive therapy for cardiac rehabilitation participants with symptoms of anxiety and/or depression. *BMJ Open*, *10*(9), e035552. <https://doi.org/10.1136/bmjopen-2019-035552>

Tanskanen, A., Tiihonen, J., & Taipale, H. (2018). Mortality in schizophrenia: 30‐year nationwide follow‐up study. *Acta Psychiatrica Scandinavica*, *138*(6), 492-499.

Taylor, C., Bee, P. E., Kelly, J., Emsley, R., & Haddock, G. (2020). iMAgery focused psychological therapy for persecutory delusions in PSychosis (iMAPS): a multiple baseline experimental case series. *Behavioural and Cognitive Psychotherapy*, *48*, 530-545. <https://doi.org/doi:10.1017/S1352465820000168>

Taylor, C. D. J., Bee, P. E., Emsley, R., Taylor, P., Ibbs, L., Baker, C., & Haddock, G. (2022; Under Review). Detailed examination of the role of negative and positive schema in predicting psychotic symptoms in psychosis: An experience sampling study. *Manuscript Submitted for Publication*.

Taylor, C. D. J., Bee, P. E., Kelly, J., & Haddock, G. (2019). iMAgery focused therapy for persecutory delusions in PSychosis (iMAPS): A novel treatment approach. *Cognitive and Behavioural Practice*, *25*, 575-588. <https://doi.org/https://doi.org/10.1016/j.cbpra.2018.10.002>

Taylor, C. D. J., Haddock, G., Speer, S., & Bee, P. E. (2020). Characterizing core beliefs in psychosis: a qualitative study. *Behavioural and Cognitive Psychotherapy*, *48*, 67-81. <https://doi.org/doi:10.1017/S1352465819000274>

Taylor, C. D. J., & Harper, S. F. (2017). Early maladaptive schema, social functioning and distress in psychosis: A preliminary investigation. *Clinical Psychologist*, *21*, 135-142. <https://doi.org/10.1111/cp.12082>

Tennant, R., Hiller, L., Fishwick, R., Platt, S., Joseph, S., Weich, S., Parkinson, J., Secker, J., & Stewart-Brown, S. (2007). The Warwick-Edinburgh Mental Well-being Scale (WEMWBS): development and UK validation. *Health and Quality of Life Outcomes*, *5*(1), 63. <http://www.hqlo.com/content/5/1/63>

Varese, F., Smeets, F., Drukker, M., Lieverse, R., Lataster, T., Viechtbauer, W., Read, J., van Os, J., & Bentall, R. P. (2012). Childhood adversities increase the risk of psychosis: a meta-analysis of patient-control, prospective-and cross-sectional cohort studies. *Schizophrenia Bulletin*, *38*(4), 661-671. <https://doi.org/http://dx.doi.org/10.1093/schbul/sbs050>

Westman, J., Eriksson, S., Gissler, M., Hällgren, J., Prieto, M., Bobo, W., Frye, M., Erlinge, D., Alfredsson, L., & Ösby, U. (2018). Increased cardiovascular mortality in people with schizophrenia: a 24-year national register study. *Epidemiology and Psychiatric Sciences*, *27*(5), 519-527.

## APPENDICES:

## APPENDIX 1: Proposed CONSORT Flow Diagram

Service Users with Psychosis in PCFT NHS Services (Five boroughs: Bury, Rochdale, Oldham, Stockport, Tameside)

300 patients per borough x 5 boroughs

(N = 1,500)

Screening

Follow-Up

**16 weeks post randomisation assessments**

Lost to follow-up (give reasons) (n = ? )

Discontinued intervention (give reasons) (n =?) )

Allocated to iMAPS + TAU (n = 30)

♦ Received allocated intervention (n= ? )

♦ Did not receive allocated intervention (give reasons) (n = ? )

Allocated to TAU (n = 15 )

♦ Received allocated intervention (n = ? )

♦ Did not receive allocated intervention (give reasons) (n = ? )

Randomized (n = 45 )

Excluded (n= ? )

♦  Not meeting inclusion criteria (n= ? )

♦  Declined to participate (n= ? )

♦  Other reasons (n= ? )

Assessed for eligibility (n = 240) )

Eligibility Assessment

Informed Consent

Analysed (n = ? )
♦ Excluded from analysis (give reasons) (n= ?)

Analysed (n= )
♦ Excluded from analysis (give reasons) (n = ? )

Excluded (n= ? )

♦  Did not have a current or recent persecutory delusion (at least 3 on PANSS P1)

♦ Did not report at least one negative belief scoring >1 on BCSS

♦  Declined to participate (n= ? )

♦  Other reasons (n= ? )

Enrollment

**28 weeks post randomisation assessments**

Lost to follow-up (give reasons) (n = ? )

Discontinued intervention (give reasons) (n =?) )

**28 weeks post randomisation assessments**

Lost to follow-up (give reasons) (n = ? )

Discontinued intervention (give reasons) (n =?) )

**16 weeks post randomisation assessments**

Lost to follow-up (give reasons) (n = ? )

Discontinued intervention (give reasons) (n =?) )

Analysis

Allocation

2:1 Randomisation

Figure 1: Proposed CONSORT Flow Diagram

iMAPS-2 Statistical and Health Economics Analysis Plan (SHEAP)


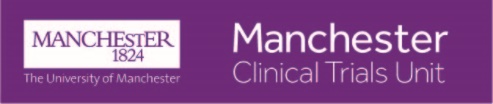


iMAPS-2 Statistical and Health Economics Analysis Plan (SHEAP)

**Full/ Long title of the Trial:** iMAgery focused therapy for Psychosis (iMAPS-2): A feasibility randomised controlled trial

**Short Study title/ Acronym:** iMAgery focused therapy for Psychosis (iMAPS-2):

Trial registration number(s): ISRCTN81150786

SHEAP version number with dates: v1.0 30^th^ May 2024

Protocol version number and date: 5.0, 27^th^ Oct 2023

SHEAP revisions:

| Protocol version | Updated SAP version number | Section number changed | Description of and reason for change | Date changed | Initials |
| --- | --- | --- | --- | --- | --- |
| 5.0 | 1.0 |  | First issue |  | YS |
|  |  |  |  |  |  |

**Roles and responsibility:** names, affiliations and roles of SAP contributors

Signatures

| Trial Statistician: Yvonne Sylvestre, Research Fellow in Clinical Trials Statistics | | |
| --- | --- | --- |
| Signature: |  | Date: 04/Jun/2024  ddmmmyyyy |
| Name: (please print):  ......Yvonne Sylvestre........................................................ |  |  |
| Lead Statistician: Dr Chris Sutton, Senior Lecturer in Clinical Trials Statistics | | |
| Signature: |  | Date: .03/Jun/2024  ddmmmyyyy |
| Name: (please print):  ................... Dr. Chris Sutton ............................................. |  |  |
| Trial Steering Committee Chair: Professor Craig Steel, Professor of Clinical Psychology and Consultant Clinical Psychologist | | |
| Signature: .................... ............... |  | Date: ..4./.6./.2024....  ddmmmyyyy |
| Name: (please print):  ...................................................................................................... |  |  |
| Chief Investigator: Dr Chris Taylor, Consultant Clinical Psychologist & Honorary Senior Lecturer | | |
| Signature: |  | Date: 30/05/2024 |

| Name: (please print): DR CHRIS TAYLOR |  |  |
| --- | --- | --- |

Contents

[2 abbreviations 5](#_Toc167457104)

[3 Introduction 6](#_Toc167457105)

[3.1 Background and rationale 6](#_Toc167457106)

[3.2 Aim and objectives 6](#_Toc167457107)

[4 Trial Methods 7](#_Toc167457108)

[4.1 Trial design 7](#_Toc167457109)

[4.2 Blinding (masking) 8](#_Toc167457110)

[4.3 Randomisation 8](#_Toc167457111)

[4.4 Sample size 8](#_Toc167457112)

[4.5 Framework 8](#_Toc167457113)

[4.6 Statistical interim analysis and stopping guidance 8](#_Toc167457114)

[Interim Analysis 8](#_Toc167457115)

[Guidelines for stopping the trial early 8](#_Toc167457116)

[4.7 Timing of final analysis 9](#_Toc167457117)

[4.8 Timing of outcome assessments 9](#_Toc167457118)

[5 Statistical Principles 9](#_Toc167457119)

[5.1 Confidence intervals (CI) and level of statistical significance 9](#_Toc167457120)

[5.2 Adherence and protocol deviations 10](#_Toc167457121)

[Adherence 10](#_Toc167457122)

[Protocol deviations 12](#_Toc167457123)

[5.3 Analysis populations 12](#_Toc167457124)

[6 Trial Population 12](#_Toc167457125)

[6.1 Screening data 12](#_Toc167457126)

[6.2 Eligibility 12](#_Toc167457127)

[6.3 Recruitment 13](#_Toc167457128)

[6.4 Withdrawal/follow-up 13](#_Toc167457129)

[6.5 Baseline patient characteristics 13](#_Toc167457130)

[7 Analysis 15](#_Toc167457131)

[7.1 Calculation of clinical scores 15](#_Toc167457132)

[Positive and Negative Syndrome Scale (PANSS) 15](#_Toc167457133)

[Psychotic Symptom Rating Scales (PSYRATS) 16](#_Toc167457134)

[Questionnaire about the Process of Recovery (QPR) 17](#_Toc167457135)

[Mental Imagery in Psychosis Questionnaire/Visual Analogue Scales (MIPQ/VAS) 17](#_Toc167457136)

[Psychosis Imagery Questionnaire (PIQ) 18](#_Toc167457137)

[Brief Core Schema Scale (BCSS) 18](#_Toc167457138)

[International Trauma Questionnaire (ITQ) 18](#_Toc167457139)

[Basic Emotions Scale (BES) 19](#_Toc167457140)

[Beck Anxiety Inventory (BAI) 20](#_Toc167457141)

[Calgary Depression Scale (CDS) 20](#_Toc167457142)

[Warwick Edinburgh Mental Well Being Scale (WEMWBS) 20](#_Toc167457143)

[The Personal and Social Performance Scale (PSP) 20](#_Toc167457144)

[Working Alliance Inventory Short form (WAI-SR) 21](#_Toc167457145)

[Clinical Global Impression- Improvement (CGI-I) 22](#_Toc167457146)

[Clinical Global Impression - Severity (CGI-S) Version 22](#_Toc167457147)

[7.2 Analysis methods 22](#_Toc167457148)

[Sensitivity analyses 27](#_Toc167457149)

[7.3 Subgroup analyses 27](#_Toc167457150)

[7.4 Missing data 28](#_Toc167457151)

[7.5 Additional analyses 28](#_Toc167457152)

[7.6 Harms 29](#_Toc167457153)

[7.7 Health economic analysis 30](#_Toc167457154)

[Health status (EQ-5D) and Quality-Adjusted Life-Years (QALYs) 30](#_Toc167457155)

[Health and social care use 30](#_Toc167457156)

[Intervention costs 31](#_Toc167457157)

[Exploratory cost-effectiveness analysis 31](#_Toc167457158)

[7.8 Statistical software 32](#_Toc167457159)

[8 References 32](#_Toc167457160)

[9 APPENDIX 36](#_Toc167457161)

# abbreviations

| AE | Adverse Event |
| --- | --- |
| CBT | Cognitive Behavioural Therapy |
| CI | Confidence Interval |
| CMHT | Community Mental Health Team |
| CONSORT | Consolidated Standards Of Reporting Trials |
| EI | Early Intervention Psychosis Team Health Team |
| ITT | Intention-to-Treat |
| MID | Minimally Important Difference |
| NICE | National Institute for Health and Care Excellence |
| MLE | Maximum Likelihood Estimator |
| RCT | Randomised Controlled Trial |
| SAE | Serious Adverse Event |
| SD | Standard Deviation |
| SHEAP | Statistical and Health Economics Analysis Plan |
| TAU | Treatment as Usual |

# Introduction

## Background and rationale

Schizophrenia is a severe mental health condition, for example, where individuals hear distressing voices other people cannot hear (e.g. auditory hallucinations) and/or have distressing unusual beliefs that others do not share (delusions). They also report negative core beliefs (e.g., “I am vulnerable; I am a failure; others are hostile”) and unwanted intrusive mental images (that are in the “mind’s eye” and other senses) which may be maintaining factors for psychotic symptoms.

One of the best interventions for psychosis is Cognitive Behavioural Therapy (CBT), which is recommended by the UK National Institute for Health and Care Excellence (NICE). Sadly, the first generation of CBT for Psychosis (adapted from CBT for emotional disorders) has a small effect size, and there is a need to refine and improve it. Imagery approaches are almost completely absent from multiple CBT for Psychosis therapy manuals. Empirical studies consistently demonstrate that imagery has a more powerful impact on emotion than verbal cognition. Therefore, we anticipate using an imagery focused approach to target images and schemas will result in a reduction in psychotic symptoms.

We wish to undertake a feasibility randomised controlled trial (RCT) of an imagery focused therapy called iMAPS-2, which targets negative images and negative core beliefs (schemas). Our research aims to improve the current treatments for people with psychosis. We wish to explore a psychological therapy (i.e. iMAPS-2) where the therapist and client specifically work with distressing “mental imagery” (e.g., “pictures in the mind’s eye, sounds in the mind’s ear”), and negative beliefs, which are often reported but rarely treated. Moreover, the project will tell us if we may be able to run a definitive randomised controlled clinical trial. Additionally we will explore proof-of-efficacy on health outcome measures (i.e., Positive and Negative Syndrome Scale (PANSS) Total score, Psychotic Symptom Rating Scales (PSYRATS), Questionnaire about the Process of Recovery (QPR) and Brief Core Schema Scale (BCSS)), but we will also be looking at proof-of-concept (Basic Emotions Scale (BES), Beck Anxiety Inventory (BAI), Calgary Depression Scale (CDS), Warwick Edinburgh Mental Well Being Scale (WEMWBS) and the Personal and Social Performance Scale (PSP)).

## Aim and objectives

The study aims to assess whether it is feasible to conduct a RCT to examine the clinical and cost effectiveness of an imagery focused psychological therapy in psychosis.

Research Questions:

1. What number and percentage of eligible patients/service users consent to the trial (recruitment)?

2. What is the level of engagement with adherence to the iMAPS-2 intervention (therapy sessions attendance measures; therapist fidelity)?

3. What completion and data quality rates can be achieved (data completion and retention of participants)?

4. What estimates of effect sizes (if any) are present (acknowledging this is a feasibility trial)?

5. What are service users’ views regarding:

i) acceptability of participating in the trial

ii) the outcomes measures collected, including their acceptability and the ranking of the potential primary outcome measures for the definitive trial, and

iii) acceptability of receiving iMAPS-2 therapy (including adherence to intervention protocol)?

**Qualitative component only: not covered in this SHEAP.**

6. What is the estimated sample size for a fully powered trial to evaluate the effectiveness of iMAPS-2 (relative to usual care)?

7. What is the range of services used by participants and which are likely to be key cost drivers to consider for the main trial?

8. What is the range of health benefits and are they covered by the EQ-5L health status questionnaire?

# Trial Methods

## Trial design

This is a feasibility assessor-blind RCT comparing treatment as usual (TAU) plus iMAPS-2 therapy, versus TAU, in a 2:1 allocation ratio.

Participants allocated to the intervention arm received 12 sessions of individualised iMAPS-2 psychological therapy.

iMAPS-2 will also look at the feasibility of establishing the optimum way of evaluating cost-effectiveness.

In addition, iMAPS-2 has a nested qualitative study design of participants’ experiences of iMAPS-2 intervention and trial participation, to identify themes relating to the acceptability of this therapy and inform parameters of the definitive trial including therapy protocol, outcome measures, recruitment and retention. However, the qualitative analysis will not be performed by the Trial Statistician or Health Economist and therefore is not described in this document.

## Blinding (masking)

Researchers undertaking assessments were blinded. Any instances of unintentional unblinding were recorded and reported.

The Trial Statistician will remain blinded to treatment allocation until after the SHEAP has been sign off and the analysis of unblinded data commences. It was not possible to blind the participants to allocation group.

## Randomisation

Participants were allocated in a 2:1 ratio to iMAPS-2 intervention plus usual care or usual care alone, using random permuted blocks stratifying by service user/patient under the care of a Community Mental Health Team (CMHT) or an Early Intervention Psychosis Team (EI).

## Sample size

Recruiting 45 participants will allow to estimate the retention rate at end of therapy with a 95% (exact binomial) confidence interval (CI), with width no greater than 25%, assuming retention is at least 80%.  It will also suffice for the estimation of the SD, although the sample size (expected minimum 36 participants with outcome data) is towards the lower end of the sample size recommended [1] [2]

## Framework

This is a feasibility trial, within a superiority framework.

## Statistical interim analysis and stopping guidance

### Interim Analysis

No interim analysis will be performed.

### Guidelines for stopping the trial early

There are no statistical reasons specified for stopping the trial early. The Chair of the TSC reviewed all serious adverse events. The trial would be stopped early to prevent harm, if there were an undue number of related SAE. The TSC also considered any relevant safety data, any recommendations for early closure of the study on this basis would had been based on their expert opinion, there were no formal statistical criteria specified for stopping the trial early.

## Timing of final analysis

All analyses will be performed at the end of the follow-up period, once all data are either collected or data from any participants with outstanding outcome data is declared missing as agreed by the Chief Investigator and Statisticians.

## Timing of outcome assessments

Outcome assessments will be performed at baseline, 16 weeks (end-of-therapy assessment) and 28 weeks (follow-up assessment) although there is no strict window for outcome assessments.

# Statistical Principles

## Confidence intervals (CI) and level of statistical significance

As this is a feasibility study, the emphasis will be on producing relevant data summaries.. For consideration of potential proof-of-efficacy investigations (i.e., Positive and Negative Syndrome Scale (PANSS) Total score, Psychotic Symptom Rating Scales (PSYRATS), Questionnaire about the Process of Recovery (QPR) and Brief Core Schema Scale (BCSS)), two-sided 100*(1-α)% confidence intervals with α ranging from 0.05 to 0.25 (in steps of 0.05) will be computed [3], thus reporting adjusted point estimates and confidence intervals (CIs), ranging from 75% to 95% confidence, for the effect obtained from the analyses described in section 7.2. This approach is based on a minimally-important difference (MID) between trial arms and is therefore more appropriate than formal hypothesis testing when a study is underpowered.

The MID for the PANSS total score can range between 4 and 21 points [4] [5]. Due to lack of literature to select appropriate MID for the PANSS and the rest primary outcome candidates, we define a promising effect to be around a standard effect size of 0.3 (or more) [ref]. In addition, we will consider an absolute change of 5 points in the Total PANSS scores as a promising effect.

Two-sided 95% confidence intervals will be presented for the estimates of the treatment effects of all other continuous outcomes. No formal testing will be performed and, as a result, p-values will not be presented.

## Adherence and protocol deviations

### Adherence

Adherence to the intervention relies upon both therapist and participant factors.

a) Participant adherence

Participant adherence to the iMAPS-2 intervention will be measured by the number of therapy sessions attended by the participants allocated to the intervention arm. An individual will be classed as having attended a session if they attended a part or the whole of a session (defined as a minimum duration of 15 minutes). We will report the mean (SD) number of sessions attended or median (IQR), as appropriate. We will also summarise the number (%) of adherent participants with a 95% exact (binomial) confidence interval (for the %). If a participant attends 5 or more of the 12 sessions [4], then they will be deemed adherent.

b) Therapist fidelity

Therapist fidelity will be assessed by:

i) Rating a sample of audio recordings of therapy sess ions using the Cognitive Therapy Rating Scale Revised (CTS-R) or the Cognitive Therapy Rating Scale for Psychosis (CTS-Psy). Scale scores will be summarised as mean (SD) if they are (approximately) symmetrically distributed or median (IQR) if they are asymmetrically distributed.

ii) Therapists who are part of the research team (approximately 4 therapists) will complete the iMAPS fidelity scale after each therapy session to record the contents of each session in terms of: agenda targets; between-session activities/home tasks; the techniques used. Items within each domain of the fidelity scale will be summarised as follows:

**Agenda targets**

1. Is there a target Image? (yes/no)
2. Distress re image (0-100)
3. Impact on Functioning (0-100)
4. Is there an Agenda? (at least 1 item included)

We will obtain the frequency (%) of all sessions where both a target image is present and there is an agenda with at least 1 item included. Distress image and Impact on functioning will each be summarised by mean (SD) or median (IQR) as appropriate.

**Between-Session Activities(BSA)/Home tasks**

1. Last session BSA reviewed as part of agenda? YES/NO
2. Has it been COMPLETED? YES/NO
3. Current session BSA agreed (collaboratively)? YES/NO
4. Last session therapist BSA reviewed as part of agenda? YES/NO
5. Has it been COMPLETED? YES/NO
6. Current Session Therapist BSA set? YES/NO

We will obtain frequencies (%) of all sessions where:

- The previous session home task was reviewed
- The previous session home task was completed
- A new session home task was set

And report them separately for the client (items 1 to 3) and therapist (items 4 to 6).

**Technique sessions (Yes, took place in session or NO, did not)**

1. Assessment and Engagement
2. Imagery Problem List
3. iMAPS Psychological Formulation (This could be with either the maintenance formulation, longitudinal formulation or both)
4. Psychoeducation regarding power of imagery
5. Normalising the image
6. Safe Place Image
7. Image Suppression
8. Behavioural Experiments
9. Upsetting Memories – Transformation
10. Upsetting Memories – Running image past the worst point
11. Upsetting memories – updating aspects of the image
12. Emotional bridge to past (diagnostic imagery exercise)
13. Imagery rescripting – past events
14. Imagery rescripting – flashforwards
15. Imagery rescripting – discussion of negative beliefs re self and others, schemas
16. Working with night-time imagery - Updating aspects of the image, rescripting new endings
17. Creating Positive Imagery – generating positive images

We will obtain the frequency (%) of all sessions by technique used.

### Protocol deviations

We will present a listing of all the protocol deviations occurred during the trial by treatment group with details of the type of deviation recorded. No formal statistical analysis will be undertaken.

## Analysis populations

Feasibility analyses will include all the consented participants to estimate the proportion of eligible consented participants and eligible participants randomised. To summarise the number of iMAPS-2 therapy sessions attended, we shall include all the participants allocated to the intervention group. The proportion of adherent participants (attending at least 3 sessions of the iMAPS-2 therapy) will include all the participants allocated to the intervention group.

Analyses of efficacy will be by intention-to-treat (ITT), where all randomised participants for whom outcomes are available will be analysed in their allocated group regardless of their adherence to therapy.

The safety population will also follow the ITT principle and it will comprise all randomised participants.

# Trial Population

## Screening data

We will produce a CONSORT flow diagram [5]. The upper section of the CONSORT flow chart will include;

- Number of patients referred to the study.
- Number of patients screened.
- Number of potentially eligible patients contacted
- Number of patients recruited (i.e., consented)
- Number of participants recruited but not eligible, along with reasons for exclusions.
- Number of eligible participants.
- Number of eligible participants not recruited, along with reasons for non-recruitment.

## Eligibility

The number of non-eligible participants excluded due to not meeting the inclusion/exclusion criteria will be reported in the CONSORT flow chart as stated in section 6.1.

## Recruitment

The lower section of the CONSORT diagram [5] will be used to describe the course of participants through the trial. This will include the:

- Number of participants recruited
- Number of participants recruited but not randomised, included reasons for non-randomisation.
- Number of participants randomised.
- Number of participants allocated to each arm receiving their allocated intervention, and, for the intervention arm, the average number of sessions received.
- Number of participants not fully completing the trial, along with reasons for withdrawal from treatment, withdrawal from follow-up and loss to follow-up (without formal withdrawal from follow-up).
- Number of participants continuing through the trial and,
- Number participants included in the analyses.

We will present overall monthly figures of participants randomised, that will also be tabulated by randomised group.

## Withdrawal/follow-up

We will produce summaries by randomised group of the:

- Number (%) of participants withdrawing from the allocated intervention, along with reasons (if available).
- Number (%) of participants withdrawing fully from the trial (including all follow-up), along with reasons (if available).
- Number (%) of participants lost to follow-up (excluding those who formally withdrew from the trial).

## Baseline patient characteristics

We will summarise the following demographic characteristics both overall and by randomised group:

- Gender (Male/Female/Non-binary/Other)
- Age (years)
- Chart Diagnosis (ICD-11) Schizophrenia, Schizoaffective disorder, delusional disorder, Schizophreniform disorder, Unspecified (non-affective) psychosis – FEP, other.
- ICD-11 Code
- Illness severity as measured by the positive and negative syndrome scale (PANSS), (Mildly ill/Moderately ill/ Markedly ill/ Severely ill)
- Highest education level (Primary school/secondary school/Further education (e.g. College)/Higher education (e.g. University)
- Employment status (Full Time/Part Time/Retired/Voluntary/Student/ Home duties/ Unemployed)
- Marital status (Single/Married/Living with partner/Civil Partnership/Separated/ Divorced/Widowed)
- Living arrangements. Who does the person live with? (Spouse/Partner only, Spouse/Partner plus children, Spouse/Partner plus other/s (not children), Alone, Children only, Parent/s only, Friend/s only, Supported accommodation/hostel, Other)
- Index of Multiple Deprivation (IMD) Decile (derived from Postcode) (adjacent categories will be merged to form three broader categories: 1-3; 4-7; 8-10)
- Ethnicity (Bangladeshi/Indian/Pakistani/Any other Asian background/African/Caribbean/ Any other Black background/ White & Asian/White & Black African/ White & Black Caribbean/ Any other mixed background/ British/ Irish/Any other White background/ Chinese/Any other ethnic group/ Prefer not to answer)
- Treatments received (i.e. past therapy) and current treatment.
- Service type (Early Intervention Psychosis Team, CMHT, Inpatient)
- Inpatient/Outpatient status and legal status will also be reported
- BAI-rated Anxiety (minimal anxiety (0-7), mild anxiety (8-15), moderate anxiety (16-25), severe anxiety (26-63),).
- CDSS rated depression (minimal or absent (0-6), possible major depressive episode(7-27))

Summary measures for the baseline characteristics of each group and overall will be presented as mean and standard deviation, and median and interquartile range (IQR) for continuous variables, and frequency and percentage for categorical variables.

The categories for some of the variables such as ‘Living arrangements’ or ‘ethnicity’ might need to be merged at the time of analysis given the large number of categories and relatively small sample size.

The presence of traumatic events in each randomised group, as measured by the Trauma and Life Events (TALE) Checklist, will be summarised by frequency and percentage and tabulated by:

- Trauma type (item number): war exposure (1) / attachment-related (2 to 4) / witnessed verbal or physical abuse at home (9) / any interpersonal [bullying and discrimination (5&6), sexual abuse (13&14), emotional abuse (7) physical abuse (8&10), emotional neglect (11), physical neglect (12) ] / psychosis-related (15 to 17) / criminal justice (18) / non-interpersonal (19) / other trauma (20).
- Multiple exposure: Repeated events (at least 1 item answered ‘yes’ to ‘more than once’) / Multiple trauma types (more than 1 type of events reported)
- Trauma timing: Child (any time <age 16 years) / Adult (any time >age 16 years) / Both (any time <age 16 years AND > age 16 years).

The perceived impact and number of trauma types will be summarised by mean and standard deviation or median and interquartile range (IQR), as appropriate.

# Analysis

The results of the analyses will adhere to the CONSORT 2010 guidelines and its extension for reporting pilot and feasibility studies [5] [6].

## Calculation of clinical scores

This section describes the scoring and item non-response rules for each tool.

In the absence of tool-specific guidance on handling item non-response, we will seek to impute missing items to complete the tools. We will use a pragmatic approach where if no more than 35% of the items within a (sub)scale are missing, they will be imputed using the mean for all the completed items to provide a valid score for the (sub)scale total, otherwise the (sub)scale will be deemed missing. Total scores will only be available if there are valid (i.e. non-missing) scores for all subscales.

A 35% cut off will allow the scales with a small number of items to have 1 item missing, and the scales with larger number of items a relatively small % of missing items without biasing the results. In psychological outcomes, a range between 20% and 50% is considered acceptable.

***Psychosis Measures***

### Positive and Negative Syndrome Scale (PANSS)

The PANSS is a 30-item therapist-rated scale used to evaluate the symptoms of schizophrenia. All 30 items are rated on a 7-point scale (1 = absent; 2 = minimal; 3 = mild; 4 = moderate; 5 = moderate severe; 6 = severe; 7 = extreme) that will be combined to form five subscales: a) Positive Symptoms (P1 Delusions, G9 Unusual thought content, P3 Hallucinatory behaviour, P6 Suspiciousness and persecution, P5 Grandiosity), b) Negative Symptoms (N2 Emotional withdrawal, N1 Blunted affect, N4 Passive apathetic social withdrawal, N6 Lack of spontaneity, N3 Poor rapport, G7 Motor retardation, G16 Active social avoidance), c) Disorganization often termed Cognitive (P2 Conceptual disorganization, G11 Poor attention, N5 Difficulty in abstract thinking, G13 Disturbance of volition, N7 Stereotyped thinking, G5 Mannerisms/posturing, G15 Preoccupation, G10 Disorientation), d) Affect often termed Depression-Anxiety (G2 Anxiety, G6 Depression, G3 Guilt feelings, G4 Tension, G1 Somatic concern) and e) Resistance or Excitement/Activity (P7 Hostility, G14 Poor impulse control, P4 Excitement, G8 Uncooperativeness) [7].

The items will also be combined to obtain a PANSS Total Score that can also be used to measure and categorise illness severity [8].

**Scoring:** The five subscales are scored by adding up the corresponding items within each subscale, which will produce subscale scores ranging from 4 to 28, 5 to 35, 7 to 49 and 8 to 56 for the resistance, positive symptoms and affective, negative symptoms and disorganisation subscales respectively, with higher scores indicating more severe symptoms.

The PANSS Total Score is obtained by adding up the 30 items producing a score ranging from 30 to 210, with higher scores indicating more severe symptoms. PANSS baseline total scores between 58 and 74 corresponds to "mildly ill", between 75 and 94 to "moderately ill", between 95 and 115 to "markedly ill" and between 116 and 210 to severely ill. (NB We did not set an “illness” criteria for entry to trial but in relation to PANSS, to be experiencing at least mild hallucinations or delusions symptoms – scoring a 3 on P1 or P3 on PANSS – see inclusion criteria).

An improvement score will also be computed for 16-week and 28-week outcomes using the formula [9]:

$$\frac{{(PANSS}_{baselinetotalscore}-{PANSS}_{endpointtotalscore}) x 100}{\left( {PANSS}_{baselinetotalscore}-30 \right)}$$

**Missing items:** Missing items within subscales will be imputed using a mean of the completed items if no more than 35% items within each subscale are missing; no more than 2 missing item for the negative symptoms and disorganisation subscales and 1 missing item for the positive symptoms, affect and resistance subscales.

### Psychotic Symptom Rating Scales (PSYRATS)

The PSYRATS is a 17-item therapist-administered instrument designed to quantify the severity of delusions and hallucinations, each item being rated from 0 (absent) to 4 (severe).

The PSYRATS has 2 subscales: the auditory hallucinations subscale (AHS) consisting of 11 items (i.e., Frequency, Duration, Location, Loudness, Beliefs Re: Origin, Amount of negative content of voices, Degree of negative content, Amount of distress, Intensity of distress, Disruption to life caused by voices and Controllability of voices) and the delusions subscale (DS) consisting of 6 items (i.e., Amount of preoccupation, Duration of preoccupation, Conviction, Amount of Distress, Intensity of Distress and Disruption) [10].

**Scoring:** Each of the subscales are scored by adding up the items within each subscale, which will yield a score ranging from 0 to 44 for the AHS subscale and from 0 to 24 for the DS, with higher scores indicating higher severity of hallucinations and delusions respectively.

**Missing items:** Missing items within subscales will be imputed using a mean of the completed items if no more than 35% items within each subscale are missing (i.e., 3 and 2 missing items for the AHS and DS subscales respectively).

### Questionnaire about the Process of Recovery (QPR)

The QPR is a 15-item self-reported measure of personal recovery. Each item is scored using a 5-point Likert scale ranging from 0 (disagree strongly) to 4 (agree strongly) [11].

**Scoring:** A total score ranging from 0 to 60 is obtained by summing all the items, with high scores indicating better progress towards recovery goals.

**Missing items:** Missing items will be imputed using a mean of the completed items if no more than 35% items (i.e. 5 items) are missing.

***Imagery***

### Mental Imagery in Psychosis Questionnaire/Visual Analogue Scales (MIPQ/VAS)

The MIPQ is an instrument used to measure mental imagery. For each image, the participants will complete 7 items rated on a visual analogue scale ranging from 1=not at all to 10=extremely [12].

**Scoring:** A total score ranging from 7 to 50 is obtained by summing items 1 to 5, with higher scores indicating more problematic imagery. Items 6 and 7 will be reported separately.

**Missing items:** Missing items 1 to 5 will be imputed using a mean of the completed items if no more than 1 item is missing (i.e., 35% items).

### Psychosis Imagery Questionnaire (PIQ)

This tool is not a secondary outcome for iMAPS-2. Validation of the new measure will be performed after the trial is completed, analysis and results of the validation study will be reported elsewhere.

***Schemas***

### Brief Core Schema Scale (BCSS)

The BCSS aims to provide a theoretically coherent self-report assessment of schemata concerning self and others in psychosis. It consists of 24 items concerning beliefs about the self and others that are assessed on a 4-point Likert scale (Believe it: 1=slightly, 2=moderately, 3=very much and 4=totally). Four domains are obtained: negative-self (items 1 to 6), positive-self (items 7 to 12), negative-others (items 13 to 18) and positive-others (items 19 to 24). The participant is asked to indicate in a dichotomous No/Yes format whether they held each belief. Then, if they held the belief (i.e. answered yes), they are asked to indicate their degree of belief conviction by circling a number from 1 to 4. If they do not hold the belief, a score of zero is assigned [13].

**Scoring:** Each domain is scored by adding the items within each domain, which will yield a score ranging from 0 to 24 with higher scores indicating higher negative/positive evaluation of self/others.

**Missing items:** Missing items within domains will be imputed using a mean of the completed items if no more than 35% items within each domain are missing (i.e., 2 missing items).

***Trauma***

### International Trauma Questionnaire (ITQ)

The ITQ is an 18-item self-report measure with each item rated on a 5-point Likert scale ranging from 0 (Not at all) to 4 (Extremely) focusing on the core features of Post-Traumatic Stress Disorder (PTSD) and Complex PTSD (CPTSD). The ITQ has two major subscales with three symptom clusters in each:

1. Post-Traumatic Stress Disorder (PTSD); Re-experiencing, Avoidance AND Sense of threat.
2. Disturbances in self-organization (DSO); Affective dysregulation, Negative self-concept and Disturbances in relationships.

The PTSD and DSO symptoms are accompanied by three items measuring associated functional impairments in the domains of social, occupation, and other important areas of life [14].

**Scoring:** There are two components of scoring and interpretation for this tool; Categorical scoring for the diagnosis of PTSD and CPTSD, and a dimensional component which measures symptom severity. iMAPS-2 is concerned with the dimensional component only.

The PTSD (items 1 to 6) and DSO (items 10 to 15) subscales range from 0 to 24 and they are computed by summing the items within each subscale, with higher scores indicating more severe symptoms. A total score for CPTSD ranging from 0 to 48 can be obtained by summing the 12 items comprising the PTSD and DSO subscales, with higher scores indicating more severe symptoms.

**Missing items:** Missing items for the PTSD and DSO subscales will be imputed using a mean of the completed items within each subscale if no more than 35% items are missing (i.e., 2 items per subscale). A total score for CPTSD will only be computed if there are valid scores for PTSD and DSO subscales, after item imputation if necessary.

### Basic Emotions Scale (BES)

The BES is a self-report measure comprising 20 items each rated on a 7-point Likert scale ranging from 1 (never) to 7 (very often) that can be combined to form 5 subscales: anger (items 1, 6, 11 and 16), sadness (items 2, 7, 12 and 17), disgust (items 3, 8, 13 and 18), anxiety (items 4, 9, 14 and 19), and happiness (items 5, 10, 15 and 20).

**Scoring:** Each subscale is obtained by summing their corresponding item scores which yield subscales ranging from 4 to 28 with higher scores indicating higher frequency of emotions experienced [15].

**Missing items:** Missing items within subscales will be imputed using the mean of the completed items in each subscale, if no more than 35% of the items (i.e.,1 item) within each subscale are missing.

***Mental Health and Functioning***

### Beck Anxiety Inventory (BAI)

The BAI is a 21-item self-report instrument in which patients rate their experience of the severity of each symptom of anxiety. Each items is rated on a 4-point Likert scale with the anchors of 0 (Not at All) to 3 (Severe) [16].

**Scoring:** A total score, ranging from 0 to 63, is obtained by adding each of the item scores, with higher scores indicating higher levels of anxiety.

**Missing items:** Missing items will be imputed using a mean of the completed items if no more than 35% items (i.e. 7 items) are missing.

### Calgary Depression Scale (CDSS)

The CDSS is a 9-item clinician rated outcome measure that assesses the level of depression in people with schizophrenia. Each item is rated on 3-point Likert-scale (0 = absent; 1 = mild; 2 = moderate; 3 = severe) [17].

**Scoring:** A total score, ranging from 0 to 27, is obtained by adding each of the item scores, with higher scores indicating higher levels of depression.

**Missing items:** Missing items will be imputed using a mean of the completed items if no more than 35% items (i.e. 3 items) are missing.

### Warwick Edinburgh Mental Well Being Scale (WEMWBS)

WEMWBS is a self-reported instrument measuring subjective well-being focusing entirely on positive aspects of mental health. It is composed of 14-items each rated on 5-point Likert scale (1 = none of the time; 2 = rarely; 3 = some of the time; 4 = often; 5 = all of the time) [18].

**Scoring:** A total score ranging from 14 to 70 is obtained by summing the item responses of all 14 items, with higher scores indicating greater positive mental wellbeing.

**Missing items:** Missing items will be imputed using a mean of the completed items if no more than 35% items (i.e. 4 items) are missing.

### The Personal and Social Performance Scale (PSP)

The PSP is a therapist-reported instrument evaluating the degree of difficulty a participant exhibits over a 1-month period in four domains: (a) personal and social relationships; (b) socially useful activities, including work and study; (c) self-care; and (d) disturbing and aggressive behaviours. The patient’s degree of severity in the four domains is rated on a six-point scale from absent (which means no problems on this dimension) over mild, manifest, marked, severe to very severe difficulties in the given area.

[19].

**Scoring:** Out of the ratings on the four domains, a total score on a 100-point scale is computed by the therapist according to manual instructions, with higher scores indicating better personal and social functioning.

**Missing items:** Due to the nature of the PSP scoring is not feasible to impute missing domains to obtain a total score. If a participant declines to answer part of the interview relating to a particular domain, then the PSS will not be scored.

### Working Alliance Inventory Short form (WAI-SR)

This is a rating scale designed to measure the working alliance between therapists and their clients during therapy sessions. The client version is composed of 12 items each rated on a 5-point Likert scale (1 = Seldom; 2 =Sometimes; 3 = Fairly often; 4 = Very often; 5 = Always) on three domains: a) Goal: client and therapist agree on goals of treatment (items 4, 6, 8 and 11), b) Task: whether they agree on how to achieve those goals (items 1, 2, 10 and 12) and c) Bond: whether they will be able to or have already established a personal bond with each other (items 3, 5, 7 and 9).

The therapist version is composed of 10 items also rated on a 1-5 point scale on 3 domains: Goal (items 3, 6 and 8), Task (1, 4 and 10) and Bond subscales (2, 5, 7 and 9).

**Scoring:** A total score is obtained be adding all 12 items producing a scale ranging from 12 to 60, with higher scores indicating greater alliance in the client version.

For the therapist version a total score is obtained be adding all 10 items producing a scale ranging from 10 to 50 with higher scores indicating greater alliance.

**Missing items:** Missing items within domains will be imputed using the mean of the completed items in each domain, if no more than 35% of the items (i.e.,1 item) within each domain are missing for the client and therapist versions. A total score will be computed, for the client and therapist versions, if no more than 1 missing item per domain is missing.

***Adverse Effects***

### Clinical Global Impression- Improvement (CGI-I)

The CGI I is a stand-alone measure rating the change from baseline to treatment. The CGI I is rated on a 7-point scale: 1=very much improved since the initiation of treatment; 2=much improved; 3=minimally improved; 4=no change from baseline (the initiation of treatment); 5=minimally worse; 6= much worse; 7=very much worse since the initiation of treatment [20].

Participant and therapist versions will be reported separately.

### Clinical Global Impression - Severity (CGI-S) Version

The CGI S is a stand-alone measure rating illness severity. The CGI S is rated on a 7-point scale: 1=normal, not at all ill; 2=borderline mentally ill; 3=mildly ill; 4=moderately ill; 5=markedly ill; 6=severely ill; 7=among the most extremely ill patients [20].

Participant and therapist versions will be reported separately.

## Analysis methods

**Objective 1.** What number and percentage of eligible patients/service users consent to the trial (recruitment)?

**Outcomes**

- Number of patients referred to the study.
- Number of patients (potential participants) contacted
- Number of patients screened for eligibility.
- Number of patients screened but not eligible, along with reasons for non-eligibility or withdrawal of interest.
- Number of eligible patients.
- Number of eligible patients not recruited, along with reasons for non-recruitment
- Number of participants recruited (consented).
- Source of recruitment
- Number of participants randomised.

**Analysis**

The number of people screened, eligible, consented and randomised will be summarised and reported in a CONSORT flow diagram (see section 6).

Randomisation rates to assess the progression criteria to conduct a definitive trial will be presented as point estimates with (exact binomial) 95% confidence intervals.

Monthly randomisation rates will also be presented overall and tabulated by randomised group as stated in section 6.3

**Progression criteria**

Progression criteria for recruitment is:

- Green (progress to full trial). At least 80% of target recruited (n ≥ 36)
- Amber (full trial considered feasible if reasons for poor recruitment identified and can be addressed). Between 40% and <80% of target recruited (n between 18 and 35, inclusive)
- Red (unlikely to progress to full trial. Substantial amendments will be necessary to make a full trial feasible). Below 40% of target recruited (n<18).

**Objective 2.** What is the level of engagement with adherence to the iMAPS-2 intervention (therapy sessions attendance measures; therapist fidelity)?

**Outcome**

Participant adherence

- Number of sessions of iMAPS-2 therapy attended for those participants allocated to the intervention arm.

Therapist fidelity:

- The Cognitive Therapy Rating Scale Revised (CTS-R) or
- The Cognitive Therapy Rating Scale for Psychosis (CTS-Psy)
- Therapist checklist.

**Analysis**

Assessment of participant’s adherence and therapist fidelity are fully described in section 5.2.1.

The proportion of participants allocated to the intervention arm, attending at least 5 sessions of the iMAPS-2 therapy will be presented as point estimates with 95% (exact binomial) confidence intervals to assess the progression criteria to conduct a definitive trial.

In addition, we will report the proportion of participants whom have received at least 5 hours (300 minutes) of the iMAPS-2 therapy along with a 95% (exact binomial) CI. We will also summarise, as mean(SD) or median(IQR) as appropriate, the overall hours of iMAPS-2 therapy received, and the hours of iMAPS-2 therapy received by the adherent participants (those who received at least 5 sessions).

Finally, we will plot:

- A histogram showing the distribution of iMAPS-2 therapy sessions attended (whether they are part or whole sessions).
- A histogram showing the distribution of hours of iMAPS-2 therapy sessions attended.

**Progression criteria**

Progression criteria for participant adherence is:

- Green (progress to full trial). At least 75% of the participants allocated to the intervention arm attend at least 5 sessions of iMAPS-2 therapy.
- Amber (full trial considered feasible if reasons for poor participant’s adherence identified and can be addressed). 40% - <75% of the participants allocated to the intervention arm attend at least 5 sessions of iMAPS-2 therapy.
- Red (unlikely to progress to full trial. Substantial amendments will be necessary to make a full trial feasible). If less than 40% of the participants allocated to the intervention arm attend at least 5 sessions of iMAPS-2 therapy.

**Objective 3.** What completion and data quality rates can be achieved (data completion and retention of participants)?

**Outcome**

- Number of participants continuing in the trial at 16 weeks.
- Number of participants completing the trial at 28 weeks.
- Questionnaire completion rates (after missing item imputation) at baseline, 16 weeks and 28 weeks for the following instruments:
  - Positive and Negative Syndrome Scale (PANSS)
  - Psychotic Symptom Rating Scales (PSYRATS)
  - Questionnaire about the Process of Recovery (QPR)
  - Mental Imagery in Psychosis Questionnaire/Visual Analogue Scales Visual Analogue Scales (MIPQ)
  - Brief Core Schema Scale (BCSS)
- Number of completed items per questionnaire as listed in the previous bullet point and prior to missing item imputation.

**Analysis**

The number of participants attending each of the 16- and 28-week trial visits will be summarised and reported in a CONSORT flow diagram (see section 6.3). In addition, retention rates at 16 weeks to assess the progression criteria to conduct a definitive trial, and also at 28 weeks, will be presented as point estimates with 95% (exact binomial) confidence intervals.

We will summarise the frequency (%, with denominator the number attending the corresponding visit) of the completed instruments (after missing item imputation) listed above by time point. We will also report the number of completed items per instrument (prior to missing item imputation) at each time point, and frequency (%) of valid scale (and subscale) scores for each instrument.

If any of the candidate primary outcome measures shows a significant amount of missing item data (more than 5% of partially or fully completed questionnaires), we will present the % of missing data by item to identify problematic items or sets of items.

**Progression criteria**

Progression Criteria for retention (baseline to primary outcome completion at 16-weeks) is:

- Green (progress to full trial). At least 80% retention:
- Amber (full trial considered feasible if reasons for poor retention identified and can be addressed). 60%-<80% retention
- Red (unlikely to progress to full trial. Substantial amendments will be necessary to make a full trial feasible). Below 60% retention.

**Objective 4.** What estimates of effect sizes (if any) are present (acknowledging this is a feasibility trial)?

**Analysis**

We shall analyse each of the candidate primary outcomes (i.e., Positive and Negative Syndrome Scale (PANSS) Total score, Psychotic Symptom Rating Scales (PSYRATS), Questionnaire about the Process of Recovery (QPR) and Brief Core Schema Scale (BCSS), using a separate linear regression model at each time-point to estimate the effect sizes at 16 and 28 weeks.

Each model will be fitted to the clinical outcome measure at the respective assessment time-point (16 or 28 weeks) and will include the fixed-effects for treatment therapy (iMAPS-2+TAU or TAU), care team type (CMHT or EI) and the corresponding baseline outcome score.

Models will be fitted using Maximum Likelihood Estimation (MLE).

Point estimates will be presented as regression coefficients and a range of confidence intervals at different confidence levels (75%, 80%, 85%, 90% & 95%) will be structured and examined as whether they are likely to capture the minimal clinically important difference (MID) for the primary end-point (i.e., 16 weeks). Point estimates with 95% confidence intervals will be presented at 28 weeks.

For this purpose, the MID will, in general, be a SES of 0.3, and so the confidence intervals used for comparison with the MID will likewise be standardised using the overall baseline SD. However, for the PANSS Total Score, the confidence intervals for the effect estimates on the unstandardised scale will also be compared with the proposed MID of 5 points.

These analyses will be used to consider evidence as to potential proof of effectiveness and will also provide estimates of their SD to assist with the estimation of the required sample size for a full effectiveness trial (Objective 6), should that measure be retained for consideration as primary. We will report the estimated standardised effect size (SES)= Effect estimate / SD_pooled_ with their corresponding 95%CIs calculated dividing the limits of the 95%CI of the effect estimate by the SD_pooled_.

The remaining continuous outcomes listed in section 7.1 will be analysed in the same fashion than the candidate primate outcomes. Each regression model will be fitted to the clinical outcome measure at the respective assessment time-point (16 or 28 weeks) and will include the fixed-effects for treatment therapy (iMAPS-2+TAU or TAU), care team type (CMHT or EI) and the corresponding baseline outcome score. Estimates of the treatment effects will be presented as the mean difference with 95% CIs.

***Regression diagnostics***

Diagnostic checks to assess the appropriateness of the regression models fitted will be through the use of residual plots. We will plot:

- Histograms and probability plots of the residuals to assess normality
- Scatter plots of residuals against fitted values to assess constant variance and linearity, and to identify potential outliers.

Should the normality assumption be untenable for the continuous outcomes, bootstrap methods will be applied to estimate the confidence intervals of the treatment effects.

**Objective 6.** What is the estimated sample size for a fully powered trial to evaluate the effectiveness of iMAPS-2 (relative to usual care)?

**Outcomes**

Positive and Negative Syndrome Scale (PANSS), Psychotic Symptom Rating Scales (PSYRATS), Questionnaire about the Process of Recovery (QPR) and Brief Core Schema Scale (BCSS)

**Analysis**

The analyses described for Objective 4, together with the findings of the qualitative analysis that will consider the views of the participants on the candidate primary outcome measures, will be used to ‘shortlist’ a potential primary outcome measure(s). We will also consider the feasibility of running a full effectiveness trial with each of those candidate outcomes remaining after this process, by considering the respective sample sizes required to detect a specified MID to assist with our decision-making. We will discuss our findings and proposals with the Trial Steering Committee to help inform our recommendations for a full effectiveness or evaluation trial.

### Sensitivity analyses

Follow-up outcome assessments are performed at 16 weeks (end-of-therapy assessment) and 28 weeks (follow-up assessment), although there is no formal time window to maximise completion of outcome data as stated in section 4.8. From a feasibility perspective to report the frequency (%) of participants completing the assessments within a reasonable timeframe (i.e., within 4 weeks of the scheduled time-points).

If the proportion of scheduled assessments completed more than 4 weeks after the scheduled date for any time-point is ≥10%, we will repeat the efficacy analyses for objective 4 excluding the ‘out of window’ assessments. This will help to explore the consideration of data collection and practicalities around introducing a ‘completion window’ for a full trial.

## Subgroup analyses

N/A

## Missing data

As this is a feasibility study we will focus on reporting the amount of missing data (see sections 6.4). We will report the number (%) of participants withdrawing fully from the trial and lost to follow-up by treatment arm. Reasons for missingness may be important and these will be tabulated by treatment arm and documented as far as possible.

We will also tabulate the number of missing items within questionnaires (see Objective 3, section 7.2).

Efficacy analyses of clinical outcomes are adjusted for baseline values. If there are any missing baseline values of the corresponding outcome data, we will use simple mean imputation (across the groups) to avoid exclusion of such participants in the proposed complete-case analysis [21].

## Additional analyses

There are 3 additional analyses planned:

1. We will tabulate the PANSS response rates:

- Deterioration or no change (Negative or 0% reduction from baseline)
- >0% but <25% reduction from baseline
- 25% to <50% reduction from baseline
- 50% to <75% reduction from baseline
- 75% to <100% reduction from baseline
- 100% reduction from baseline

at 16 and 28 weeks by treatment arm to show how many participants are still symptomatic at the end of treatment and follow up, and how many have responded and to show the overall amount of change.

1. To assess the extent of unblinding, we will report the frequency (%) of recorded instances where outcomes assessors reported to be unblinded, overall and by randomised group.
2. We will summarise the individual items of the brief imagery interview (shortened version of Schulze et al. , 2013) where participants describe the images (from 1 image to 3 images) they experienced in relation to your psychosis:
3. Image frequency in past week (0 = images have not occurred 1 = images occur once a week; 2 = images occur a few times a week; 3 = images occur once a day; 4 = images occur multiple times a day)
4. Image frequency in past month 0 = images have not occurred 1 = images occur once a week; 2 = images occur a few times a week; 3 = images occur once a day; 4 = images occur multiple times a day
5. Image Distress (0-100) where 0 is not distressing at all and 100 is the most distressing it could be.

We will tabulate items 1 and 2 and present frequencies (%) of the endorsed individual items. Images reported and Image distress will be summarised by mean (SD) or median (IQR) as appropriate.

Results will be presented by allocated treatment and time point (i.e., Baseline, 16 weeks and 28 weeks).

1. The reporting of harms, as described in the next section.

## Harms

The number of safety events (e.g., AEs and SAEs) and number (and proportion) of participants reporting at least one safety event will be summarised by treatment arm. Summary measures will be number (%).

Safety events will also be tabulated by:

- Death by suicide
- Suicide attempt
- Suicidal crisis without attempt (rating 2 on item 8 of Calgary Depression Rating Scale for Schizophrenia CDSS)
- Severe symptoms increase (rating of > 6 on the patient or researcher rated CGI and CGI-I)

They will be reported by both overall and by treatment arm.

Treatment effects will be estimated by the difference in event rates and 95% CI for the differences.

In addition, we will tabulate and present frequencies of the endorsed individual items in the Adverse Effects in Psychotherapy (AEP) scale (29) for those participants allocated to the intervention arm. This is a self-report measure of potential adverse effects completed by the participants at the end of therapy assessment measuring 7 broad categories: worsening difficulties, poor engagement (including low motivation), situational change, no benefit from therapy, stigma, conflict with others (family, care team) and feeling better.

## Health economic analysis

The aim of the health economic analysis is to establish the optimum way of evaluating cost-effectiveness in a full trial. The focus is on informing the data required for a definitive trial, including health status measurement and the range of costs to be included.

### Health status (EQ-5D) and Quality-Adjusted Life-Years (QALYs)

To assess the feasibility of collecting the EQ-5D measure in a full trial, we will report and review the completeness of the data, as well as an assessment of whether the measure appears to be reflecting health in the population. EQ-5D index values/utility values will be derived using the approach recommended by NICE, which is currently using the validated mapping function from the existing EQ-5D-3L. In line with current NICE recommendations, the mapping function developed by the Decision Support Unit (DSU) using the 'EEPRU dataset' will be used [22].

The key questions that will be answered are below:

- How complete is the EQ-5D data? This will be assessed by reporting the proportion of participants with partially (e.g., missing a domain) or completely missing EQ-5D responses.
- Are the utilities as expected compared to general population norms for a similar age and gender mix? This will be assessed by comparing the estimating utility values to published population norms [25].
- How do the EQ-5D index values correlate to key measures and demographics? This will be assessed by direction, strength of correlation and statistical significance using a simple exploratory regression analysis.

The described analysis will provide some early conclusions on the suitability of the EQ-5D for future trials and will also give some information on the likely moderators or influencers of total QALYs, to inform data collection in the definitive trial.

Recognising some of the limitations of the EQ-5D, the ReQoL-10 (a newer and more mental health focused measure) has also been collected within the feasibility analysis and will be used to generate alternative utility scores using a published algorithm [24]. The completeness of data will again be assessed to identify whether participants found the measure easier or more challenging to complete in comparison to the EQ-5D. In addition, the generated utility scores (EQ-5D and ReQoL) will be compared to assess whether one measure may be more or less suitable.

### Health and social care use

As part of the feasibility trial a health service resource use questionnaire is collecting participant reported health and social care use (hospital, primary, community and social care use). An analysis of the range of services used and ability of participants to report complete service use data will be used to inform a definitive trial.

Data from the resource use questionnaire will be cleaned and costed. Total costs will be presented, and mean costs will be reported for each type of service use, alongside standard deviation and 95% confidence intervals. The unit costs of NHS and social care services will be derived from national average unit cost data. These unit costs are published annually in the NHS reference costs database, and in the Unit Costs of Health and Social Care document published by the Personal Social Services Research Unit (PSSRU), University of Kent.

The key questions that will be answered are below:

- Was the questionnaire feasible for participants and researchers to complete? This will be assessed with a summary of missing data (by type of service) to assess the level of missingness and whether any sections were difficult to complete. Any unfeasible values or outliers will be discussed with the research team. Discussions will be held with the researchers to get their perspective of the questionnaire. This will focus on whether simplifications can be made and whether the recall period is appropriate.
- Would revisions to the health resource use questionnaire be required for a definitive trial? If so, what are these? Following the findings above, suggestions will be made to revise service use collection forms.

### Intervention costs

Costs of providing the iMAPS intervention will be estimated using the staff time to deliver intervention and number of sessions.

### Exploratory cost-effectiveness analysis

An exploratory cost-effectiveness acceptability analysis may be conducted. However, this will be limited due to the feasibility stage of the work (in particular the sample size) and it should be highlighted that the key aims of the economic analysis are not to assess cost-effectiveness, rather to aid the design of a definitive trial. If the data are sufficient (i.e., if complete cost and QALY data are available for >75% of participants), an exploratory cost-effectiveness analysis will be performed.

Analysis of the economic data will use an intention-to-treat approach and will consider complete cases only. This analysis will use only the observed data and will provide insight to the result for the group of participants with complete follow up and complete data (evaluable cohort). The proportion of participants for which complete QALY and cost data are available will be summarised. In a definitive trial, imputation techniques would be applied to overcome some of the impact of missing data, however, this exploratory analysis will be kept simple to not overstate the usefulness of the analysis.

Costs and health benefit for the primary analysis will be estimated from baseline to end of follow-up, to estimate the incremental cost-effectiveness of the addition of iMAPS intervention. The primary measure of interest for the economic analysis is the incremental cost-effectiveness ratio (ICER). This is calculated by dividing the difference in costs (net costs) by the difference in QALYs (net QALYs) between any two interventions. The ICER represents the additional cost associated with an intervention per additional QALY gained:

ICER =

Cost _iMAPS intervention plus TAU_ – Cost _TAU_

QALYs _iMAPS intervention plus TAU_ – QALYs _TAU_

Regression analysis will be used to estimate the net costs and QALYs of iMAPS intervention. Key covariates (aligned with the statistical analysis plan) will be included in the regression models to control for baseline factors that may influence QALYs or costs. The covariates for these analyses will be identified in the analysis described above. The estimates of costs and health benefits from the regression analyses will be bootstrapped to simulate 10,000 pairs of incremental cost and QALY outcomes of the intervention. Pairs of net costs and QALYs will be plotted on a cost-effectiveness plane to illustrate the level of uncertainty in the data.

Finally, each of the net QALY estimates from bootstrap simulation results will be revalued by multiplying it by a willingness to pay threshold to estimate the net benefit statistic. The monetary value of simulated QALYs will be varied from £0 to £30,000 to reflect a range of hypothetical willingness to pay thresholds (WTPT).

## Statistical software

All analyses will be performed using STATA/SE (StataCorp, College Station, TX, USA).

# References

| [1] | S. A. Julious, “Sample size of 12 per group rule ofthumb for a pilot study,” *Pharmaceutical Statistics,* vol. 4, no. 4, p. 287–291, 2005. |
| --- | --- |
| [2] | J. Sim and M. Lewis, “The size of a pilot study for a clinical trial should be calculated in relation to considerations of precision and efficiency,” *Journal of Clinical Epidemiology,* vol. 65, no. 3, pp. 301-308, 2012. |
| [3] | E. C. Lee, A. L. Whitehead, R. M. Jacques and S. A. Julious, “The statistical interpretation of pilot trials: should significance thresholds be reconsidered?,” *BMC medical research methodology,* vol. 14, no. 1, p. 41, 2014. |
| [4] | S. Jolley, P. Garety, E. Peters , . M. Fornells-Ambrojo, J. Onwumere, . V. Harris and A. Brab, “Opportunities and challenges in Improving Access to Psychological Therapies for people with Severe Mental Illness (IAPT-SMI): evaluating the first operational year of the South London and Maudsley (SLaM) demonstration site for psychosis,” *Behaviour Research and Therapy,* vol. 64, pp. 24-30, 2015. |
| [5] | K. F. Schulz, D. G. Altman and D. Moher, “CONSORT 2010 Statement: updated guidelines for reporting parallel group randomised trials,” *BMJ,* p. 340:c332 , 2010. |
| [6] | S. M. Eldridge, C. L. Chan, M. J. Campbell, C. M. Bond, S. Hopewell, L. Thabane and G. . A. Lancaster, “CONSORT 2010 statement: extension to randomised pilot and feasibility trials,” *BMJ,* p. 355:i5239, 2016. |
| [7] | A. Shafer and F. Dazzi, “Meta-analysis of the positive and Negative Syndrome Scale (PANSS) factor structure,” *Journal of Psychiatric Research,* vol. 115, pp. 113-120, 2019. |
| [8] | M. van der Gaag, T. Hoffman, M. Remijsen, R. Hijman, L. de Haan, B. van Meijel, P. N. van Harten, L. Valmaggia, M. de Hert, A. Cuijpers and D. Wiersma, “The five-factor model of the Positive and Negative Syndrome Scale II: A ten-fold cross-validation of a revised model,” *Schizophrenia Research,* vol. 85, no. 1-3, pp. 280-7, 2005. |
| [9] | S. Leucht , J. Davis , R. Engel , W. Kissling and J. Kane , “Definitions of response and remission inschizophrenia: recommendations for theiruse and their presentation,” *Acta Psychiatrica Scandinavica,* vol. 119, no. (Suppl. 438), p. 7–14, 2009. |
| [10] | G. Haddock, J. McCarron, N. Tarrier and E. B. Faragher, “Scales to measure dimensions of hallucinations and delusions: the psychotic symptom rating scales (PSYRATS),” *Psychological Medicine,* vol. 29, no. 4, pp. 879-89, 1999. |
| [11] | H. Law , S. T. Neil, G. Dunn and A. P. Morrison, “Psychometric properties of the Questionnaire about the Process of Recovery (QPR),” *Schizophrenia Research,* vol. 156, no. 2-3, pp. 184-9, 2014. |
| [12] | C. D. Taylor, P. E. Bee, J. Kelly and G. Haddock, “iMAgery-Focused Psychological Therapy for Persecutory Delusions in PSychosis (iMAPS): A Novel Treatment Approach,” *Cognitive and Behavioral Practice,* vol. 26, no. 3, pp. 575-588, 2019. |
| [13] | D. Fowler, D. Freeman, B. Smith, E. Kuipers, P. Bebbington, H. Bashforth, S. Coker, J. Hodgekins, A. Gracie, G. Dunn and P. Garety, “The Brief Core Schema Scales (BCSS): psychometric properties and associations with paranoia and grandiosity in non-clinical and psychosis samples,” *Psychological Medicine,,* vol. 36, no. 6, pp. 749-759, 2003. |
| [14] | M. Cloitre, M. Shevlin, C. R. Brewin, J. I. Bisson, N. P. Roberts, A. Maercker, T. Karatzias and P. Hylan, “The International Trauma Questionnaire: development of a self-report measure of ICD-11 PTSD and complex PTSD,” *Acta Psychiatrica Scandinavica,* vol. 138, no. 6, pp. 536-546, 2018. |
| [15] | M. J. Power, “The structure of emotion: An empirical comparison of six models,” *Cognition and Emotion,* vol. 20, no. 5, p. 694–713, 2006. |
| [16] | A. T. Beck, N. Epstein, G. Brown and R. A. Steer, “An inventory for measuring clinical anxiety: psychometric properties,” *Consulting and Clinical Psychology,* vol. 56, no. 6, pp. 893-7, 1988. |
| [17] | D. Addington, J. Addington and E. Maticka-tyndale, “Assessing depression in schizophrenia: the Calgary Depression Scale,” *The British Journal of Psychiatry,* vol. 163, no. S22, pp. 39-44, 1993. |
| [18] | R. Tennant, L. Hiller, R. Fishwick, S. Joseph, S. Weich, J. Parkinson, J. Secker and S. Stewart-Brown, “The Warwick-Edinburgh Mental Well-being Scale (WEMWBS): development and UK validation,” *Health Qual Life Outcomes,* vol. 5, no. 1, p. 63, 2007. |
| [19] | P. L. Morosini, L. Magliano, L. Brambilla and S. Ugoli, “Development, reliability and acceptability of a new version of the DSM-IV Social and Occupational Functioning Assessment Scale (SOFAS) to assess routine social functioning,” *Acta Psychiatrica Scandinavica,* vol. 101, no. 4, p. 323–329., 2001. |
| [20] | J. Busner and S. D. Targum, “The clinical global impressions scale: applying a research tool in clinical practice,” *Psychiatry (edgmont),* vol. 4, no. 7, p. 28–37, 2007. |
| [21] | I. R. White and S. G. Thompson, “Adjusting for partially missing baseline measurements in randomized trials,” *Statistics in Medicine,* vol. 24, p. 993–1007, 2004. |
| [22] | “The manual Process and methods,” NICE health technology evaluation (PMG36), 2022. |
| [23] | B. Janssen, A. Szende and J. Cabases, “Population Norms for the EQ-5D,” in *Self-Reported Population Health: An International Perspective based on EQ-5D*, Dordrecht (NL), Springer, 2014, p. Chapter 3. |
| [24] | A. D. Keetharuth, D. Rowen, J. B. Bjorner and J. Brazier, “Estimating a Preference-Based Index for Mental Health From the Recovering Quality of Life Measure: Valuation of Recovering Quality of Life Utility Index,” *Value Health,* vol. 24, no. 2, pp. 281-290, 2021. |
| [25] | S. R. Kay, A. Fiszbein and L. A. Opler, “The Positive and Negative Syndrome Scale (PANSS) for Schizophrenia,” *Schizophrenia Bulletin,* vol. 13, no. 2, p. 261–276, 1987. |
| [26] | S. Carr, A. Hardy and M. Fornells-Ambrojo, “The Trauma and Life Events (TALE) checklist: development of a tool for improving routine screening in people with psychosis,” *European Journal of Psychotraumatology,* vol. 9, no. 1, p. Article: 1512265, 2018. |
| [27] | J. Williams, M. Leamy, F. Pesola, V. Bird, C. Le Boutillier and M. Slade, “Psychometric evaluation of the Questionnaire about the Process of Recovery (QPR),” *The British Journal of Psychiatry,* vol. 207, no. 6, p. 551–555, 2015. |
| [28] | Rothwell, J C, S. A. Julious and C. L. Cooper, “A study of target effect sizes in randomised controlled trials published in the Health Technology Assessment Journal,” *Trials,* vol. 19, no. 1, p. 544, 2018. |

[29] Pyle, M., Norrie, J., Schwannauer, M., Kingdon, D., Gumley, A., Turkington, D., Byrne, R., Syrett, S., MacLennan, G., Dudley, R., MacLeod, H. J., Griffiths, H., Bowe, S., Barnes, T. R. E., French, P., Hutton, P., Davies, L., and Morrison, A. P. (2016). "Design and protocol for the Focusing on Clozapine Unresponsive Symptoms (FOCUS) trial: a randomised controlled trial", *BMC Psychiatry*, vol. 16, 280, p1-12.

# APPENDIX

**Figure 1:** CONSORT Diagram

**
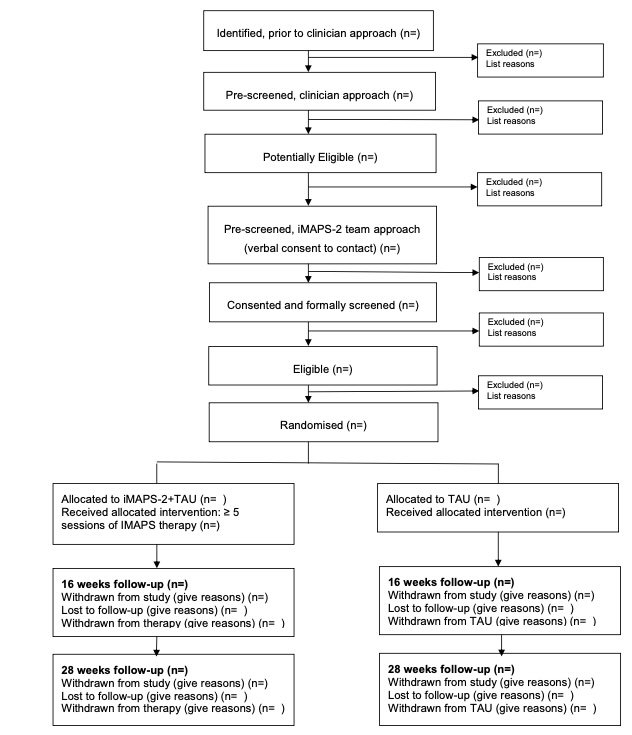
**

**Additional Tables**

**Monthly and cumulative randomisation**

We were interested in the number and percentage of eligible patients/service users consenting to the trial (recruitment).

Participant recruitment began on 14^th^ July 2022 and the final participant was randomised on 08^th^ September 2023. The study recruited its target of 45 participants randomised, 100%, 95%CI (92% to 100%) and therefore met the green criteria for recruitment. Monthly and cumulative randomised figures are shown in the table below. The overall average monthly rate randomised was 45/15 = 3 participants per month.

**Supplementary Table 1: Monthly and cumulative randomisation**

| **Month** | **Randomised** |
| --- | --- |
|  |  |
| 14^th^ July 2022 | 0 |
| Aug-22 | 1 |
| Sep-22 | 0 |
| Oct-22 | 2 |
| Nov-22 | 5 |
| Dec-22 | 3 |
| Jan-23 | 4 |
| Feb-23 | 6 |
| Mar-23 | 3 |
| Apr-23 | 5 |
| May-23 | 5 |
| Jun-23 | 3 |
| Jul-23 | 1 |
| Aug-23 | 3 |
| Sep-23 | 4 |

**Supplementary Figure 1: Distribution of the number of therapy sessions attended and their duration in minutes**

The proportion of participants who began therapy and received at least 300 minutes (5 hours) of the iMAPS therapy was 23/29 (79%; 95%CI - 55% to 88%), with a mean (SD) and median of 495 (228) and 581 (350 to 660) of therapy minutes received respectively

**Supplementary Table 2: Baseline Trauma and Life Events (TALE) by treatment allocated**

|  |  | **iMAPS+TAU** | **TAU** | **Total** |
| --- | --- | --- | --- | --- |
| **Trauma Category** |  | **n=31** | **n=14** | **N=45** |
| Trauma type (TALE item number) | War exposure (item 1) | 2 (6) | 1 (7) | 3 (7) |
|  | Attachment related (2-4) | 28 (90) | 11 (79) | 39 (87) |
|  | Witnessed verbal or physical abuse at home(item 9) | 17 (55) | 5 (36) | 22 (49) |
|  | Any interpersonal (any of below items) |  |  |  |
|  | Bullying & discrimination (5 & 6) | 23 (74) | 11 (79) | 34 (76) |
|  | - Sexual abuse (13 & 14) | 14 (45) | 7 (50) | 21 (47) |
|  | - Emotional abuse (item 7) | 23 (74) | 8 (57) | 31 (69) |
|  | - Physical abuse (8 & 10) | 19 (61) | 4 (29) | 23 (51) |
|  | - Emotional neglect (item 11) | 17 (55) | 4 (29) | 21 (47) |
|  | - Physical neglect (item 12) | 4 (13) | 2 (14) | 6 (13) |
|  | Psychosis-related (15-17) | 30 (97) | 13 (93) | 43 (96) |
|  | Criminal justice (item 18) | 10 (32) | 3 (21) | 13 (29) |
|  | Non-interpersonal (item 19) | 7 (23) | 5 (36) | 12 (27) |
|  | Other trauma (item 20) | 8 (26) | 5 (36) | 13 (29) |
| Perceived impact (TALE item 21.c) - Impact of index traumas on current difficulties | Mean (SD) | 8 (2) | 8 (1) | 8 (2) |
|  | Range | 2 to 10 | 6 to 10 | 2 to 10 |
|  | Median (IQR) | 8 (7 to 10) | 8 (7 to 9) | 8 (7 to 10) |
| Multiple exposure | Repeated events (at least 1 item answered ‘yes’ to more than once) | 25 (81) | 13 (93) | 38 (84) |
|  | Multiple trauma types (more than 1 of above types reported) | 31 (100) | 13 (93) | 44 (98) |
| No. of trauma types | Mean (SD) | 9 (3) | 8 (5) | 9 (4) |
|  | Range | 3 to 15 | 1 to 17 | 1 to 17 |
|  | Median (IQR) | 9 (6 to 12) | 9 (3 to 12) | 9 (6 to 12) |
| Trauma timing | Child (any item <16 years old) | 25 (81) | 9 (64) | 34 (76) |
|  | Adult (any item >16 years old) | 29 (94) | 13 (93) | 42 (93) |
|  | both (any item <16 yr. AND >16 yrs.) | 11 (35) | 6 (43) | 17 (38) |

**Supplementary Table 3: Baseline clinical measures by treatment allocated**

|  |  | **iMAPS+TAU** | **TAU** | **Total** |
| --- | --- | --- | --- | --- |
| **Positive and Negative Syndrome Scale (PANSS)** | |  |  |  |
| Positive Symptoms | Mean (SD) | 15.6 (4.0) | 17.1 (2.7) | 16.1 (3.7) |
|  | Range | 9.0 to 25.0 | 13.0 to 24.0 | 9.0 to 25.0 |
|  | IQR | 15.0 (13.0 to 18.0) | 16.5 (16.0 to 18.0) | 16.0 (13.0 to 18.0) |
|  | n | 31 | 14 | 45 |
| Negative Symptoms | Mean (SD) | 15.0 (4.9) | 13.3 (3.0) | 14.4 (4.4) |
|  | Range | 7.0 to 24.0 | 8.0 to 19.0 | 7.0 to 24.0 |
|  | IQR | 15.0 (11.0 to 19.0) | 13.5 (11.0 to 15.0) | 14.0 (11.0 to 17.0) |
|  | n | 31 | 14 | 45 |
| Disorganization | Mean (SD) | 13.5 (3.7) | 13.1 (3.3) | 13.4 (3.5) |
|  | Range | 8.0 to 27.0 | 10.0 to 18.0 | 8.0 to 27.0 |
|  | IQR | 13.0 (11.0 to 15.0) | 12.0 (10.0 to 17.0) | 13.0 (11.0 to 16.0) |
|  | n | 31 | 14 | 45 |
| Affect | Mean (SD) | 14.8 (3.3) | 16.9 (2.7) | 15.4 (3.2) |
|  | Range | 9.0 to 22.0 | 12.0 to 24.0 | 9.0 to 24.0 |
|  | IQR | 15.0 (12.0 to 17.0) | 17.0 (15.0 to 18.0) | 16.0 (13.0 to 17.0) |
|  | n | 31 | 14 | 45 |
| Resistance | Mean (SD) | 4.6 (1.4) | 4.9 (1.5) | 4.7 (1.4) |
|  | Range | 4.0 to 9.0 | 4.0 to 9.0 | 4.0 to 9.0 |
|  | IQR | 4.0 (4.0 to 4.0) | 4.0 (4.0 to 6.0) | 4.0 (4.0 to 4.0) |
|  | n | 31 | 14 | 45 |
| Total score | Mean (SD) | 65.4 (11.5) | 66.6 (9.1) | 65.8 (10.7) |
|  | Range | 45.0 to 101.0 | 51.0 to 79.0 | 45.0 to 101.0 |
|  | IQR | 64.0 (58.0 to 72.0) | 66.5 (61.0 to 75.0) | 64.0 (58.0 to 73.0) |
|  | n | 31 | 14 | 45 |
| **Psychotic Symptom Rating Scales (PSYRATS)** | |  |  |  |
| Auditory Hallucinations Subscale (AHS) | Mean (SD) | 13.9 (14.1) | 18.5 (13.5) | 15.3 (13.9) |
|  | Range | 0.0 to 37.0 | 0.0 to 36.0 | 0.0 to 37.0 |
|  | IQR | 12.0 (0.0 to 30.0) | 23.0 (0.0 to 30.0) | 16.0 (0.0 to 30.0) |
|  | n | 31 | 14 | 45 |
| Delusions Subscale (DS) | Mean (SD) | 13.8 (5.2) | 15.9 (3.2) | 14.5 (4.8) |
|  | Range | 0.0 to 22.0 | 9.0 to 21.0 | 0.0 to 22.0 |
|  | IQR | 14.0 (11.0 to 18.0) | 16.0 (15.0 to 17.0) | 15.0 (12.0 to 18.0) |
|  | n | 31 | 14 | 45 |
| **Questionnaire about the Process of Recovery (QPR)** | Mean (SD) | 28.7 (11.5) | 27.7 (9.5) | 28.4 (10.8) |
|  | Range | 2.0 to 46.0 | 10.0 to 43.9 | 2.0 to 46.0 |
|  | IQR | 28.5 (20.0 to 39.0) | 27.5 (23.0 to 35.0) | 28.5 (23.0 to 39.0) |
|  | n | 30 | 14 | 44 |
| **Mental Imagery in Psychosis Questionnaire (MIPQS)** | |  |  |  |
| Image 1 | Mean (SD) | 34.4 (14.0) | 38.2 (6.7) | 35.6 (12.2) |
|  | Range | 5.0 to 50.0 | 24.0 to 50.0 | 5.0 to 50.0 |
|  | IQR | 39.5 (26.0 to 45.0) | 38.0 (34.0 to 41.0) | 39.0 (31.5 to 45.0) |
|  | n | 30 | 14 | 44 |
| Image 2 | Mean (SD) | 36.3 (13.5) | 39.5 (6.8) | 37.1 (12.1) |
|  | Range | 5.0 to 50.0 | 26.0 to 45.0 | 5.0 to 50.0 |
|  | IQR | 42.0 (23.8 to 47.0) | 41.5 (41.0 to 42.0) | 42.0 (26.0 to 47.0) |
|  | n | 17 | 6 | 23 |
| Image 3 | Mean (SD) | 37.0 (12.8) | 12.5 (10.6) | 30.9 (16.2) |
|  | Range | 13.0 to 47.0 | 5.0 to 20.0 | 5.0 to 47.0 |
|  | IQR | 42.0 (33.0 to 45.0) | 12.5 (5.0 to 20.0) | 36.5 (16.5 to 44.5) |
|  | n | 6 | 2 | 8 |
| **Brief Core Schema Scale (BCSS)** |  |  |  |  |
| Negative-self | Mean (SD) | 8.8 (7.4) | 9.4 (7.2) | 9.0 (7.3) |
|  | Range | 0.0 to 23.0 | 0.0 to 22.0 | 0.0 to 23.0 |
|  | IQR | 7.0 (2.0 to 17.0) | 9.5 (2.0 to 15.0) | 8.0 (2.0 to 15.6) |
|  | n | 31 | 14 | 45 |
| Positive-self | Mean (SD) | 8.4 (7.0) | 8.1 (4.5) | 8.3 (6.3) |
|  | Range | 0.0 to 20.0 | 0.0 to 15.0 | 0.0 to 20.0 |
|  | IQR | 5.5 (2.0 to 16.3) | 8.0 (6.0 to 11.0) | 6.5 (2.0 to 15.0) |
|  | n | 28 | 14 | 42 |
| Negative-others | Mean (SD) | 9.9 (7.4) | 9.3 (8.1) | 9.7 (7.5) |
|  | Range | 0.0 to 24.0 | 0.0 to 24.0 | 0.0 to 24.0 |
|  | IQR | 9.0 (4.0 to 16.0) | 6.0 (4.0 to 15.0) | 9.0 (4.0 to 15.5) |
|  | n | 31 | 13 | 44 |
| Positive-others | Mean (SD) | 8.3 (6.9) | 9.5 (6.0) | 8.7 (6.6) |
|  | Range | 0.0 to 24.0 | 0.0 to 24.0 | 0.0 to 24.0 |
|  | IQR | 8.0 (3.0 to 14.0) | 8.0 (6.0 to 13.0) | 8.0 (3.0 to 14.0) |
|  | n | 29 | 14 | 43 |
| **International Trauma Questionnaire (ITQ)** | |  |  |  |
| Post-Traumatic Stress Disorder (PTSD) | Mean (SD) | 14.6 (6.4) | 12.2 (5.4) | 13.8 (6.2) |
|  | Range | 2.0 to 24.0 | 2.0 to 22.0 | 2.0 to 24.0 |
|  | IQR | 15.0 (9.0 to 20.0) | 12.0 (9.0 to 15.0) | 14.0 (9.0 to 18.5) |
|  | n | 27 | 13 | 40 |
| Disturbances in self- organization (DSO) | Mean (SD) | 15.6 (6.5) | 12.5 (4.9) | 14.6 (6.2) |
|  | Range | 2.0 to 24.0 | 2.0 to 21.0 | 2.0 to 24.0 |
|  | IQR | 16.0 (11.0 to 22.0) | 12.0 (11.0 to 15.0) | 15.3 (11.0 to 20.0) |
|  | n | 27 | 13 | 40 |
| Total Score | Mean (SD) | 30.2 (12.4) | 24.7 (9.7) | 28.4 (11.7) |
|  | Range | 4.0 to 48.0 | 4.0 to 40.0 | 4.0 to 48.0 |
|  | IQR | 31.0 (24.0 to 40.0) | 23.0 (20.0 to 29.0) | 28.3 (20.5 to 37.0) |
|  | n | 27 | 13 | 40 |
| **Basic Emotions Scale (BES)** |  |  |  |  |
| Anger | Mean (SD) | 16.6 (5.5) | 15.6 (4.6) | 16.3 (5.2) |
|  | Range | 6.0 to 28.0 | 9.0 to 24.0 | 6.0 to 28.0 |
|  | IQR | 16.0 (13.0 to 19.0) | 14.5 (13.0 to 19.0) | 16.0 (13.0 to 19.0) |
|  | n | 31 | 14 | 45 |
| Sadness | Mean (SD) | 15.3 (5.9) | 14.4 (6.2) | 15.0 (5.9) |
|  | Range | 5.0 to 27.0 | 6.0 to 25.0 | 5.0 to 27.0 |
|  | IQR | 15.0 (10.0 to 20.0) | 13.5 (10.0 to 19.0) | 14.0 (10.0 to 20.0) |
|  | n | 31 | 14 | 45 |
| Disgust | Mean (SD) | 16.0 (6.6) | 15.7 (7.2) | 15.9 (6.7) |
|  | Range | 5.0 to 27.0 | 5.0 to 28.0 | 5.0 to 28.0 |
|  | IQR | 17.0 (10.0 to 22.0) | 14.0 (11.0 to 22.0) | 16.0 (11.0 to 22.0) |
|  | n | 31 | 14 | 45 |
| Anxiety | Mean (SD) | 20.4(6.1) | 21.1 (5.0) | 20.6 (5.8) |
|  | Range | 10.0to28.0 | 13.0 to 28.0 | 10.0 to 28.0 |
|  | IQR | 23.0(14.0to26.0) | 20.0 (17.0 to 26.0) | 21.0 (16.0 to 26.0) |
|  | n | 31 | 14 | 45 |
| Happiness | Mean (SD) | 14.8(4.6) | 15.8 (3.0) | 15.1 (4.2) |
|  | Range | 6.0to27.0 | 11.0 to 22.0 | 6.0 to 27.0 |
|  | IQR | 15.0(12.0to17.0) | 15.5 (14.0 to 18.0) | 15.0 (12.0 to 17.0) |
|  | n | 31 | 14 | 45 |
| **Beck Anxiety Inventory (BAI)** | Mean (SD) | 30.3 (16.2) | 31.3 (14.6) | 30.6 (15.5) |
|  | Range | 6.0 to 58.0 | 5.0 to 56.0 | 5.0 to 58.0 |
|  | IQR | 30.0 (16.0 to 43.0) | 31.0 (21.0 to 41.0) | 30.0 (18.0 to 43.0) |
|  | n | 31 | 14 | 45 |
| **Calgary Depression Scale (CDS))** | Mean (SD) | 9.7 (5.2) | 9.9 (3.5) | 9.8 (4.7) |
|  | Range | 2.0 to 22.0 | 6.0 to 18.0 | 2.0 to 22.0 |
|  | IQR | 9.0 (6.0 to 13.0) | 9.0 (7.0 to 12.0) | 9.0 (7.0 to 13.0) |
|  | n | 31 | 14 | 45 |
| **Warwick Edinburgh Mental Well Being Scale (WEMWBS)** | Mean (SD) | 37.0 (9.8) | 36.6 (8.1) | 36.9 (9.2) |
|  | Range | 22.0 to 62.0 | 21.0 to 51.0 | 21.0 to 62.0 |
|  | IQR | 38.0 (28.0 to 45.0) | 36.0 (33.0 to 42.0) | 36.6 (30.0 to 44.0) |
|  | n | 31 | 14 | 45 |
| **The Personal and Social Performance Scale (PSP)** | Mean (SD) | 58.0 (9.8) | 56.2 (8.1) | 57.5 (9.3) |
|  | Range | 38.0 to 79.0 | 40.0 to 68.0 | 38.0 to 79.0 |
|  | IQR | 57.0 (50.0 to 65.0) | 55.0 (52.0 to 61.0) | 57.0 (50.5 to 65.0) |
|  | n | 31 | 13 | 44 |

**Supplementary Table 4a: Post Hoc Analysis - Frequency (%) of participants by time-point with presence of both auditory hallucinations & delusions, hallucinations or delusions only at baseline**

|  | **Baseline** | | **16 Weeks** | | **28 Weeks** | |
| --- | --- | --- | --- | --- | --- | --- |
|  | **iMAPS-TAU** | **TAU** | **iMAPS-TAU** | **TAU** | **iMAPS-TAU** | **TAU** |
|  | **n=31** | **n=14** | **n=25** | **n=11** | **n=18** | **n=8** |
| Auditory hallucinations & delusions | 14 (45) | 10 (71) | 10 (40) | 7 (64) | 9 (50) | 3 (38) |
| Missing | 0 (0) | 0 (0) | 1 (4) | 0 (0) | 0 (0) | 2 (25) |
| Auditory hallucinations only | 2 (6) | 0 (0) | 2 (8) | 0 (0) | 2 (11) | 0 (0) |
| Missing | 0 (0) | 0 (0) | 1 (4) | 0 (0) | 1 (6) | 2 (25) |
| Delusions only | 15 (48) | 4 (29) | 12 (48) | 3 (27) | 7 (39) | 3 (38) |
| Missing | 0 (0) | 0 (0) | 1 (4) | 1 (9) | 1 (6) | 2 (25) |

**Supplementary Table 4b: Post Hoc Analysis Baseline summary measures of the Psychotic Symptom Rating Scales (PSYRATS) for those participants with auditory hallucinations & delusions at baseline**

|  |  | **iMAPS+TAU**  **n=14** | **TAU**  **n=10** | **Total**  **N=24** |
| --- | --- | --- | --- | --- |
| **Psychotic Symptom Rating Scales (PSYRATS)** | |  |  |  |
| Auditory Hallucinations Subscale (AHS) | Mean (SD) | 27.1 (6.1) | 25.9 (7.0) | 26.6 (6.4) |
|  | Range | 14.0 to 37.0 | 14.0 to 36.0 | 14.0 to 37.0 |
|  | Median (IQR) | 29.0 (26.0 to 31.0) | 27.5 (21.0 to 30.0) | 28.5 (23.0 to 31.0) |
|  | Mean (SD) | 15.6 (4.1) | 16.5 (2.8) | 16.0 (3.5) |
| Delusions Subscale (DS) | Range | 7.0 to 22.0 | 11.0 to 21.0 | 7.0 to 22.0 |
|  | Median(IQR) | 16.0 (13.0 to 18.0) | 16.5 (15.0 to 17.0) | 16.5 (13.5 to 18.0) |

**Supplementary Table 4c: Post Hoc Analysis Follow up summary measures of the Psychotic Symptom Rating Scales (PSYRATS) for those participants with auditory hallucinations & delusions at baseline**

|  |  | **16 Weeks** | | | **28 Weeks** | | |
| --- | --- | --- | --- | --- | --- | --- | --- |
|  |  | **iMAPS+TAU**  **n=10** | **TAU**  **n=7** | **Total**  **N=17** | **iMAPS+TAU**  **n=9** | **TAU**  **n=3** | **Total**  **N=12** |
| **Psychotic Symptom Rating Scales (PSYRATS)** | |  |  |  |  |  |  |
| Auditory Hallucinations Subscale (AHS) | Range | 0.0 to 31.0 | 0.0 to 39.0 | 0.0 to 39.0 | 0.0 to 33.0 | 0.0 to 26.4 | 0.0 to 33.0 |
|  | Mean(SD) | 23.0 (10.4) | 18.9 (18.0) | 21.3 (13.7) | 20.9 (13.3) | 8.8 (15.2) | 17.6 (14.2) |
|  | Median(IQR) | 27.5 (16.0 to 31.0) | 27.0 (0.0 to 34.0) | 27.0 (13.0 to 31.0) | 26.0 (11.5 to 29.5) | 0.0 (0.0 to 26.4) | 25.0 (0.0 to 28.0) |
|  | Missing | 0 | 0 | 0 | 1 | 0 | 1 |
| Delusions Subscale (DS) | Range | 0.0 to 21.0 | 5.0 to 19.0 | 0.0 to 21.0 | 0.0 to 17.0 | 5.0 to 20.0 | 0.0 to 20.0 |
|  | Mean(SD) | 12.2 (6.2) | 14.6 (5.1) | 13.2 (5.7) | 5.1 (7.2) | 12.3 (7.5) | 7.1 (7.7) |
|  | Median(IQR) | 13.5 (7.0 to 17.0) | 16.0 (12.0 to 19.0) | 14.0 (12.0 to 17.0) | 0.0 (0.0 to 12.0) | 12.0 (5.0 to 20.0) | 5.0 (0.0 to 12.0) |
|  | Missing | 0 | 0 | 0 | 1 | 0 | 1 |

**Supplementary Table 4d: Post Hoc Analysis Treatment effects at 16 weeks of the Psychotic Symptom Rating Scales (PSYRATS) for those participants with auditory hallucinations & delusions at baseline**

|  | **Scale range** | **N** | **Effect estimate** | **in favour of …** | **95% CI** | **90% CI** | **85% CI** | **80% CI** | **75% CI** | **SD_pooled_** | **SES (95%CI)** |
| --- | --- | --- | --- | --- | --- | --- | --- | --- | --- | --- | --- |
| **Psychotic Symptom Rating Scales (PSYRATS)** | |  |  |  |  |  |  |  |  |  |  |
| Auditory Hallucinations Subscale (AHS) | 0-44 | 17 | -3.4 | iMAPs | (-17.4 to 10.6) | (-15.1 to 8.3) | (-13.1 to 6.4) | (-12.5 to 5.7) | (-11.4 to 4.7) | 13.9 | -0.2 (-1.2 to 0.8) |
| Delusions Subscale (DS) | 0-24 | 17 | 1.3 | TAU | (-3.3 to 6.0) | (-2.5 to 5.2) | (-2.2 to 4.9) | (-1.8 to 4.4) | (-1.5 to 4.2) | 5.8 | 0.2 (-0.6 to 1.0) |

*Effect estimate = iMAPS intervention+TAU - TAU*

*Standardised effect size (SES)=* *Effect estimate /* SD_pooled_; the corresponding 95%CI is calculated by dividing the limits of the 95%CI for the effect estimate by the SD_pooled_.

**Supplementary Table 5: Response rates as measured by the Positive and Negative Syndrome Scale (PANSS) by treatment allocated and time point.**

|  | **N (%)** | | | |
| --- | --- | --- | --- | --- |
|  | **16 Weeks** | | **28 Weeks** | |
|  | **iMAPs** | **TAU** | **iMAPs** | **TAU** |
| **Improvement category** | **n=25** | **n=11** | **n=18** | **n=8** |
| Deterioration or no change | 8 (32) | 5 (45) | 8 (44) | 2 (25) |
| >0% but <25% reduction from baseline | 7 (28) | 3 (27) | 4 (22) | 1 (13) |
| 25% to <50% reduction from baseline | 6 (24) | 3 (27) | 2 (11) | 1 (13) |
| 50% to <75% reduction from baseline | 2 (8) | 0 (0) | 1 (6) | 1 (13) |
| 75% to <100% reduction from baseline | 0 (0) | 0 (0) | 1 (6) | 0 (0) |
| Missing | 2 (8) | 0 (0) | 2 (11) | 3 (38) |

**Supplementary Table 6:** Number of Adverse Events and Serious Adverse Events by Treatment Group and Overall

|  | **iMAPS + TAU** | **TAU** | **Overall** |
| --- | --- | --- | --- |
| **Unrelated** |  |  |  |
| Total Serious Adverse Events (SAE), n  Hospital Admission  Physical  Mental Health/Psychiatric  Suicide Attempts | 1  1  0 | 0  0  0 | 2 |
| Total Adverse Events (AE), n  Suicidal Ideation with a plan | 8 | 3 | 11 |
| **Total** | 10 | 3 | 13 |
| **Related** |  |  |  |
| Total Serious Adverse Events (SAE), n  Hospital Admission  Physical  Mental Health/Psychiatric  Suicide Attempts | 0  0  0 | 0  0  0 | 0  0  0 |
| Total Adverse Events (AE), n  Suicidal Ideation with a plan | 0 | 0 | 0 |
| **Total** | 0 | 0 | 0 |
|  |  |  |  |
|  |  |  |  |

**Supplementary Table 7a: Image 1 - How often have you experienced the image in the past week?**

|  | **N (%)** | | | | | |
| --- | --- | --- | --- | --- | --- | --- |
|  | **Baseline** | | **16 Weeks** | | **28 Weeks** | |
|  | **iMAPs** | **TAU** | **iMAPs** | **TAU** | **iMAPs** | **TAU** |
| Images have not occurred | 4 (13) | 0 (0) | 8 (32) | 2 (18) | 9 (50) | 3 (38) |
| Occur once a week | 4 (13) | 0 (0) | 2 (8) | 1 (9) | 2 (11) | 1 (13) |
| A few times a week | 7 (23) | 3 (21) | 5 (20) | 3 (27) | 2 (11) | 0 (0) |
| Once a day | 16 (52) | 8 (57) | 2 (8) | 4 (36) | 2 (11) | 0 (0) |
| Multiple times a day | 0 (0) | 2 (14) | 3 (12) | 1 (9) | 2 (11) | 2 (25) |
| n | 31 | 13 | 20 | 11 | 17 | 5 |

**Supplementary Table 7b: Image 1 - How often have you experienced the image in the past month?**

|  | **N (%)** | | | | | |
| --- | --- | --- | --- | --- | --- | --- |
|  | **Baseline** | | **16 Weeks** | | **28 Weeks** | |
|  | **iMAPs** | **TAU** | **iMAPs** | **TAU** | **iMAPs** | **TAU** |
| images have not occurred | 0 (0) | 0 (0) | 6 (24) | 0 (0) | 8 (44) | 2 (25) |
| occur once a month | 1 (3) | 0 (0) | 1 (4) | 1 (9) | 2 (11) | 1 (13) |
| a few times a month | 5 (16) | 1 (7) | 3 (12) | 2 (18) | 0 (0) | 1 (13) |
| at least once a week | 9 (29) | 3 (21) | 4 (16) | 4 (36) | 3 (17) | 0 (0) |
| once a day | 14 (45) | 6 (43) | 2 (8) | 3 (27) | 2 (11) | 0 (0) |
| multiple times a day | 0 (0) | 3 (21) | 3 (12) | 1 (9) | 2 (11) | 2 (25) |
| N | 29 | 13 | 19 | 11 | 17 | 6 |

**Supplementary Table 7c: Image 1 How distressing was the image at the time?** (Higher scores show worse distress)

|  | **N (%)** | | | | | |
| --- | --- | --- | --- | --- | --- | --- |
|  | **Baseline** | | **16 Weeks** | | **28 Weeks** | |
|  | **iMAPs** | **TAU** | **iMAPs** | **TAU** | **iMAPs** | **TAU** |
| Mean (SD) | 78 (17) | 78 (17) | 62 (25) | 48 (27) | 56 (21) | 65 (31) |
| Range | 50 to 100 | 50 to 100 | 25 to 100 | 6 to 100 | 10 to 90 | 30 to 100 |
| Median (IQR) | 75 (60 to 90) | 80 (65 to 90) | 65 (40 to 80) | 40 (40 to 70) | 60 (50 to 65) | 65 (40 to 90) |
| n | 30 | 14 | 15 | 9 | 9 | 4 |

**Supplementary Table 8a: Image 2 - How often have you experienced the image in the past week?**

|  | **N (%)** | | | | | | |
| --- | --- | --- | --- | --- | --- | --- | --- |
|  | **Baseline** | | | **16 Weeks** | | **28 Weeks** | |
|  | **iMAPs** | | **TAU** | **iMAPs** | **TAU** | **iMAPs** | **TAU** |
| images have not occurred | 4 (20) | 1 (11) | | 5 (25) | 2 (33) | 8 (57) | 2 (33) |
| occur once a week | 7 (35) | 2 (22) | | 1 (5) | 2 (33) | 1 (7) | 0 (0) |
| a few times a week | 3 (15) | 2 (22) | | 5 (25) | 0 (0) | 4 (29) | 1 (17) |
| once a day | 4 (20) | 4 (44) | | 2 (10) | 2 (33) | 0 (0) | 0 (0) |
| multiple times a day | 0 (0) | 0 (0) | | 2 (10) | 0 (0) | 0 (0) | 1 (17) |
| n | 18 | 9 | | 15 | 6 | 13 | 4 |

**Supplementary Table 8b: Image 2 - How often have you experienced the image in the past month?**

|  | **N (%)** | | | | | | |
| --- | --- | --- | --- | --- | --- | --- | --- |
|  | **Baseline** | | | **16 Weeks** | | **28 Weeks** | |
|  | **iMAPs** | | **TAU** | **iMAPs** | **TAU** | **iMAPs** | **TAU** |
| images have not occurred | 1 (5) | 0 (0) | | 4 (20) | 2 (33) | 6 (43) | 2 (33) |
| occur once a monthk | 0 (0) | 1 (11) | | 1 (5) | 0 (0) | 0 (0) | 0 (0) |
| a few times a month | 4 (20) | 2 (22) | | 4 (20) | 1 (17) | 3 (21) | 0 (0) |
| at least once a week | 8 (40) | 2 (22) | | 2 (10) | 1 (17) | 4 (29) | 0 (0) |
| once a day | 4 (20) | 4 (44) | | 2 (10) | 2 (33) | 0 (0) | 1 (17) |
| multiple times a day | 0 (0) | 0 (0) | | 2 (10) | 0 (0) | 0 (0) | 0 (0) |
| n | 17 | 9 | | 15 | 6 | 13 | 3 |

**Supplementary Table 8c: Image 2 How distressing was the image at the time?** (Higher scores show worse distress)

|  | **N (%)** | | | | | | |
| --- | --- | --- | --- | --- | --- | --- | --- |
|  | **Baseline** | | **16 Weeks** | | | **28 Weeks** | |
|  | **iMAPs** | **TAU** | **iMAPs** | **TAU** | | **iMAPs** | **TAU** |
| Mean (SD) | 76 (17) | 84 (11) | 58 (24) | 42 (31) | 46 (31) | | 33 (4) |
| Range | 50 to 100 | 65 to 100 | 18 to 100 | 6 to 60 | 6 to 85 | | 30 to 35 |
| Median (IQR) | 80 (70 to 85) | 85 (80 to 90) | 60 (40 to 80) | 60 (6 to 60) | 60 (15 to 70) | | 33 (30 to 35) |
| n | 17 | 9 | 11 | 3 | 7 | | 2 |

**Supplementary Table 9a: Image 3 - How often have you experienced the image in the past week?**

|  | **N (%)** | | | | | | |
| --- | --- | --- | --- | --- | --- | --- | --- |
|  | **Baseline** | | | **16 Weeks** | | **28 Weeks** | |
|  | **iMAPs** | | **TAU** | **iMAPs** | **TAU** | **iMAPs** | **TAU** |
| images have not occurred | 0 (0) | 1 (25) | | 2 (18) | 2 (50) | 2 (29) | 2 (40) |
| occur once a week | 3 (38) | 0 (0) | | 0 (0) | 1 (25) | 1 (14) | 0 (0) |
| a few times a week | 0 (0) | 0 (0) | | 4 (36) | 0 (0) | 3 (43) | 0 (0) |
| once a day | 4 (50) | 2 (50) | | 0 (0) | 0 (0) | 0 (0) | 0 (0) |
| multiple times a day | 0 (0) | 1 (25) | | 0 (0) | 1 (25) | 0 (0) | 0 (0) |
| n | 7 | 4 | | 6 | 4 | 6 | 2 |

**Supplementary Table 9b: Image 3 - How often have you experienced the image in the past month?**

|  | **N (%)** | | | | | | |
| --- | --- | --- | --- | --- | --- | --- | --- |
|  | **Baseline** | | | **16 Weeks** | | **28 Weeks** | |
|  | **iMAPs** | | **TAU** | **iMAPs** | **TAU** | **iMAPs** | **TAU** |
| images have not occurred | 0 (0) | 1 (25) | | 2 (18) | 2 (50) | 2 (29) | 1 (20) |
| occur once a monthk | 0 (0) | 0 (0) | | 0 (0) | 0 (0) | 0 (0) | 1 (20) |
| a few times a month | 1 (13) | 0 (0) | | 0 (0) | 0 (0) | 0 (0) | 0 (0) |
| at least once a week | 2 (25) | 0 (0) | | 1 (9) | 1 (25) | 3 (43) | 0 (0) |
| once a day | 4 (50) | 2 (50) | | 2 (18) | 0 (0) | 1 (14) | 0 (0) |
| multiple times a day | 0 (0) | 1 (25) | | 0 (0) | 1 (25) | 0 (0) | 0 (0) |
| n | 7 | 4 | | 5 | 4 | 6 | 2 |

**Supplementary Table 9c: Image 3 How distressing was the image at the time?** (Higher scores show worse distress)

|  | **N (%)** | | | | | |
| --- | --- | --- | --- | --- | --- | --- |
|  | **Baseline** | | **16 Weeks** | | **28 Weeks** | |
|  | **iMAPs** | **TAU** | **iMAPs** | **TAU** | **iMAPs** | **TAU** |
| Mean (SD) | 81 (23) | 68 (43) | 82 (13) | 50 (.) | 71 (10) | 40 (.) |
| Range | 50 to 100 | 20 to 100 | 70 to 95 | 50 to 50 | 60 to 85 | 40 to 40 |
| Median (IQR) | 90 (50 to 100) | 85 (20 to 100) | 80 (70 to 95) | 50 (50 to 50) | 70 (65 to 78) | 40 (40 to 40) |
| n | 7 | 3 | 3 | 1 | 4 | 1 |

**Supplementary Table 10: Working Alliance Inventory (WAI) Scores – Participant and Therapist Versions**

|  | **iMAPS + TAU (n = 31)** | | |
| --- | --- | --- | --- |
|  | **M** | **S.D.** | **Missing** |
| WAI - Participant |  |  |  |
| Session 3 | 45.0 | 9.5 | 20 |
| Session 6 | 55.7 | 6.1 | 24 |
| Session 9 | 47.0 | 8.6 | 21 |
| WAI – Therapist |  |  |  |
| Session 3 | 39.9 | 1.9 | 22 |
| Session 6 | 41.6 | 4.8 | 24 |
| Session 9 | 42.1 | 2.9 | 19 |

Note: WAI measures were only administered to participants who were randomised to the iMAPS + TAU arm. The WAI Participant measure was 12 item version and the WAI Therapist measure was 10 item version.

**Supplementary Table 11: CONSORT Combined reporting checklist**

| **Section** | **CONSORT-PILOT** | **Pp.** | **CONSORT-SPI** | **Pp.** |
| --- | --- | --- | --- | --- |
| **Title and abstract** |  |  |  |  |
| Title | 1a.Identification as a pilot or feasibility randomised trial in the title | 1 |  |  |
| Abstract | 1b. Structured summary of pilot trial, design, methods, results and conclusions (see CONSORT abstract extension for pilot trials) | 2 | 1b.Refer to CONSORT extension for social and psychological intervention trial abstracts | 2 |
| **Introduction** |  |  |  |  |
| Background and rationale | 2a. Scientific background and explanation of rationale for future definitive trial, and reasons for randomised pilot trial | 3 | 2b. If pre-specified, how the intervention was hypothesised to work. | 3 |
| Objectives | 2b. Specific objectives or research questions for pilot trial | 7 |  |  |
| **Methods** |  |  |  |  |
| Trial design | 3a. Description of pilot trial design (such as parallel, factorial) including allocation ratio | 4 |  |  |
|  | 3b. Important changes to methods after pilot trial commencement (such as eligibility), with reasons | N/A |  |  |
| Participants | 4a. Eligibility criteria 4 for participants | 4 |  |  |
|  | 4b. Settings and locations where the data were collected | 4 |  |  |
|  | 4c. How participants were identified and consented | 4 | 4a. Where applicable, eligibility criteria for settings and those delivering the interventions. | 4 |
| Interventions | 5.The interventions for each group with sufficient details to allow replications, including how and when they were actually administered | 6 | 5a. Extent to which interventions were actually delivered by providers and taken up by participants as planned. | 12 |
|  |  |  | 5b Where other informational materials about delivering the intervention can be accessed. | 6 |
|  |  |  | 5c Where applicable, how intervention providers were assigned to each group | N/A |
| Outcomes | 6a. Completely defined prespecified assessments or measurements to address each pilot trial objective specified in 2b, including how and when they were assessed | 7 |  |  |
|  | 6b. Any changes to pilot trial assessments or measurements after the pilot trial commenced, with reasons | N/A |  |  |
|  | 6c. If applicable, prespecified criteria used to judge whether, or how, to proceed with future definitive trial | 7 |  |  |
| Sample size | 7a. Rationale for numbers in the pilot trial | 5 |  |  |
|  | 7b. Where applicable, explanation of any interim analyses and stopping guidelines | N/A |  |  |
| Sequence generation | 8a. Method used to generate the random allocation sequence | 5 |  |  |
|  | 8b. Type of randomisation(s); details of any restriction (such as blocking and block size) | 5 |  |  |
| Allocation concealment mechanism | 9. Mechanism used to implement the random allocation sequence (such as sequentially numbered containers), describing any steps taken to conceal the sequence until interventions were assigned. | 5 |  |  |
| Implementation | 10.Who generated the random allocation sequence, who enrolled participants, and who assigned participants to interventions | 5 |  |  |
| Blinding (masking) | 11a.If done, who was blinded after assignment to interventions (for example, participants care providers, those assessing outcomes) and how | 8 |  |  |
|  | 11b.If relevant, description of similarity of interventions | N/A |  |  |
| Statistical methods | 12a. Methods [which will] used to address each pilot trial objective whether qualitative or quantitative | 10 | 12a. How missing data were handled, with details of any imputation method | 11 |
| **Results** |  |  |  |  |
|  | 13a For each group, the numbers of participants who were approached and/or assessed for eligibility, randomly assigned, received intended treatment and were assessed for each objective | 11,12 | 13a Where possible, the number approached, screened and eligible prior to random assignment, with reasons for non-enrolment | 11,12 |
|  | 13b For each group, losses and exclusions after randomisation, together with reasons | 11,12 |  |  |
|  | 14b. Why the pilot trial ended or was stopped | N/A |  |  |
|  | 15. A table showing baseline demographic and clinical characteristics for each group | 11 | 15. Include socioeconomic variables where appropriate | 11 |
|  | 16. For each objective, number of participants (denominator) included in each analysis. If relevant, these numbers should be by randomised group. | 11 |  |  |
|  | 17.For each objective, results including expressions of uncertainty (such as 96% confidence interval) for any estimates. If relevant, these results should be by randomised group. | 13 | 17a.Indicate the availability of trial data. | 18 |
|  | 18.Results of any other analysis performed that could be used to inform the future definitive trial | N/A |  |  |
|  | 19a.All important harms or unintended effects in each group (for specific guidance see CONSORT for harms) | 14 |  |  |
|  | 19b.If relevant, other important unintended consequences | N/A |  |  |
| **Ethics and dissemination** |  |  |  |  |
| **Discussion** |  |  |  |  |
| Limitations | 20.Pilot trial limitations, addressing sources of potential bias and remaining uncertainty about feasibility | 17 | 20.Trial limitations, addressing sources of potential bias, imprecision, and if relevant, multiplicity of analyses | 16,17 |
| Generalisability | 21.Generalisability (applicability) of pilot trial methods and findings to future definitive trial and other studies | 16 | 21.Generalisablity (external validity, applicability) of the trial findings | 16 |
|  | 22. Interpretation consistent with pilot trial objectives and findings, balancing potential harms and considering other relevant evidence | 17 | 22.Interpretation consistent with results, balancing benefits and harms and considering other relevant evidence | 17 |
|  | 23.Implications for progression from pilot to future definitive trial, including any proposed amendments | 17 |  |  |
| **Administrative information** |  |  |  |  |
| Trial registration | 23.Registration number for pilot trial and name of trial registry | 4 |  |  |
| Protocol | 24.Where the pilot protocol can be accessed, if available | 4 |  |  |
| Funding | 25.Sources of funding and other support (such as supply of drugs), role of funders | 18 |  |  |
| Ethical approval | 26. Ethical approval or approval by research review committee, confirmed with reference number | 4 |  |  |
| Declaration of interests |  |  | 25 Declaration of any other interests | 18 |
| Stakeholder involvement/ investment |  |  | 26a.Any involvement of the intervention developer in the design, conduct, analysis or reporting of the trial. | 18 |
|  |  |  | 26b.Other stakeholder involvement in trial design, conduct, or analyses. | N/A |
|  |  |  | 26c.Inventives offered as part of the trial | N/A |
|  |  |  |  |  |

**Supplementary Table 12: Changes and Amendments submitted for Research Ethics Committee (REC) approval**

|  | **Description** | **Date** |
| --- | --- | --- |
| 1 | Three primary feasibility outcomes were included in the HRA approved Protocol. At trial registration, two additional outcomes were listed: therapy safety (AEs and SAEs) was added and blinding breaks. This was an administrative error (both therapy safety and blinding breaks were mentioned in the protocol and are reported in this outcomes manuscript). | 22/05/2022 |
| 2 | **Substantial Amendment**  1. Reduce Inclusion Criteria Age Range from 18 years to 16 years old, to expand access to a greater number of Early Intervention Psychosis service users/patients.  2. Expand recruitment to include adult inpatient wards, for patients who are judged to have capacity to consent as assessed by a key clinician, to expand access to the study for potentially eligible service users/patients.  3. To capture imagery characteristics each therapy session, as a potential mechanism of change  4. Seek verbal consent to document demographics at screening stage, to record any differences between those screened and those eventually randomised, to support maximising purposive sampling of participants from different ethnic minority backgrounds. | 21/11/2022 |
| 3 | **Substantial Amendment**  Substantial Amendment requested to add a third qualitative study. The aim of the study is explore therapist's experiences of delivering imagery focused therapy through interviews and to amend participant’s qualitative interview schedule. | 20/01/2023 |
| 4 | **Non-Substantial Amendment**  Non-Substantial Amendment to extend study end date to 30^th^ June 2024, to allow 16 week and 28-week assessments to be completed. Recruitment window had been extended due to research assistants leaving post and new colleagues appointed, CI bereavement leave, and delays in contacting wider mental health team for referrals due to MH team staff absences due to Flu season/Ill Health in Winter 2022/23 during recruitment phase. | 27/10/2023 |

1. Mental Imagery “occurs when perceptual information is accessed from memory, giving rise to the experience of “seeing with the mind’s eye”, hearing with the mind’s ear.” (Kosslyn et al. 2001). We define a distressing image as a potential participant reporting an mental image, a “picture in the mind’s eye”, which could also be in any one of the five senses. The images could be clear or unclear, fully formed or fleeting. [↑](#footnote-ref-1)
